# Do large language models differ in their pharmacology-related response quality for oral and maxillofacial surgery? a blinded expert benchmark study

**DOI:** 10.1186/s12903-026-09182-w

**Published:** 2026-07-04

**Authors:** Mustafa Isleyen, Asenur Aydemir

**Affiliations:** Department of Oral and Maxillofacial Surgery, Faculty of Dentistry, Mehmet Akif Ersoy University, Burdur, Turkey

**Keywords:** Large language models, ChatGPT, Gemini, Claude, Oral and maxillofacial surgery, Pharmacology, Drug–drug interactions, Clinical decision support

## Abstract

**Background:**

Pharmacology represents the lowest-performing subcategory in oral and maxillofacial surgery (OMFS) evaluations of large language models (LLMs), yet no study has simultaneously compared the leading commercial LLMs across multiple pharmacological domains and question formats. This study evaluated ChatGPT 5.3, Gemini 3.1 Pro, and Claude 4.6 Sonnet in OMFS pharmacology.

**Methods:**

Thirty-six OMFS pharmacology questions spanning five clinical domains (antibiotic prophylaxis, analgesics, drug–drug interactions, anesthetic pharmacology, special populations) and three formats (open-ended, multiple-choice, true/false; *n* = 12 each) were submitted to each LLM using a standardized role-conditioning prompt. The 108 responses were independently and blindly evaluated by two oral and maxillofacial surgeons (one specialist and one resident) on three 5-point Likert criteria. Inter-rater reliability was quantified using ICC(2,1) and Cohen’s κ_w. Inter-model differences were assessed using Friedman tests; format effects were assessed using Kruskal–Wallis tests with Bonferroni-corrected post-hoc comparisons.

**Results:**

Inter-rater reliability was excellent (ICC = 0.828; κ_w = 0.827; exact agreement 91.0%). A robust hierarchy emerged: Claude > Gemini > ChatGPT (χ²(2) = 47.91, *p* < 0.001, W = 0.665), with all pairwise comparisons significant. Gemini and Claude did not differ significantly in any format section, indicating clinical equivalence. ChatGPT exhibited a significant decline on open-ended, integrative-reasoning items (H(2) = 17.04, *p* < 0.001, ε² = 0.456), absent in Gemini and Claude. Significant positive correlations among the evaluation criteria within the ChatGPT data indicated convergence among the three scoring dimensions.

**Conclusion:**

Claude 4.6 Sonnet and Gemini 3.1 Pro achieved near-maximal scores on this structured pharmacology benchmark, while ChatGPT 5.3 showed a significant decline in open-ended reasoning. Current LLMs should be regarded as adjunctive tools requiring expert verification for high-risk OMFS pharmacological decisions.

**Supplementary Information:**

The online version contains supplementary material available at https://doi.org/10.1186/s12903-026-09182-w.

## Introduction

Since the public release of ChatGPT in 2022, large language models (LLMs) have become a widely consulted source of health information for both patients and clinicians, with population-level studies documenting a marked shift toward conversational AI platforms for medical queries [1, 2]. As LLM-generated clinical content directly influences healthcare decisions, rigorous, domain-specific performance evaluation has become an urgent research priority [3, 4].

In oral and maxillofacial surgery (OMFS), a growing body of research has begun benchmarking LLM performance against discipline-specific knowledge. Quah et al. evaluated five LLMs on 259 OMFS examination questions and reported a mean accuracy of 62.5%, with all models performing poorest in the pharmacology subcategory (45.9%) [5]. Grillo et al. demonstrated variable task-dependent performance across six LLMs [6], and Liu et al. showed that all evaluated models exhibited significant performance decline on clinical case-based reasoning relative to factual recall [7]. Pharmacology occupies a uniquely high-risk position in OMFS practice, where errors in dosing, contraindication recognition, or drug–drug interaction (DDI) management in medically compromised patients can carry serious consequences [8, 9]. De Vito et al. evaluated 14 LLMs across 60 antibiotic prescribing scenarios and found substantial inter-model variability, with older versions of Claude and Gemini yielding the lowest accuracy, underscoring the importance of version-specific benchmarking [10].

Despite this clinical imperative, published evidence on LLM performance in OMFS pharmacology remains limited. Çırakoğlu et al. compared ChatGPT-3.5 and Gemini-1.0 on dental antibiotic questions but reported poor inter-rater reliability (ICC = 0.314 and 0.001) and limited scope to a single domain [11]. Crucially, no study to date has simultaneously benchmarked all three leading current-generation LLMs in OMFS pharmacology. Existing evaluations have largely relied on single-format questions, single-rater scoring, or perception-based survey designs, none of which can detect format-dependent performance differences or quantify evaluator agreement. The present study was designed to address these specific gaps. We conducted a cross-sectional, comparative in silico evaluation of ChatGPT 5.3, Gemini 3.1 Pro, and Claude 4.6 Sonnet on a structured bank of 36 OMFS pharmacology questions spanning five clinical domains. The bank was stratified across three question formats: open-ended, multiple-choice, and true/false. Each response was independently and blindly evaluated by two raters, a specialist oral and maxillofacial surgeon and a resident, using a multi-dimensional, three-criteria, 5-point Likert framework with formal inter-rater reliability quantification.

## Methods

### Study design and ethical considerations

**Fig. 1:**
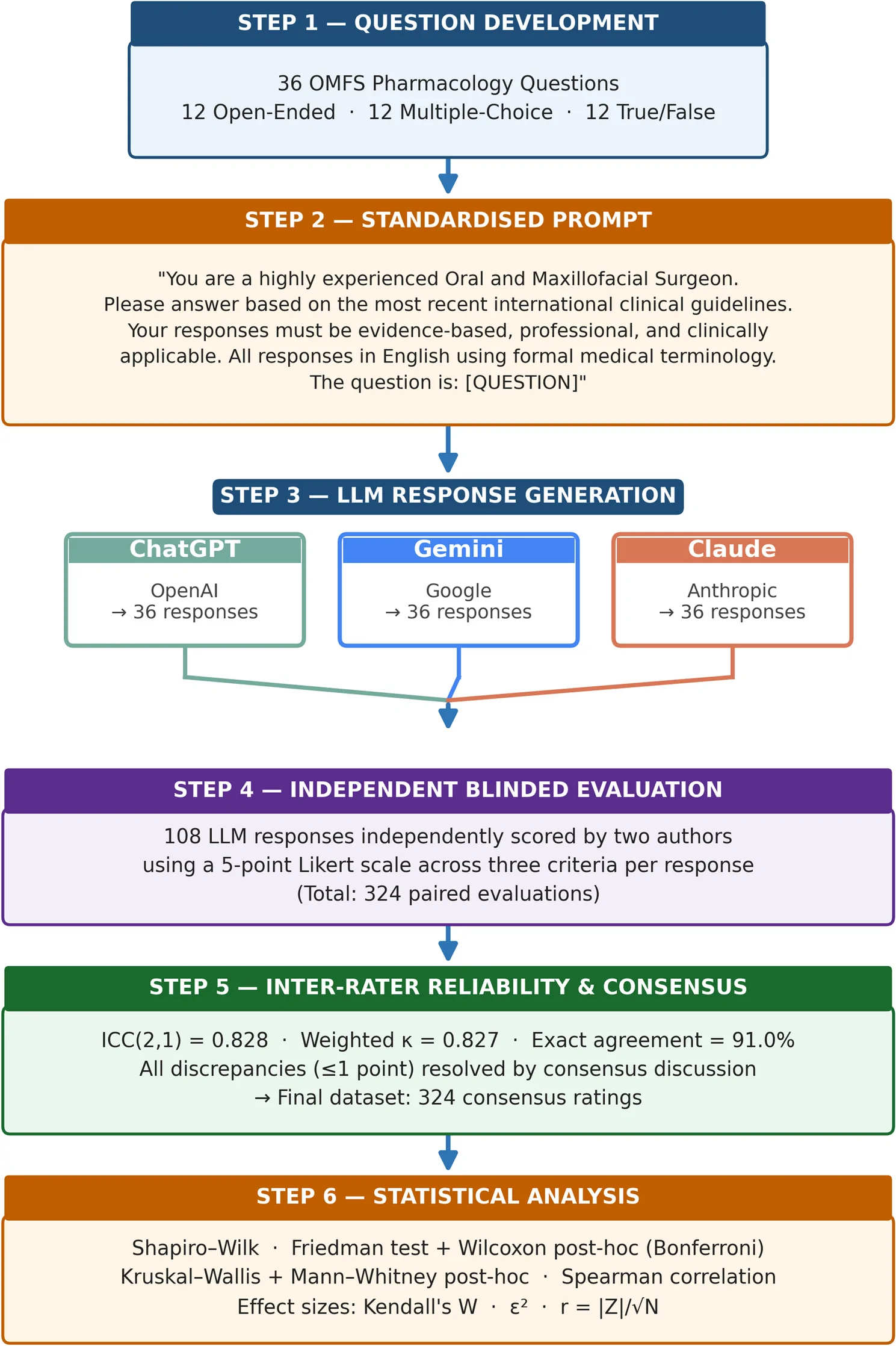
Schematic overview of the study methodology. The workflow comprised six sequential steps: (1) development of 36 OMFS pharmacology questions by an expert panel; (2) standardised role-conditioning prompt engineering; (3) independent response generation by ChatGPT, Gemini, and Claude; (4) blinded evaluation by two authors using a 5-point Likert scale; (5) inter-rater reliability assessment and consensus resolution; and (6) non-parametric statistical analysis. Final dataset: 324 consensus ratings (36 questions × 3 LLMs × 3 criteria)

This study employed a cross-sectional, comparative in silico design to evaluate three publicly accessible LLMs, ChatGPT 5.3 (OpenAI), Gemini 3.1 Pro (Google DeepMind), and Claude 4.6 Sonnet (Anthropic), on pharmacology questions relevant to OMFS. All models were accessed concurrently on April 1, 2026 via their official public web interfaces. The methodological framework was aligned with that of recent comparative LLM-evaluation studies [5–7, 11, 12]. As this study did not involve human participants, patient data, or clinical interventions, formal ethics committee approval was not required; the study was conducted in accordance with the Declaration of Helsinki. A schematic overview of the complete study workflow is presented in Fig. 1.

### Question development and content validation

A structured bank of 36 OMFS pharmacology questions was developed by the two authors prior to any interaction with the LLMs, thereby eliminating the risk of reverse engineering. The thematic scope encompassed five clinical domains: (i) antibiotic prophylaxis and therapeutic regimens; (ii) analgesics and perioperative pain management; (iii) clinically significant drug–drug interactions; (iv) local and general anesthetic pharmacology; and (v) pharmacological considerations in special populations (pregnant, lactating, pediatric, geriatric, and medically compromised patients). Theme selection was guided by AAOMS, ADA, and AHA infective endocarditis prophylaxis guidelines [8], following the approach of Çırakoğlu et al. [11]. The 36 items were equally stratified across three formats (*n* = 12 each): open-ended, multiple-choice (MCQ; five options, one correct), and true/false (T/F). The complete question set and reference answers are provided in Supplementary File 1. The selection of 36 questions mirrored the design of Çırakoğlu et al. [11] to enable direct methodological comparability. This sample size was further selected to provide balanced representation across the five clinical domains (approximately seven items per domain) and three question formats (12 items each), while remaining feasible for two independent raters to evaluate within a single session without evaluator fatigue, and is consistent with prior LLM benchmarking studies in dental and OMFS contexts [5–7, 11].

### Large language models evaluated

Three publicly accessible LLMs were evaluated: ChatGPT 5.3 (OpenAI), Gemini 3.1 Pro (Google DeepMind), and Claude 4.6 Sonnet (Anthropic). These were the most advanced publicly available versions of each model, as offered through their respective consumer web interfaces on the access date (April 1, 2026). All models were queried through their official web interfaces (chatgpt.com, gemini.google.com, and claude.ai, respectively), without API access or third-party wrappers. No custom system instructions, plugins, browsing tools, or retrieval features were enabled; each interface used its default model configuration. All queries were completed within a single calendar day to minimize temporal bias arising from interim model updates.

### Response generation protocol

All 36 questions were submitted by a single trained investigator in a fixed sequence [5, 7, 11]. Before each query, browser cookies, cache, and history were cleared; a dedicated study account was used; a fresh chat window was initiated for every individual question; and memory-retention features were disabled where available [7]. A role-conditioning (persona) prompt was chosen rather than a bare zero-shot query to approximate a realistic use case in which a clinician explicitly frames the model as a domain expert and to standardize the querying context across models. A single standardized prompt was therefore applied uniformly across all 108 query sessions, identically for every model and every question, so that any observed inter-model differences could not be attributed to variation in prompt wording. The prompt, presented immediately before each question, was:

“You are a highly experienced Oral and Maxillofacial Surgeon. Please answer the following questions based on the most recent international clinical guidelines. Your responses must be technically profound, evidence-based, professional, and clinically applicable. All responses must be provided in English using formal medical terminology. The question is: [QUESTION].”

Each question was submitted exactly once without iterative prompting [11, 12]. All responses were anonymized, randomly coded, and stored verbatim for blinded evaluation. A single-session evaluation was used to ensure temporal homogeneity and prevent confounding by intervening model updates, consistent with prior benchmarking studies in dental and OMFS contexts [5, 6, 11]. The implications of this design choice for response variability are addressed in the Limitations section.

### Evaluation framework and scoring rubric

All 108 responses (36 questions × 3 LLMs) were independently and blindly evaluated by two raters, a specialist oral and maxillofacial surgeon (> 3 years of post-specialty clinical experience) and a resident in oral and maxillofacial surgery, following a structured calibration session using ten pilot responses [6, 11]. Each response was rated on a 5-point Likert scale (1 = poor; 5 = excellent) across three pre-defined criteria: Accuracy, Comprehensiveness, and Usefulness for open-ended items; Correctness, Accuracy of Explanation, and Usefulness of Explanation for MCQs; and Correctness, Accuracy of Justification, and Usefulness of Justification for T/F items. Full operational definitions and Likert anchor descriptors are provided in Supplementary File 2. This multi-dimensional framework was adopted in line with the approach of Maia et al. [12], Tam et al. [13], and the Global Quality Scale applied in OMFS evaluations [11]. Blinding was maintained throughout: each rater evaluated all 108 responses independently, without access to the other rater’s scores. Responses were presented in randomised order with the originating LLM concealed, and raters were instructed not to attempt to identify the source platform. The risk of partial deblinding through stylistic recognition (e.g., bullet-point versus prose formatting characteristic of specific platforms) is acknowledged as a limitation.

### Inter-rater reliability and consensus resolution

Inter-rater reliability was assessed on the complete pre-consensus dataset comprising 324 paired ratings (36 questions × 3 LLMs × 3 criteria) using the Intraclass Correlation Coefficient (ICC [1, 2]) under a two-way random-effects, absolute-agreement, single-measurement model, as recommended by Koo and Li [14]. ICC thresholds: <0.50, poor; 0.50–0.74, moderate; 0.75–0.89, good; ≥0.90, excellent. Cohen’s quadratic-weighted kappa (κ_w), unweighted kappa (κ), and Spearman’s ρ were additionally computed to complement the ICC and address ceiling-effect limitations [11, 12].

Discrepancies were resolved through a three-tier procedure: (i) identical scores → automatic consensus; (ii) ± 1-point discrepancy → joint discussion; (iii) ≥ 2-point discrepancy → independent third-rater adjudication (pre-specified per Grillo et al. [6]; not required in practice, as no pair differed by more than one point). The final dataset comprised 324 consensus ratings.

### Statistical analysis

Analyses were performed using IBM SPSS Statistics v29.0 and Python 3.12 (SciPy v1.17.1) at two-tailed α = 0.05. Given significant deviations from normality (Shapiro–Wilk, *p* < 0.05) and pronounced ceiling effects [11, 12], non-parametric methods were used throughout. The Friedman test (with Kendall’s W as effect-size estimator) compared per-question grand totals, section totals, and per-criterion scores across LLMs, followed by Bonferroni-corrected Wilcoxon signed-rank post-hoc tests (corrected α = 0.0167; effect size r = |Z|/√N). Within-model differences across question types were assessed using Kruskal–Wallis H tests (ε² effect size) followed by Bonferroni-corrected Mann–Whitney U pairwise tests. Spearman’s ρ was computed for inter-criterion correlations to examine the convergence among the evaluation criteria. Multivariable or mixed-effects modelling was not applied: the ordinal Likert structure of the data, the limited number of independent items (*n* = 36), and the pronounced ceiling effects observed in two of the three models would not support stable multivariable estimation without violating its distributional assumptions. Publication-quality figures were generated using Matplotlib at 300 dpi.

## Results

### Inter-rater reliability

Across all 324 paired ratings, inter-rater reliability was excellent: ICC(2,1) = 0.828 (95% CI: 0.792–0.861), κ_w = 0.827, unweighted κ = 0.804, and Spearman’s ρ = 0.822 (*p* < 0.001). Exact agreement was 91.0%, with all discrepancies within ± 1 point. Section-level ICCs ranged from 0.693 (MCQ) to 0.879 (open-ended), with T/F yielding 0.848; all exceeded the 0.75 good-reliability threshold [14]. Section-level statistics are summarised in Table 1.

**Table 1:** Inter-rater reliability statistics between the two independent evaluators (pre-consensus)

Section	*n*	ICC(2,1) [95% CI]	Weighted κ	Unweighted κ	Spearman ρ	Exact %
Open-ended	108	0.879 [0.828–0.916]	0.878	0.843	0.878	91.7
Multiple-choice	108	0.693 [0.583–0.781]	0.691	0.691	0.696	88.0
True/false	108	0.848 [0.789–0.896]	0.846	0.846	0.851	93.5
Overall	324	0.828 [0.792–0.861]	0.827	0.804	0.822	91.0

*ICC* Intraclass Correlation Coefficient (two-way random-effects, absolute agreement, single measures), *CI *Confidence interval, κ = Cohen’s kappa (quadratic weights for weighted variant); ρ = Spearman’s rank correlation coefficient. Exact % = rating pairs with identical scores

All Spearman’s ρ *p*-values < 0.001

### Descriptive performance across LLMs

Claude achieved the highest overall consensus score (median = 15.0, IQR = 14.8–15.0; mean = 14.75 ± 0.44), followed by Gemini (median = 14.0, IQR = 13.0–15.0; mean = 14.17 ± 0.88) and ChatGPT (median = 13.0, IQR = 12.0–14.0; mean = 12.86 ± 1.13). Claude achieved ceiling-level median scores of 5 across all nine evaluation criteria (mean = 4.92 ± 0.29 throughout). ChatGPT exhibited the greatest criterion-level variability, with the lowest mean scores in the open-ended section, particularly Comprehensiveness (3.92 ± 0.51) and Usefulness (3.83 ± 0.39). Detailed criterion-level statistics are presented in Table 2.

**Table 2:** Consensus scores per criterion for each LLM — median (IQR) and mean ± SD (*n* = 12 per cell)

Section / Criterion	ChatGPT Median (IQR)	ChatGPT Mean ± SD	Gemini Median (IQR)	Gemini Mean ± SD	Claude Median (IQR)	Claude Mean ± SD
Open-Ended						
Accuracy	4 (4–4)	4.08 ± 0.51	5 (5–5)	4.92 ± 0.29	5 (5–5)	4.92 ± 0.29
Comprehensiveness	4 (4–4)	3.92 ± 0.51	5 (5–5)	4.75 ± 0.45	5 (5–5)	4.92 ± 0.29
Usefulness	4 (4–4)	3.83 ± 0.39	4 (4–5)	4.50 ± 0.52	5 (5–5)	4.92 ± 0.29
Multiple-Choice						
Correctness	5 (5–5)	4.92 ± 0.29	5 (5–5)	5.00 ± 0.00	5 (5–5)	4.92 ± 0.29
Accuracy of Explanation	5 (4–5)	4.58 ± 0.51	5 (5–5)	4.75 ± 0.45	5 (5–5)	4.92 ± 0.29
Usefulness of Explanation	4 (4–4)	4.00 ± 0.00	5 (4–5)	4.58 ± 0.51	5 (5–5)	4.92 ± 0.29
True/False						
Correctness	5 (5–5)	4.92 ± 0.29	5 (5–5)	4.92 ± 0.29	5 (5–5)	4.92 ± 0.29
Accuracy of Justification	4 (4–5)	4.33 ± 0.49	5 (4–5)	4.67 ± 0.49	5 (5–5)	4.92 ± 0.29
Usefulness of Justification	4 (4–4)	4.00 ± 0.00	4 (4–5)	4.42 ± 0.51	5 (5–5)	4.92 ± 0.29

*IQR* Interquartile range (Q1–Q3), *SD* Standard deviation

Each criterion scored 1 (poor) to 5 (excellent)

### Between-model comparisons (Friedman Test)

The Friedman test demonstrated highly significant inter-model differences (χ²(2) = 47.91, *p* < 0.001, Kendall’s W = 0.665, large effect). Bonferroni-corrected Wilcoxon post-hoc tests confirmed all three pairwise comparisons: Claude vs. ChatGPT (*p* < 0.001, *r* = 0.85), Gemini vs. ChatGPT (*p* < 0.001, *r* = 0.74), and Claude vs. Gemini (p_Bonf = 0.006, *r* = 0.52). Section-level analyses revealed the largest differences in the open-ended section (W = 0.775), where ChatGPT performed significantly below both Gemini (p_Bonf = 0.003) and Claude (p_Bonf = 0.002), while Gemini and Claude did not differ significantly in any of the three sections (open-ended: p_Bonf = 0.352; MCQ: 0.094; T/F: 0.094). Section-specific results are summarised in Table 3.

**Table 3:** Friedman test results with post-hoc Wilcoxon signed-rank pairwise comparisons

Section	χ²(df = 2)	*p* (Friedman)	Kendall W	ChatGPT vs. Gemini p_Bonf, *r*	ChatGPT vs. Claude p_Bonf, *r*	Gemini vs. Claude p_Bonf, *r*
Open-ended	18.59	< 0.001	0.775	0.003, *r* = 0.95	0.002, *r* = 1.00†	0.352, *r* = 0.45
Multiple-choice	14.89	< 0.001	0.620	0.094, *r* = 0.62	0.003, *r* = 0.95	0.750, *r* = 0.33
True/false	15.17	< 0.001	0.632	0.094, *r* = 0.62	0.006, *r* = 0.89	0.094, *r* = 0.62
Overall (36 items)	47.91	< 0.001	0.665	< 0.001, *r* = 0.74	< 0.001, *r* = 0.85	0.006, *r* = 0.52

χ² = Friedman chi-square; Kendall’s W interpreted as effect size (small ≤ 0.20; moderate 0.20–0.40; large > 0.40). p_Bonf = Bonferroni-corrected p (corrected α = 0.0167). r = Wilcoxon effect size (small ≈ 0.10; medium ≈ 0.30; large ≈ 0.50). *n* = 12 per section; *n* = 36 for overall

†Effect size r capped at the theoretical maximum of 1.00; the computed value (|Z|/√N) approached this ceiling owing to the maximal rank-sum separation between the two paired score distributions

### Within-model comparisons across question types (Kruskal-Wallis)

ChatGPT demonstrated a statistically significant and large performance difference across question types (H(2) = 17.04, *p* < 0.001, ε² = 0.456), with significantly lower scores on open-ended items (mean = 11.83) compared to MCQ (13.50) and T/F (13.25) (p_Bonf < 0.05 for both comparisons). In contrast, Gemini (H(2) = 0.97, *p* = 0.616) and Claude (H(2) = 0.00, *p* > 0.999) demonstrated complete format-independence. Results are presented in Table 4.

**Table 4:** Kruskal-Wallis H test results comparing each LLM’s performance across question types

LLM	H (df = 2)	*p*	ε²	Open-Ended Median (Mean)	MCQ Median (Mean)	True/False Median (Mean)
ChatGPT	17.04	< 0.001	0.456	12.0 (11.83)	14.0 (13.50)	13.0 (13.25)
Gemini	0.97	0.616	0.000	14.5 (14.17)	15.0 (14.33)	14.0 (14.00)
Claude	0.00	> 0.999	0.000	15.0 (14.75)	15.0 (14.75)	15.0 (14.75)

ChatGPT showed a highly significant, large effect indicating differential format-dependent performance; Gemini and Claude exhibited stable performance independent of question format

H = Kruskal-Wallis statistic; ε² = epsilon-squared effect size. *n* = 12 per section

### Inter-criterion correlations

For ChatGPT, all three pairwise inter-criterion Spearman correlations were statistically significant and positive (C1 vs. C2: ρ = 0.465, *p* = 0.004; C1 vs. C3: ρ = 0.412, *p* = 0.013; C2 vs. C3: ρ = 0.463, *p* = 0.004), indicating convergence among the three criteria of the multi-criteria framework. For Gemini and Claude, inter-criterion correlations were largely non-significant, a direct mathematical consequence of severe ceiling effects (Gemini Criterion 1: 34/36 scores = 5; Claude: only three non-maximum scores per criterion at non-overlapping question positions). When inter-item variance is this severely restricted, rank-based correlation cannot detect associations regardless of underlying existence [5]. Convergence among the criteria could therefore be meaningfully estimated only in the ChatGPT data, in which sufficient score variability was present. Results are presented in Table 5.

**Table 5:** Spearman’s rank correlations between evaluation criteria within each LLM’s consensus dataset (*n* = 36)

LLM	Criterion 1 vs. 2	Criterion 1 vs. 3	Criterion 2 vs. 3
ChatGPT	ρ = 0.465, *p* = 0.004*	ρ = 0.412, *p* = 0.013*	ρ = 0.463, *p* = 0.004*
Gemini	ρ = -0.150, *p* = 0.381	ρ = 0.000, *p* > 0.999	ρ = 0.620, *p* < 0.001*
Claude	ρ = -0.091, *p* = 0.598	ρ = -0.091, *p* = 0.598	ρ = -0.091, *p* = 0.598

Non-significant results for Gemini and Claude are attributable to a pronounced ceiling effect (near-constant scores), which mathematically precludes the detection of rank-based associations; these should not be interpreted as evidence against the internal coherence of the framework

C1 = Accuracy/Correctness; C2 = Comprehensiveness/Accuracy of Explanation or Justification; C3 = Usefulness

* *p* < 0.05

### Visual summary of findings

**Fig. 2:**
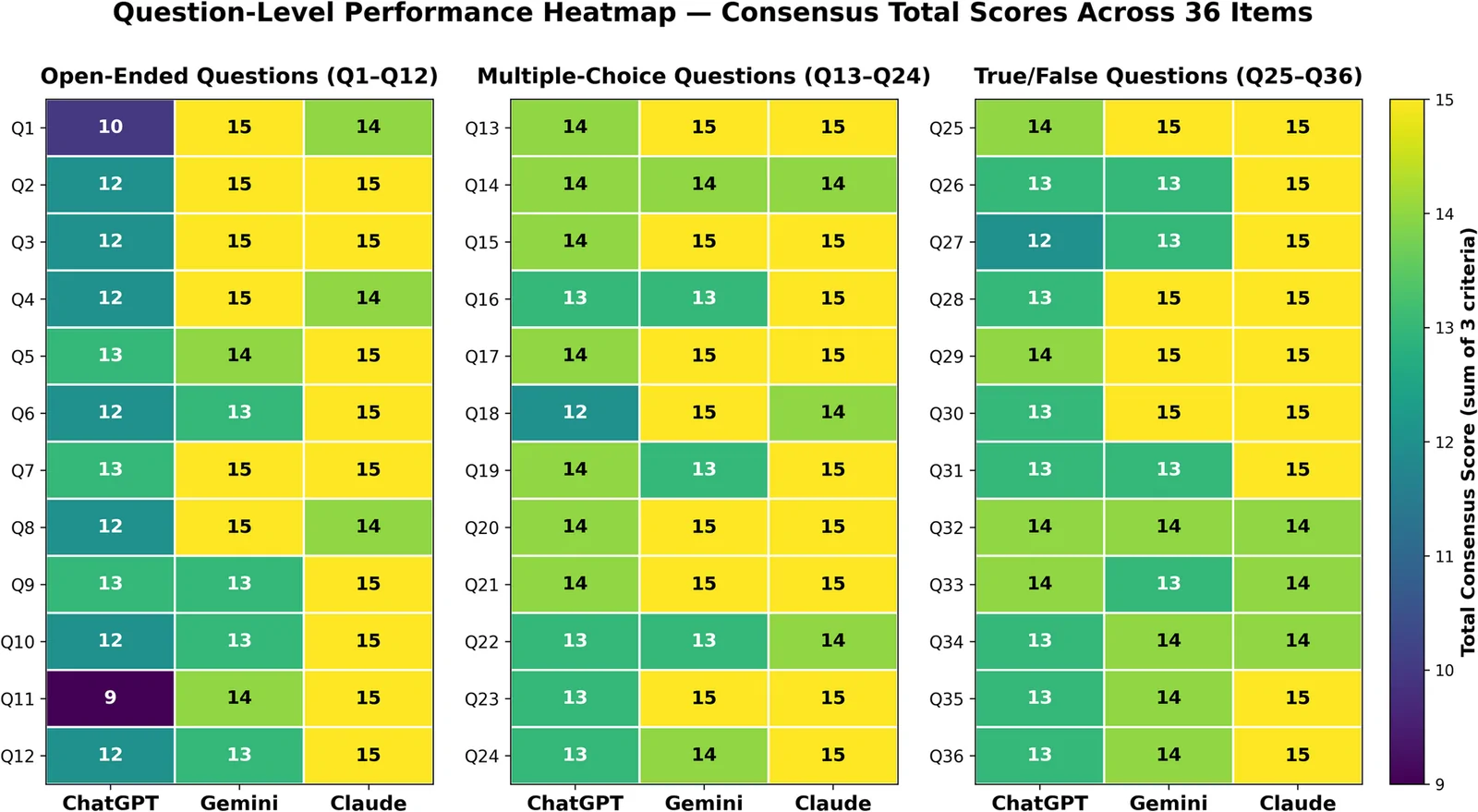
Question-level performance heatmap displaying consensus total scores (range 3–15; sum of three criteria) for all 36 items across the three LLMs. Warmer colours indicate lower performance; cooler colours indicate higher performance

**Fig. 3:**
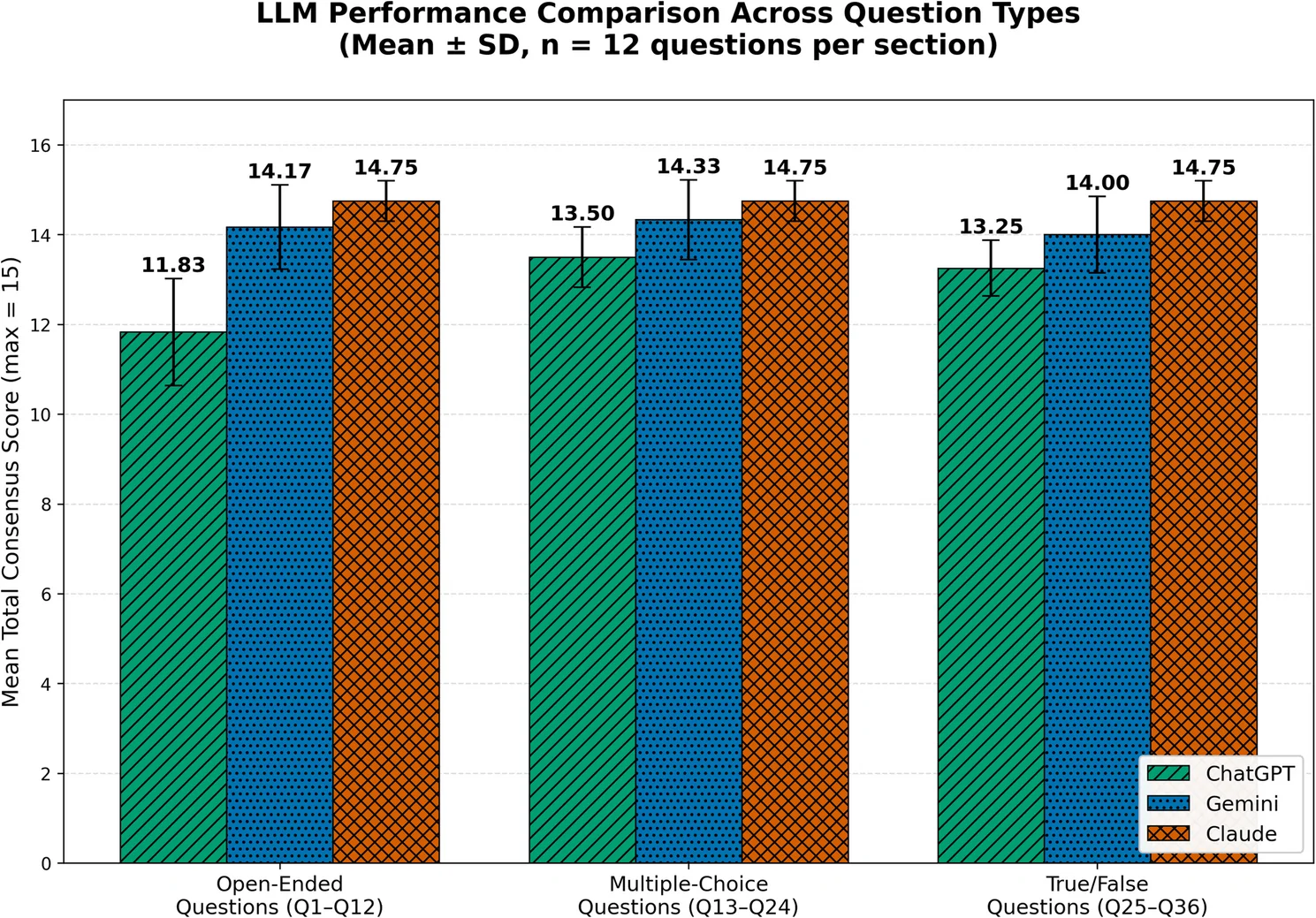
Mean total consensus score per LLM (± SD) across the three question types. The performance hierarchy (Claude ≥ Gemini > ChatGPT) is consistent across all sections, with the largest inter-model gap in the open-ended section

Figure 2 (heatmap) illustrates ChatGPT’s heterogeneous open-ended performance, notably Q1 (10/15) and Q11 (9/15), contrasting with Claude’s uniformly high scores across all 36 items. Figure 3 confirms a consistent hierarchy of Claude ≥ Gemini > ChatGPT across all formats, with the largest inter-model gap in the open-ended section.

## Discussion

The present study provides, to our knowledge, the first blinded, multi-criteria comparative evaluation of ChatGPT 5.3, Gemini 3.1 Pro, and Claude 4.6 Sonnet in OMFS pharmacology across five clinical domains and three question formats. The principal finding, a robust performance hierarchy of Claude > Gemini > ChatGPT (χ²(2) = 47.91, *p* < 0.001, W = 0.665, large effect), with all three pairwise post-hoc comparisons significant after Bonferroni correction, extends the growing body of evidence on differential LLM capability in dental and surgical disciplines. Importantly, section-level analyses revealed that this hierarchy was driven predominantly by ChatGPT’s underperformance: Gemini and Claude did not differ significantly in any format section, indicating clinical equivalence across all structured pharmacological formats. These findings are directly motivated by Quah et al.’s demonstration that pharmacology represents the lowest-performing OMFS subcategory (45.9%) [5], establishing the targeted evidence gap that present benchmark addresses.

The superior performance of Claude 4.6 Sonnet contrasts markedly with earlier investigations employing older model generations. Fujimoto et al., evaluating ChatGPT-4, Claude 3 Opus, and Gemini 1.0 on dental anesthesiology board examinations, reported accuracy rates of 51.2%, 47.4%, and 30.3%, respectively, a hierarchy in which Gemini performed markedly lowest [15]. In a direct OMFS comparison, Grillo et al. evaluated six LLMs on OMFS-relevant clinical prompts and demonstrated substantial task-dependent variability, with no single model achieving consistent superiority across all evaluated domains [6]. Sato et al. tracked 18 AI models across 2024 and observed leadership shifting sequentially from ChatGPT to Claude to Gemini across successive versions [16]; Nguyen et al. showed Claude 3.5 Sonnet achieving the highest overall accuracy (85%) among six LLMs in antibiotic prescribing vignettes across four countries [17]. Wójcik et al. similarly demonstrated that Claude was the only model to consistently pass the Polish dental licensing examination threshold across both prompt languages [18]. Crucially, De Vito et al. specifically noted that older Claude and Gemini versions yielded the lowest accuracy among 14 LLMs in antibiotic prescribing scenarios [10], directly contextualizing the substantially improved performance of both Claude 4.6 Sonnet and Gemini 3.1 Pro observed in the present study. Collectively, these data support the interpretation that the current hierarchy reflects genuine, version-specific capability gains rather than a fixed architectural invariant, and underscore the imperative for contemporaneous benchmarking as these platforms continue to evolve.

The most clinically informative within-model finding was ChatGPT’s pronounced format-dependent decline in performance on open-ended questions (H(2) = 17.04, *p* < 0.001, ε² = 0.456), a pattern entirely absent in Gemini and Claude. ChatGPT’s mean open-ended score (11.83) fell ~ 13% below its own MCQ performance (13.50), with Comprehensiveness and Usefulness criteria registering the lowest values across the entire dataset. The heatmap reveals that ChatGPT’s lowest individual scores occurred on items requiring multi-step guideline synthesis with complicating clinical variables (Q1: 10/15; Q11: 9/15). These items demanded narrative integration of multi-domain pharmacological knowledge and contextualized recommendations, precisely the competencies that prior work has identified as differentially challenging. Liu et al. demonstrated a consistent decline from factual recall to clinical case-based reasoning across all LLMs evaluated on OMFS content [7]. Ji et al. showed that standard direct querying yielded significantly inferior open-ended scores compared to Chain-of-Thought prompting in OMFS dental licensing [19], suggesting ChatGPT’s vulnerability is sensitive to prompt architecture. The role-conditioning prompt employed here likely elevated performance across all models relative to zero-shot designs such as Fujimoto et al.’s [15], limiting direct numeric comparability; Rewthamrongsris et al. confirmed that preprompting is a significant performance modifier across 10 LLMs evaluated on OMFS-relevant infective endocarditis prophylaxis [9]. The systematic review of Ronsivalle et al. concluded that LLM accuracy declines notably in complex or non-standardized cases relative to straightforward scenarios [20]. These observations suggest ChatGPT’s open-ended vulnerability reflects a limitation in spontaneous integrative clinical reasoning rather than a global pharmacological knowledge deficit. It is important to note, however, that the open-ended items were not merely a different presentation format but also carried the highest intrinsic content complexity, requiring multi-step synthesis and contextual judgement; the observed decline therefore most likely reflects a confounded combination of question format and underlying task difficulty rather than a pure format effect, as discussed further in the Limitations. This interpretation is consistent with recent evidence that LLM performance in oral and maxillofacial disorders is markedly weaker in case-based, history-taking, and open-ended diagnostic-reasoning tasks than in structured, knowledge-based questions, underscoring the need for greater caution when these models are applied to complex, open-ended clinical scenarios [21].

The consistently high MCQ and T/F Correctness scores across all models (mean ≥ 4.92 in MCQ Correctness) align with prior evidence that current-generation LLMs possess broad declarative knowledge of pharmacology. Nguyen et al. found no significant inter-model differences in the pharmacology subgroup of dental MCQs (*p* = 0.934), attributing this to the generalized knowledge bases of contemporary LLMs [17]. The performance pattern therefore reflects a domain-specific asymmetry: structured knowledge retrieval is broadly accessible, whereas integrative open-ended reasoning, encompassing DDI management, dose adjustment for special populations, and guideline-adherent multi-drug prescribing, remains differentially challenging. This aligns with Antonie et al.’s observation that AI chatbots demonstrate satisfactory accuracy in guideline-based antibiotic recommendations but struggle with the contextual nuances required for complex prescribing in medically compromised patients [2]. Sicard et al., evaluating ChatGPT, Claude, and Gemini on real-world DDI cases from the French National Pharmacovigilance Database, demonstrated high DDI detection sensitivity (95–99%) but comprehensive characterisation of interaction type, mechanism, and management correct in only 66–68% for ChatGPT and Claude and 33% for Gemini, with accuracy declining as interaction complexity increased [22], a gradient directly paralleling ChatGPT’s format-dependent decline observed here. These findings are particularly salient in OMFS, where DDI management in patients receiving anticoagulants, antihypertensives, or immunosuppressants alongside perioperative analgesics and antibiotics represents one of the highest-risk prescribing contexts in ambulatory surgical care.

The pre-specified inter-criterion correlation analysis indicated that the three scoring dimensions converged as intended, supporting the internal coherence of the multi-criteria evaluation framework. For ChatGPT, all three inter-criterion correlations were significant and positive, demonstrating meaningful co-variation of Accuracy, Comprehensiveness, and Usefulness across questions. Non-significant correlations for Gemini and Claude reflect their severe ceiling effects rather than framework incoherence — consistent with the well-documented property that rank-based correlation cannot detect associations in near-constant data [12]. The inter-rater reliability achieved (ICC = 0.828, κ_w = 0.827, exact agreement 91.0%) represents one of the highest values reported in the LLM-evaluation literature for OMFS, against which Çırakoğlu et al. reported ICCs of 0.314 and 0.001 with a single-rater design [11]. The MCQ section yielded the lowest ICC (0.693), consistent with greater subjectivity in evaluating explanatory responses; the open-ended section paradoxically achieved the highest (0.879), likely reflecting the structured calibration protocol and pre-specified comprehensive reference answers. The multi-dimensional framework aligns with the approaches of Maia et al. and Tam et al., who demonstrated that single-metric accuracy alone is insufficient to capture clinically relevant dimensions of LLM response quality [12, 13]. It is important, however, to interpret ceiling-level performance with appropriate caution: uniformly maximal scores on a structured benchmark do not necessarily imply clinical equivalence in real-world settings, where response nuance under ambiguity, sensitivity to atypical clinical presentations, and judgment in DDI management at the limits of current evidence may reveal performance differences not captured by the present framework.

This study has several limitations. First, performance was assessed at a single time point; given the pace of model evolution demonstrated by Sato et al. [16] and the performance inversions across model generations [10, 15, 18], present rankings may not persist with model updates. Furthermore, each query was administered only once per LLM; given the inherent stochasticity of large language model outputs (particularly for open-ended responses), results from a single administration may not fully capture the variability in responses and would benefit from confirmation through multi-run replication in future work. Second, the reference answers used as evaluation standards were developed and internally reviewed by the two study authors; independent external validation by a third specialist blinded to the LLM outputs was not performed, which may introduce a degree of criterion bias. Third, expert-evaluator scoring, a pragmatic necessity for narrative responses, introduces subjectivity despite robust inter-rater procedures. Fourth, only three commercial LLMs via standard interfaces were evaluated; open-source, fine-tuned, and Retrieval-Augmented Generation (RAG)-enhanced implementations were not assessed. Rewthamrongsris et al. demonstrated that RAG augmentation does not uniformly improve performance and is highly model-dependent [9], suggesting future OMFS pharmacology benchmarking should systematically include RAG conditions. Fifth, the standardized English prompt may differentially favor models with stronger English corpora, as demonstrated by Wójcik et al. [18]. Relatedly, a single fixed role-conditioning prompt was used for all queries; because prompt design is itself a recognised performance modifier, the absolute scores and the magnitude of inter-model differences reported here may be specific to this prompt, and alternative prompting strategies (e.g., zero-shot, chain-of-thought, or iterative prompting) could yield different results. Sixth, in silico benchmark performance may not generalize to real-world clinical contexts involving incomplete information, ambiguity, and time pressure. Seventh, question format and intrinsic task difficulty were not fully separable in the present design: the open-ended items inherently demanded more multi-step, integrative clinical reasoning than the multiple-choice and true/false items. ChatGPT’s open-ended decline should therefore be interpreted as a combined format-and-complexity effect rather than a pure effect of question format; disentangling these factors would require future designs that hold content difficulty constant across formats (e.g., matched-difficulty item sets presented in multiple formats). Finally, although raters were blinded to the originating LLM and presented with randomly coded responses, partial deblinding via stylistic features characteristic of each platform cannot be entirely ruled out as a residual source of bias.

The clinical implications must be situated within an incompletely characterized risk landscape. The superior, format-independent performance of Claude 4.6 Sonnet suggests it may currently represent the most reliable of the three platforms for OMFS pharmacological information support, while ChatGPT warrants caution in open-ended clinical reasoning. However, these recommendations are explicitly tentative and version-specific. As Bélisle-Pipon has argued, LLM outputs represent statistically plausible text generation rather than evidence-based clinical reasoning, a distinction particularly consequential in pharmacology, where confident incorrect recommendations may be indistinguishable from accurate ones to non-specialist users [23]. AlGain et al.’s systematic review of 23 AI antibiotic prescribing studies concluded that LLMs currently lack reliability for complex clinical applications and that specialist oversight remains indispensable [24]. Giacobbe et al. emphasized that no LLM tool is currently approved by regulatory authorities for clinical pharmacological decision-making [25]. Future research should prioritize prospective evaluation in real-world OMFS workflows, head-to-head comparison with specialist benchmark answers, and systematic investigation of RAG-enhanced, guideline-grounded implementations. Longitudinal version tracking remains necessary to translate single benchmarking studies into durable clinical guidance.

## Conclusion

Within a blinded, multi-criteria framework with strong inter-rater reliability (ICC = 0.828), Claude 4.6 Sonnet and Gemini 3.1 Pro both achieved near-maximal scores across the structured OMFS question formats of this benchmark, whereas ChatGPT 5.3 demonstrated a significant decline on open-ended items requiring integrative clinical reasoning. The version-specific reversal of inter-model rankings relative to earlier benchmarks confirms that LLM capability in OMFS pharmacology is rapidly evolving and platform-dependent. Until prospective real-world validation, regulatory approval, and version-stable performance data are available, current LLMs should be regarded as adjunctive informational tools requiring expert clinical verification, particularly for high-risk pharmacological decisions involving DDI management, special populations, and complex perioperative prescribing in oral and maxillofacial surgery. Moreover, safe clinical integration of LLMs depends not only on response accuracy but also on data security, anonymization, and adherence to the ethical and legal obligations governing patient information, domains in which dental practitioners’ awareness remains limited [26]. Accuracy benchmarks such as the present study should therefore be complemented by attention to privacy and ethical safeguards before LLMs are incorporated into routine OMFS workflows.

## Supplementary Information

Supplementary Material 1.

Supplementary Material 2.

## Data Availability

The complete question bank and reference answers supporting the findings of this study are available as Supplementary Files 1 and 2 accompanying this manuscript. Additional data are available from the corresponding author upon reasonable request.

## Abbreviations

AAOMS: American Association of Oral and Maxillofacial Surgeons
ADA: American Dental Association
AHA: American Heart Association
AI: Artificial intelligence
API: Application programming interface
CI: Confidence interval
CoT: Chain-of-Thought
DDI: Drug–drug interaction
ICC: Intraclass Correlation Coefficient
LLM: Large language model
MCQ: Multiple-choice question
NLP: Natural language processing
OMFS: Oral and maxillofacial surgery
RAG: Retrieval-Augmented Generation
SPSS: Statistical Package for the Social Sciences
T/F: True/false
