# Supplementary material for: Do large language models differ in their pharmacology-related response quality for oral and maxillofacial surgery? a blinded expert benchmark study
Source: BMC Oral Health. 2026 Jul 4;26:1808. doi: 10.1186/s12903-026-09182-w (PMC13602642; doi:10.1186/s12903-026-09182-w)
Supplement: Supplementary file 1 [file 12903_2026_9182_MOESM1_ESM.docx]

| **Supplementary Table 1:** Questions and answers provided by the three LLM platforms | | | |
| --- | --- | --- | --- |
|  | **Response** | | |
|  | **Chatgpt** | **Gemini** | **Claude** |
| 1. **Explain the indications for prophylactic antibiotic use following asymptomatic impacted mandibular third molar surgery according to current international guidelines?** | Evidence-based position from current international guidelines  Contemporary international guidance (including the American Dental Association [ADA], American Heart Association [AHA], and broader antimicrobial stewardship frameworks) has fundamentally shifted toward restricting antibiotic prophylaxis in oral surgery. The key principle is:  Routine prophylactic antibiotic use is NOT indicated in healthy patients undergoing asymptomatic impacted mandibular third molar surgery.  This reflects a risk–benefit reassessment driven by high-quality evidence and concerns regarding antimicrobial resistance.    1. Routine use after third molar surgery: Not recommended  For asymptomatic, non-infected impacted mandibular third molars in otherwise healthy individuals:  • Antibiotic prophylaxis is not routinely indicated, either preoperatively or postoperatively.  • The rationale:  o Low incidence of postoperative infection (typically <5–10% in standard cases).  o Lack of consistent evidence demonstrating clinically meaningful benefit.  o Risk of adverse drug reactions and antimicrobial resistance outweighs benefits.  Additionally, authoritative reviews emphasize:  • “Antimicrobial prophylaxis is not routinely indicated for healthy patients undergoing dental or oral surgery procedures.”  This applies directly to uncomplicated third molar surgery.    2. Indications for prophylactic antibiotics: Patient-related (systemic) risk factors  Guidelines consistently emphasize that indications are patient-specific—not procedure-specific.  A. High-risk cardiac conditions (AHA/ADA consensus)  Prophylaxis is indicated only in patients at highest risk of infective endocarditis, including:  • Prosthetic cardiac valves or valve repair material  • Previous infective endocarditis  • Certain congenital heart diseases  • Cardiac transplant recipients with valvulopathy  In such patients, any procedure involving mucosal incision (including third molar surgery) warrants prophylaxis.    B. Severe immunocompromise or systemic vulnerability  Although not uniformly codified across all guidelines, widely accepted indications include:  • Uncontrolled diabetes mellitus  • Immunosuppressive therapy (e.g., chemotherapy, high-dose corticosteroids)  • Advanced HIV infection  • Hematologic malignancy  • Organ transplantation  In these patients, antibiotics may be considered due to impaired host defense and increased infection risk.    C. Selected complex medical scenarios  Prophylaxis may be considered (case-by-case):  • Recent prosthetic joint complications (not routine; requires specialist consultation)  • History of radiotherapy to the jaws (controversial; more relevant for osteoradionecrosis prevention than infection)  • Extensive reconstructive surgery or grafting procedures    3. Procedure-related indications (limited and selective)  Even in otherwise healthy patients, prophylaxis may be considered when surgical morbidity is expected to be high:  • Prolonged, traumatic surgery  • Extensive bone removal or multiple impactions  • Presence of intraoperative contamination  • Concomitant infection (i.e., not asymptomatic cases)  However, this remains controversial, and many guidelines still favor no routine use even in these scenarios unless additional risk factors are present.    4. Postoperative antibiotics: Also not routinely indicated  Modern evidence-based protocols distinguish between:  • Prophylaxis (preoperative single dose)  • Postoperative antibiotic therapy (extended courses)  For asymptomatic third molar surgery:  • Routine postoperative antibiotics are not recommended  • Management should prioritize:  o Atraumatic technique  o Adequate irrigation  o Proper flap design and closure  o Analgesia and local measures    5. Core principle: Antibiotic stewardship  Current international consensus emphasizes:  • Antibiotics should be prescribed only when clearly indicated  • Overprescription leads to:  o Antimicrobial resistance  o Clostridioides difficile infection  o Drug-related adverse events  Thus:  “Antibiotic prophylaxis should be reserved for patients at the greatest risk of post-treatment complications.”    6. Practical clinical summary (OMFS perspective)  For asymptomatic impacted mandibular third molar surgery:  Indicated:  • High-risk cardiac conditions (AHA criteria)  • Severe immunocompromise  • Selected complex/high-risk surgical or systemic scenarios  Not indicated:  • Healthy patients  • Routine third molar extractions  • Standard postoperative use    Bottom line  From a contemporary OMFS standpoint aligned with international guidelines:  • Routine prophylactic antibiotics after asymptomatic third molar surgery are unjustified.  • Indications are restricted to well-defined high-risk systemic conditions—not the surgical procedure itself.  • Clinical decision-making should prioritize risk stratification and antibiotic stewardship, rather than habitual prescribing.    If you want, I can extend this into a guideline-compliant prescription protocol (drug, dose, timing) or compare AAOMS vs NICE vs European recommendations, which show some nuanced differences. | Based on the most recent parameters of care established by the American Association of Oral and Maxillofacial Surgeons (AAOMS), Cochrane systematic reviews, and global antimicrobial stewardship protocols, the paradigm regarding antibiotic prophylaxis in dentoalveolar surgery has definitively shifted.  The unequivocal consensus is that the routine prescription of postoperative systemic antibiotics following the surgical extraction of asymptomatic, disease-free impacted mandibular third molars in immunocompetent patients is strictly contraindicated.  Here is the evidence-based rationale and a detailed breakdown of the specific clinical scenarios where prophylactic antibiotic use is indicated.  1. The Evidence-Based Rationale Against Routine Use  In healthy patients, the baseline rate of surgical site infection (SSI) following third molar surgery is remarkably low, typically ranging from 1% to 5%.  • Statistical Inefficacy: Extensive Cochrane meta-analyses demonstrate that the Number Needed to Treat (NNT) with systemic antibiotics to prevent a single, localized SSI or case of alveolar osteitis is prohibitively high.  • Iatrogenic Risk: The marginal benefit of routine prophylaxis is vastly outweighed by the exponential risks of adverse drug events (e.g., severe hypersensitivity reactions, gastrointestinal toxicity), the precipitation of Clostridioides difficile-associated pseudomembranous colitis, and the dangerous promotion of multi-drug-resistant organisms within the oral microbiome.  2. Validated Clinical Indications for Prophylaxis  The administration of prophylactic antibiotics is reserved strictly for specific systemic host factors or profound surgical complexities. When indicated, these fall into the following categories:  A. Immunocompromised Host States Patients with systemic conditions that attenuate the innate immune system's ability to sterilize the transient bacteremia induced by surgical trauma require prophylaxis. Indications include:  • Poorly Controlled Diabetes Mellitus: Specifically, patients exhibiting an HbA1c > 8% or fasting blood glucose levels indicative of significant metabolic dysregulation, leading to impaired neutrophil chemotaxis and delayed granulation.  • Systemic Immunosuppression: Patients currently undergoing active chemotherapy, high-dose systemic corticosteroid therapy, or utilizing Disease-Modifying Antirheumatic Drugs (DMARDs) and biologics (e.g., methotrexate, anti-TNF agents) for autoimmune conditions.  • Severe Neutropenia or Leukopenia: Typically defined as an Absolute Neutrophil Count (ANC) of < 2,000 cells/µL.  • Advanced Organ Failure: Patients with End-Stage Renal Disease (ESRD) on hemodialysis or those with advanced hepatic cirrhosis (Child-Pugh Class B or C) with associated reticuloendothelial dysfunction.  B. Prevention of Distant Metastatic Infection As per the most recent American Heart Association (AHA) and European Society of Cardiology (ESC) guidelines, antibiotic prophylaxis is mandatory (irrespective of the tooth's asymptomatic status) to prevent infective endocarditis in highest-risk cohorts. This includes patients with:  • Prosthetic cardiac valves or prosthetic material used for cardiac valve repair.  • A prior history of infective endocarditis.  • Specific, unrepaired cyanotic congenital heart diseases, or repaired defects with residual shunts.  C. Extreme Surgical Complexity (Relative Indication) While routine impactions do not require antibiotics, highly complex surgical interventions may warrant prophylaxis. This includes:  • Extensive ostectomy and tooth sectioning leading to severely prolonged operative times (e.g., > 2 hours).  • Procedures requiring substantial raising of mucoperiosteal flaps adjacent to anatomically compromising spaces (e.g., deep lingual space involvement or extensive trauma to the retromolar trigone).  3. The Pharmacokinetic Protocol: Preoperative vs. Postoperative  If an indication for prophylaxis is met in a case devoid of pre-existing acute suppuration, the standard of care dictates the administration of a single preoperative loading dose rather than a prolonged postoperative course.  • The Protocol: Administration of a bactericidal agent (e.g., Amoxicillin 2g PO, or IV Cefazolin 2g) 30 to 60 minutes prior to surgical incision.  • Clinical Rationale: This pharmacokinetic timing ensures that peak plasma and target tissue concentrations are achieved at the exact moment the mucosal barrier is breached, preventing initial bacterial adhesion and colonization of the surgical hematoma.  • Postoperative Courses: Extending antibiotic therapy for 5 to 7 days postoperatively in clean-contaminated elective surgery provides no statistically significant reduction in SSI rates and is considered clinically obsolete unless the patient is severely immunocompromised (e.g., ANC < 1000) or active purulence was encountered intraoperatively. | Prophylactic Antibiotic Use Following Asymptomatic Impacted Mandibular Third Molar Surgery  Preamble: Current Guideline Landscape  The indications for antibiotic prophylaxis in third molar surgery have undergone substantial revision over the past decade, driven by two converging imperatives: the global antimicrobial resistance (AMR) crisis and a growing body of high-quality evidence — including systematic reviews and Cochrane analyses — demonstrating that routine antibiotic prophylaxis in healthy, immunocompetent patients undergoing elective third molar extraction is not supported by contemporary evidence.  The primary authoritative sources informing current practice include:  • Scottish Dental Clinical Effectiveness Programme (SDCEP) – Drug Prescribing for Dentistry (3rd ed., 2016, updated guidance 2023)  • European Association for Cranio-Maxillo-Facial Surgery (EACMFS) clinical recommendations  • American Association of Oral and Maxillofacial Surgeons (AAOMS) position papers  • SIGN (Scottish Intercollegiate Guidelines Network) and NICE (UK) antimicrobial stewardship guidelines  • Cochrane Systematic Reviews (Lodi et al., 2021 — antibiotics to prevent complications following tooth extractions)    I. General Principle: Antimicrobial Stewardship First  Current international consensus unequivocally establishes that antibiotic prophylaxis should NOT be prescribed routinely for asymptomatic impacted third molar surgery in systemically healthy patients. The prevailing rationale is anchored in three pillars:  1. Lack of proven clinical benefit in reducing postoperative infection rates when weighed against the baseline complication risk in ASA Class I–II patients  2. Risk of adverse drug reactions (gastrointestinal disturbance, hypersensitivity, Clostridioides difficile colitis)  3. Contribution to antimicrobial resistance at both individual and population levels  The Lodi et al. Cochrane Review (2021), analyzing data from over 2,000 patients, concluded that while prophylactic amoxicillin modestly reduces the risk of alveolar osteitis and postoperative infection, the absolute risk reduction does not justify routine universal administration in healthy individuals, and the number needed to treat (NNT) remains high.    II. Accepted Indications for Antibiotic Prophylaxis  A. Patient-Related (Host) Factors  Antibiotic prophylaxis is indicated when the patient's systemic status impairs normal immune response or increases the risk of local or systemic infectious sequelae:  Clinical Condition Guideline Basis  Immunocompromised states (HIV/AIDS with CD4 <200 cells/µL, active chemotherapy, immunosuppressive therapy post-transplant) AAOMS, SDCEP  Poorly controlled Diabetes Mellitus (HbA1c >9% / >75 mmol/mol) SDCEP, SIGN  Active hematological malignancy or recent radiotherapy to the head and neck region EACMFS  Significant valvular heart disease — only when not covered by NICE NG151 (2008, revised 2016) endocarditis prophylaxis guidelines, noting that current NICE guidance does NOT recommend antibiotic prophylaxis solely to prevent infective endocarditis during dental procedures NICE NG151  Hereditary or acquired bleeding disorders associated with prolonged soft tissue hemorrhage and hematoma formation (e.g., severe hemophilia, patients on anticoagulation with supratherapeutic INR) Institutional/EACMFS  Severe neutropenia (ANC <500 cells/µL) AAOMS  Asplenia or functional hyposplenism SDCEP  ⚠️ Critical Note on Infective Endocarditis: NICE guideline NG151 explicitly states that antibiotic prophylaxis against infective endocarditis is not recommended for dental procedures, including extractions, regardless of cardiac risk category. Some jurisdictions (notably the United States — AHA/ADA guidelines) maintain prophylaxis for a restricted group of highest-risk cardiac patients (prosthetic valves, previous IE, certain congenital defects). The clinician must apply the relevant national guideline applicable to their jurisdiction.    B. Surgical Complexity and Procedure-Related Factors  When the operative procedure itself increases the likelihood of postoperative infection beyond the baseline threshold, prophylaxis may be warranted:  • Deeply impacted mandibular third molars requiring extensive osteotomy, significant bone removal, or involving proximity to the inferior alveolar nerve (IAN) — particularly Class III, Position C (Pell and Gregory) or equivalent classifications with anticipated prolonged operative duration (>45–60 minutes)  • Concurrent acute pericoronitis that has been adequately managed prior to surgery — in this context, prophylaxis bridges the residual inflammatory/infectious milieu  • Previous history of postoperative osteitis or surgical site infection in the same region (relative indication)  • Anticipated significant periosteal stripping with compromised soft tissue vascularity  It is important to distinguish prophylaxis (administered in the absence of established infection) from therapeutic antibiotics (indicated when active odontogenic infection, suppuration, or systemic involvement is already present). The latter is not prophylaxis and is always indicated.    C. Concurrent Surgical Procedures  When third molar removal is performed concurrently with:  • Orthognathic surgery (e.g., bilateral sagittal split osteotomy)  • Osseous reconstructive procedures involving bone grafting  • Implant placement in proximity to the surgical site  ...antibiotic prophylaxis protocols follow the more stringent guidelines governing those primary procedures, typically with perioperative systemic coverage.    III. Antibiotic Selection, Dosing, and Timing  When prophylaxis is legitimately indicated, the following regimen reflects current guideline consensus:  First-Line  • Amoxicillin 2g orally administered 30–60 minutes preoperatively (single preoperative dose preferred over postoperative continuation in healthy hosts)  Penicillin Allergy (Non-Anaphylactic)  • Clindamycin 600mg orally 30–60 minutes preoperatively  Penicillin Allergy (Anaphylactic / IgE-mediated)  • Azithromycin 500mg or Clarithromycin 500mg orally, single preoperative dose  • Avoid cephalosporins in confirmed IgE-mediated cross-reactivity  Duration  Current evidence does not support prolonged postoperative antibiotic courses following uncomplicated third molar surgery, even in higher-risk patients. A single preoperative dose is the evidence-based standard wherever prophylaxis is employed. Postoperative continuation beyond 24 hours has not demonstrated superior outcomes and substantially increases resistance risk and adverse events.    IV. Strategies That Complement or Replace Antibiotic Prophylaxis  In patients where prophylaxis is borderline or undesirable, evidence-based adjunctive measures include:  • Chlorhexidine gluconate 0.12–0.2% mouthrinse pre- and postoperatively — proven to reduce alveolar osteitis incidence (Hermesch et al.; multiple RCTs)  • Copious intraoperative irrigation with sterile saline  • Meticulous atraumatic surgical technique — minimizing bone necrosis, soft tissue trauma, and operative time  • Oxidized cellulose or resorbable gelatin haemostatic agents in the socket where appropriate  • Adequate postoperative analgesia to support compliance with oral hygiene protocols    V. Summary of Evidence-Based Decision Algorithm  Asymptomatic Impacted Mandibular Third Molar → Elective Surgical Extraction  │  ▼  Is the patient ASA Class I or II with no immunocompromise?  / \  YES NO  │ │  ▼ ▼  Routine prophylaxis Assess specific  NOT indicated systemic condition  and consult relevant  guideline / specialist    VI. Concluding Remarks  The paradigm governing antibiotic prophylaxis in third molar surgery has unequivocally shifted from reflex prescription to selective, evidence-justified administration. The responsible clinician must conduct a rigorous preoperative risk stratification, integrating the patient's systemic health status, surgical complexity, and operative environment before prescribing any antibiotic agent. Indiscriminate prophylaxis in low-risk patients confers no meaningful clinical benefit, exposes patients to unnecessary adverse effects, and contributes to the global burden of antimicrobial resistance — an outcome entirely inconsistent with the ethical obligations of contemporary surgical practice.  When in doubt, consultation with an infectious disease specialist or the patient's treating physician is advisable prior to any deviation from standard antimicrobial stewardship principles. |
| 2. **Describe the medical management protocol (antibiotics and analgesics) for an oncology patient currently receiving intravenous bisphosphonate therapy (e.g., Zoledronic acid) who presents with Stage 2 Medication-Related Osteonecrosis of the Jaw (MRONJ)?** | Below is a guideline-based, clinically structured, exam-ready answer consistent with current AAOMS-positioned literature and recent multidisciplinary MRONJ recommendations.    Medical Management Protocol for Stage 2 MRONJ in a Patient Receiving IV Zoledronic Acid  Clinical Context  Stage 2 Medication-Related Osteonecrosis of the Jaw (MRONJ) is defined by:  • Exposed necrotic bone (or fistula probing to bone)  • Associated infection (erythema, purulent discharge)  • Painful symptomatic presentation  • No extensive disease beyond alveolar bone  Management in oncology patients receiving IV bisphosphonates (e.g., zoledronic acid) is primarily conservative but infection-controlled and symptom-oriented, with multidisciplinary coordination (oral surgery + oncology + infectious disease).    1. Antibiotic Management (Core Component)  A. First-line systemic antibiotics  Goal: control polymicrobial oral flora (aerobic + anaerobic organisms)  Preferred regimens:  • Amoxicillin–clavulanate (first-line if no allergy)  o Covers streptococci, anaerobes, gram-negative oral flora  • If penicillin-allergic:  o Doxycycline  o OR Azithromycin  o OR Metronidazole + fluoroquinolone (e.g., levofloxacin) in more severe infections  📌 Evidence basis:  • AAOMS and recent reviews recommend systemic antibiotics for Stage 2 MRONJ with infection as mandatory therapy to control disease progression and symptoms.    B. Duration of antibiotics  • Typically 10–14 days initially  • May require repeated or prolonged courses depending on:  o Clinical response  o Persistence of infection  o Sequestrum presence  📌 Important principle:  • Antibiotics are symptom-control therapy, not curative therapy in MRONJ.    C. Adjunct antimicrobial therapy  • 0.12–0.2% chlorhexidine mouth rinses (twice daily)  • Local irrigation of exposed bone if possible    2. Analgesic Management  A. First-line analgesics  • Paracetamol (Acetaminophen)  o Safe, first-line agent  o Can be used regularly (dose adjusted for hepatic function if needed)  • NSAIDs (if no contraindications)  o Ibuprofen or naproxen for inflammatory pain  o Used cautiously due to renal and gastrointestinal risks in oncology patients    B. Moderate–severe pain  • Stepwise escalation:  o Paracetamol + NSAID combination  o If inadequate: weak opioids (tramadol, codeine)  o Severe refractory pain: short-course stronger opioids (e.g., morphine)    C. Key clinical caution  • Avoid long-term high-dose NSAIDs if:  o Renal impairment (common in oncology patients)  o Gastrointestinal risk  o Concurrent corticosteroid use    3. Bisphosphonate (Zoledronic Acid) Consideration  • Discontinuation (“drug holiday”) is controversial  • No strong evidence that stopping bisphosphonates reverses MRONJ  • Decision must be:  o Individualized  o Made with oncologist  o Based on cancer-related skeletal risk vs MRONJ progression  📌 Current consensus:  • Priority is infection control + quality of life, not drug cessation alone    4. Local and Supportive Measures (Essential in Stage 2)  • Oral hygiene optimization  • Chlorhexidine rinses  • Removal of loose sequestra (minimally invasive debridement if needed)  • Avoidance of traumatic prosthesis pressure  • Nutritional support if painful mastication limits intake    5. Clinical Treatment Goals  According to contemporary MRONJ consensus statements:  • Control infection  • Reduce pain  • Stabilize lesion  • Prevent progression to Stage 3 (pathologic fracture, fistula, sinus involvement)    6. Summary (Exam-Oriented)  Stage 2 MRONJ in a patient on IV zoledronic acid is managed with:  Antibiotics  • First line: Amoxicillin–clavulanate  • Alternatives: doxycycline, azithromycin, or metronidazole-based combinations  • Duration: typically 10–14 days, repeat as needed  Analgesia  • First line: Paracetamol  • Add NSAIDs if safe  • Escalate to opioids for severe pain  Adjuncts  • Chlorhexidine rinses  • Local debridement of sequestra if indicated  • Multidisciplinary oncologic coordination | As an Oral and Maxillofacial Surgeon, managing Stage 2 Medication-Related Osteonecrosis of the Jaw (MRONJ) in an oncology patient requires a meticulous, evidence-based approach. The primary goal is to alleviate pain, control secondary infection of the necrotic bone, and prevent further sequestration or progression to Stage 3.  According to the most recent clinical guidelines—including the AAOMS (American Association of Oral and Maxillofacial Surgeons) Position Paper and the MASCC/ISOO/ASCO clinical practice guidelines—the management of Stage 2 MRONJ is primarily non-surgical/conservative, focusing on pharmacological optimization.    1. Antibiotic Protocol  Stage 2 MRONJ is defined by exposed and necrotic bone associated with infection, evidenced by pain and erythema. Antibiotic therapy is directed at reducing the microbial load, specifically targeting the oral commensal flora and Actinomyces species often found in sequestered bone.  Long-Term Suppressive Therapy  Because necrotic bone is avascular, systemic antibiotics cannot easily reach the site. Therefore, extended courses are often necessary.  • First-Line Therapy: Amoxicillin with Clavulanic Acid (875/125 mg) twice daily.  o Alternative: Phenoxymethylpenicillin (500 mg) four times daily.  • In Case of Penicillin Allergy:  o Clindamycin (300 mg) three times daily.  o Metronidazole (500 mg) three times daily (often used in combination with Penicillin to cover anaerobes).  • Refractory Cases: Quinolones like Ciprofloxacin (500 mg) twice daily may be utilized if culture and sensitivity (C&S) testing indicates resistant gram-negative organisms.  Topical Antimicrobials  Systemic therapy must be augmented with local disinfection to reduce the biofilm on the exposed bone.  • Chlorhexidine Gluconate (0.12%) oral rinse used 2–3 times daily.  • Oxidizing mouthwashes (e.g., diluted Hydrogen Peroxide) may be used for debridement of necrotic debris.    2. Analgesic Protocol  Pain management in oncology patients must be balanced against their existing systemic treatments and potential renal/hepatic compromises.  Multimodal Analgesic Strategy  Following the WHO Analgesic Ladder, we aim for the lowest effective dose to maintain quality of life.  Tier Medication Type Clinical Application  Mild Pain Non-Opioids Acetaminophen (Paracetamol) 1g every 6 hours. NSAIDs (e.g., Ibuprofen) should be used with caution if the patient is on concomitant chemotherapy or has renal impairment.  Moderate Pain Weak Opioids Codeine or Tramadol (50–100 mg) every 6 hours, often in combination with Acetaminophen.  Severe Pain Strong Opioids Morphine or Oxycodone. Note: Many oncology patients are already managed by a palliative team; coordination is vital to avoid respiratory depression or drug-drug interactions.    3. Clinical Considerations & Adjuncts  The presence of Stage 2 disease in a patient on Zoledronic acid indicates a high-turnover inhibition of osteoclasts.  • Microbiology: If the infection does not resolve with empirical therapy, a swab for Culture and Sensitivity (C&S) is mandatory to rule out resistant strains or fungal superinfection (Candida).  • Teriparatide (PTH 1-34): While some evidence suggests Teriparatide may assist in bone remodeling, it is strictly contraindicated in oncology patients with a history of skeletal malignancies or bone metastases due to the risk of osteosarcoma.  • Drug Holiday: The "Drug Holiday" for intravenous bisphosphonates remains controversial in the oncology setting. Discontinuation must only be done in consultation with the Medical Oncologist, as the risk of Skeletal Related Events (SREs) like pathological fractures often outweighs the benefits of MRONJ resolution.  Summary of Stage 2 Management Goals  1. Eliminate Pain: Through a structured analgesic ladder.  2. Control Infection: Long-term oral antibiotics and antimicrobial rinses.  3. Conservative Debridement: Remove only sharp edges of bone that cause soft tissue trauma; avoid aggressive surgical resection in the acute inflammatory phase. | Medical Management Protocol for Stage 2 MRONJ in an Oncology Patient Receiving Intravenous Bisphosphonate Therapy    Preamble: Defining the Clinical Context  The scenario presented — an oncology patient receiving intravenous zoledronic acid (ZA) with Stage 2 Medication-Related Osteonecrosis of the Jaw (MRONJ) — represents one of the most clinically demanding intersections in contemporary Oral and Maxillofacial Surgery. The management of this condition demands a multidisciplinary, risk-stratified approach, integrating oncological imperatives, antimicrobial stewardship, pain management, and the biological limitations imposed by compromised osseous vascularity and impaired immune function in the cancer patient.  Primary Authoritative Guideline Sources  • American Association of Oral and Maxillofacial Surgeons (AAOMS) Position Paper on MRONJ — 2022 Update  • Multinational Association of Supportive Care in Cancer / International Society of Oral Oncology (MASCC/ISOO/AAOMS) Consensus Recommendations  • European Association for Osseointegration (EAO) and EACMFS MRONJ guidelines  • National Comprehensive Cancer Network (NCCN) Supportive Care Guidelines  • SIGN / NICE antimicrobial prescribing frameworks  • Ruggiero et al. (2022) — Updated AAOMS staging and management recommendations    I. Staging Confirmation: Stage 2 MRONJ — Diagnostic Criteria  Before establishing a management protocol, precise staging confirmation is essential. According to the AAOMS 2022 classification:  Stage 2 MRONJ is defined as: exposed and necrotic bone, or bone that can be probed through an intraoral or extraoral fistula in the maxillofacial region, with evidence of infection manifested by pain and erythema in the region of the exposed bone with or without purulent drainage, without radiographic evidence of disease extension to the inferior border of the mandible, maxillary sinus, or skin fistulization.  Distinguishing Features from Adjacent Stages  Feature Stage 1 Stage 2 Stage 3  Exposed/necrotic bone Present Present Present  Infection (pain, erythema, suppuration) Absent Present Present  Radiographic extension None None beyond alveolus Pathological fracture / sinus involvement / skin fistula  Systemic symptoms Absent Absent May be present    II. Foundational Pre-Treatment Considerations  A. Multidisciplinary Team (MDT) Coordination  Management must occur within a multidisciplinary framework comprising:  • Oral and Maxillofacial Surgeon — primary procedural and antimicrobial management  • Medical Oncologist — assessment of oncological necessity for continued bisphosphonate therapy, drug holiday feasibility  • Clinical Pharmacist — drug interaction profiling, renal function-adjusted dosing  • Infectious Disease Specialist — refractory infection management, microbiological guidance  • Pain Management / Palliative Care Team — complex analgesic optimization in the oncology context  B. Drug Holiday (Bisphosphonate Cessation): Current Evidence and Recommendations  This remains one of the most debated aspects of MRONJ management. Current guidance reflects the following:  • AAOMS 2022 acknowledges that the benefit of a "drug holiday" (temporary cessation of antiresorptive therapy) has not been conclusively demonstrated in improving MRONJ outcomes, largely due to the prolonged skeletal retention of zoledronic acid (terminal half-life in bone: >10 years)  • However, in consultation with the treating oncologist, temporary suspension of ZA may be considered if the oncological risk of doing so is deemed acceptable — particularly in patients receiving ZA for bone metastasis prophylaxis rather than acute hypercalcemia of malignancy  • Absolute contraindication to drug holiday exists in patients with: active hypercalcemia of malignancy, imminent fracture risk from extensive metastatic bone disease, or hematological malignancies (e.g., multiple myeloma) where bisphosphonate therapy is actively disease-modifying  • The decision must be documented collaboratively with the oncologist, and informed consent must address the inherent oncological risks  Current consensus does not support mandatory drug holidays as a prerequisite for Stage 2 MRONJ management. Surgical and pharmacological intervention should not be delayed pending cessation.  C. Baseline Investigations  Prior to initiating the management protocol, the following investigations are mandatory:  Laboratory:  • Full blood count (FBC) with differential — assess neutropenia, thrombocytopenia  • Comprehensive metabolic panel — renal function critical for antibiotic dosing (ZA is renally cleared; concurrent nephrotoxicity risk)  • Serum calcium, phosphate, alkaline phosphatase  • HbA1c (if diabetic comorbidity)  • Serum C-telopeptide (CTX) — while its predictive value for MRONJ outcomes remains debated, baseline documentation is recommended by AAOMS  • Coagulation studies (PT/INR/aPTT) — relevant if concurrent anticoagulation therapy  Microbiological:  • Deep wound swab or bone biopsy culture (aerobic and anaerobic) with sensitivity profiling — essential to guide targeted antibiotic therapy and distinguish MRONJ from primary osteomyelitis or malignant involvement  • Fungal culture where clinically indicated (immunocompromised host)  Imaging:  • Cone Beam Computed Tomography (CBCT) — primary modality for osseous extent assessment, sequestrum identification, cortical perforation  • Panoramic radiograph — baseline documentation and staging confirmation  • MRI with gadolinium — superior for soft tissue involvement, marrow oedema, and perineural spread assessment in equivocal cases  • Tc-99m bone scintigraphy — optional, useful for multifocal disease assessment in patients with widespread bone metastases    III. Antibiotic Management Protocol  A. Empirical Antibiotic Therapy (Pending Culture Results)  The microbiological environment of MRONJ is characteristically polymicrobial, dominated by oral streptococci, anaerobic gram-negative rods (notably Prevotella, Fusobacterium, Porphyromonas species), and in some cases Actinomyces israelii — the latter being significantly associated with refractory MRONJ.  First-Line Empirical Regimen  Amoxicillin-Clavulanate (Co-Amoxiclav)  • Dose: 875mg/125mg orally twice daily (BID) or 500mg/125mg three times daily (TID)  • Rationale: Broad-spectrum coverage encompassing aerobic streptococci, oral anaerobes, and beta-lactamase producing organisms; established efficacy in odontogenic and osseous infections of the jaws  • Duration: Minimum 4–6 weeks for Stage 2 MRONJ; reassessment at 2-week intervals  • Renal dose adjustment: Required if eGFR <30 mL/min/1.73m² (consult BNF/pharmacist)  ⚠️ In the oncology patient, renal function must be carefully assessed given concurrent ZA nephrotoxicity risk and potential concomitant nephrotoxic chemotherapy agents (e.g., cisplatin, carboplatin).  Penicillin Allergy — Non-Anaphylactic  Clindamycin  • Dose: 300–450mg orally four times daily (QID)  • Rationale: Excellent bone penetration, strong anaerobic and gram-positive coverage  • Critical caveat: Risk of Clostridioides difficile colitis is substantially elevated in oncology patients receiving concurrent or recent broad-spectrum antibiotic therapy, proton pump inhibitors, and chemotherapy-induced gastrointestinal mucosal disruption. Informed consent and active monitoring for C. diff (diarrhoea, abdominal pain, systemic features) are mandatory  • Duration: 4–6 weeks with clinical reassessment  Penicillin Allergy — IgE-Mediated (Anaphylactic History)  Metronidazole + Azithromycin combination:  • Metronidazole 400mg TID (superior anaerobic coverage)  • Azithromycin 500mg once daily for 3–5 days loading, then consider alternative aerobic coverage  • Alternative: Moxifloxacin 400mg once daily — broad-spectrum fluoroquinolone with excellent bone penetration; however, use with caution given QTc prolongation risk in patients receiving concurrent cardiotoxic chemotherapy (anthracyclines) or antiemetics (ondansetron)  B. Culture-Directed (Definitive) Antibiotic Therapy  Upon return of microbiological sensitivity data (typically 48–72 hours), empirical therapy should be de-escalated or targeted accordingly:  • Actinomyces israelii confirmed: Prolonged amoxicillin or penicillin V for 6–12 weeks is the evidence-based standard; Actinomyces-associated MRONJ carries a notoriously refractory course  • MRSA identified: Trimethoprim-sulfamethoxazole (TMP-SMX), doxycycline, or linezolid per sensitivity profile; infectious disease specialist co-management mandatory  • Gram-negative predominance (e.g., Pseudomonas aeruginosa in immunocompromised host): Ciprofloxacin or extended-spectrum penicillins per sensitivity  C. Intravenous Antibiotic Escalation Criteria  Escalation to intravenous antibiotic therapy is indicated when any of the following criteria are met:  • Failure of adequate oral antibiotic therapy after 2–4 weeks of compliance-confirmed treatment  • Inability to tolerate oral medications (severe mucositis, nausea, dysphagia — common in oncology patients)  • Systemic signs of sepsis (pyrexia >38.5°C, tachycardia, raised CRP/ESR/procalcitonin, leukocytosis — or paradoxical leukopenia in neutropenic patients)  • Rapid progression of osseous involvement despite oral therapy  • Deep fascial space involvement or imminent airway compromise  Preferred IV regimen:  • Amoxicillin-Clavulanate IV 1.2g every 8 hours, or  • Piperacillin-Tazobactam 4.5g every 8 hours (broader gram-negative cover in immunocompromised host)  • + Metronidazole 500mg IV every 8 hours if anaerobic burden is clinically significant    IV. Analgesic Management Protocol  A. WHO Analgesic Ladder Application in the MRONJ Context  Pain in Stage 2 MRONJ is characteristically mixed nociceptive and neuropathic in character, arising from: exposed necrotic bone with periosteal nerve irritation, secondary infectious inflammatory mediator release, and — in the oncology context — concurrent disease-related, chemotherapy-induced, or radiotherapy-associated neuropathic pain. Management must therefore address multiple pain mechanisms simultaneously.  Step 1 — Non-Opioid Baseline Analgesia  Paracetamol (Acetaminophen)  • Dose: 1g orally or IV every 6 hours (maximum 4g/24 hours)  • Hepatic dose adjustment: Mandatory in patients with hepatic metastases, hepatotoxic chemotherapy (e.g., methotrexate, FOLFOX), or known hepatic dysfunction — reduce to 500mg every 6 hours or maximum 2g/24 hours  • Rationale: Central and peripheral analgesic effect, antipyretic benefit in febrile MRONJ; well-tolerated and safe in renally impaired patients unlike NSAIDs  NSAIDs — Position Statement:  NSAIDs (e.g., ibuprofen, diclofenac, naproxen) should be used with significant caution or avoided in the following patient subgroups commonly encountered in this clinical scenario:  • Concurrent anticoagulant or antiplatelet therapy (hemorrhagic risk)  • Thrombocytopenia secondary to chemotherapy or bone marrow suppression  • Renal impairment (ZA nephrotoxicity + NSAID nephrotoxicity = compounded tubular damage)  • Gastric mucosal compromise secondary to systemic corticosteroids, chemotherapy-induced mucositis  Where NSAIDs are considered appropriate (eGFR >60, no contraindications): Celecoxib 100–200mg BID (COX-2 selective) with gastroprotection (PPI) represents a safer profile. However, cardiovascular risk assessment is mandatory in oncology patients with pre-existing vascular comorbidities.  Step 2 — Weak Opioid Combination Therapy  When Step 1 analgesia is insufficient (NRS pain score ≥4/10 at rest):  Codeine phosphate 30–60mg orally every 4–6 hours in combination with paracetamol  • Or Tramadol 50–100mg every 6 hours (with caution: serotonergic risk with concurrent 5-HT3 antagonist antiemetics such as ondansetron; seizure threshold lowering)  • Tramadol is relatively contraindicated in patients receiving MAOIs, SSRIs, or SNRIs (serotonin syndrome risk)  Step 3 — Strong Opioid Therapy  Indicated in:  • Breakthrough pain unresponsive to Step 2 agents (NRS ≥7/10)  • Neuropathic component with significant functional impairment  • Patients already on opioid therapy for underlying oncological pain  Morphine sulphate:  • Immediate-release: 5–10mg orally every 4 hours (opioid-naïve adult)  • Modified-release: 10–30mg every 12 hours with rescue immediate-release doses  Oxycodone:  • 5–10mg orally every 4–6 hours; modified-release preparations available  • Preferred over morphine in patients with significant renal impairment due to reduced accumulation of active metabolites  Fentanyl transdermal patches:  • Appropriate for patients with dysphagia, intractable nausea, or established opioid tolerance  • Dose titration must be performed by palliative care/pain management team in oncology patients already on complex analgesic regimens  ⚠️ Opioid prescribing in oncology patients must be coordinated with the treating oncologist and palliative care team to avoid duplication, dose escalation without monitoring, respiratory depression risk, and constipation management (co-prescribe appropriate laxative regimen — e.g., macrogol, senna — with all strong opioids).  B. Neuropathic Pain Adjuvants  Given the frequent neuropathic component in MRONJ (inferior alveolar nerve involvement, perineural invasion by necrotic process):  Gabapentin:  • Starting dose: 300mg at night, titrating over 1–2 weeks to 300–600mg TID  • Renal dose adjustment mandatory — gabapentin is renally eliminated; in eGFR <30, dose reduction and extended intervals required  • Particularly relevant when patients report burning, shooting, or lancinating jaw pain, or inferior lip paraesthesia  Amitriptyline (low-dose):  • 10–25mg at night — effective for neuropathic pain modulation  • Caution: Anticholinergic effects (dry mouth — contraindicated in patients with concurrent xerostomia secondary to head and neck radiation); QTc prolongation risk with concurrent cardiotoxic agents; contraindicated with MAOIs  Pregabalin:  • 75mg BID titrating to 150–300mg BID  • Superior bioavailability profile compared to gabapentin; similar renal adjustment requirements  C. Topical and Local Analgesic Adjuncts  • Chlorhexidine gluconate 0.12–0.2% mouthrinse BID — antimicrobial and mild anti-inflammatory benefit, reduces bacterial biofilm on exposed bone surface; evidence-supported in MRONJ management across all stages  • Topical lidocaine gel (2%) applied to exposed bone margins — short-term procedural analgesia and patient comfort  • Hyaluronic acid-based mucosal dressings — emerging adjunct for soft tissue protection overlying exposed bone    V. Surgical Considerations at Stage 2: Conservative vs. Active  While this question focuses on medical management, it is clinically essential to clarify the surgical decision framework at Stage 2, as surgical intervention directly impacts antibiotic and analgesic requirements:  AAOMS 2022 Recommendation for Stage 2:  • Primary goal: Symptomatic control via antimicrobial therapy, analgesia, and antimicrobial rinses  • Conservative surgical debridement of superficial necrotic bone (sequestrectomy) is recommended to reduce infectious burden, enhance antibiotic penetration, and facilitate soft tissue coverage — without aggressive resection  • Surgical resection (marginal or segmental mandibulectomy) is reserved for Stage 3 or refractory Stage 2 following documented failure of conservative management  • The perioperative antibiotic protocol is as above, with continuation of systemic antibiotics for minimum 2 weeks postoperatively following any surgical debridement    VI. Adjunctive and Emerging Therapeutic Modalities  A. Hyperbaric Oxygen Therapy (HBOT)  • Mechanism: Enhances tissue oxygenation in avascular necrotic bone, promotes angiogenesis, augments phagocytic killing capacity  • Evidence: Limited but accumulating; some case series and retrospective studies demonstrate improved mucosal healing in MRONJ with HBOT  • AAOMS position: Not currently recommended as routine standard of care; may be considered in refractory cases where surgical intervention is contraindicated  • Contraindications in oncology patients: Active thoracic malignancy, uncontrolled pneumothorax, certain chemotherapy agents (bleomycin — oxygen toxicity risk)  B. Platelet-Rich Plasma (PRP) / Platelet-Rich Fibrin (PRF)  • Mechanism: Delivery of concentrated autologous growth factors (PDGF, TGF-β, VEGF) to enhance tissue regeneration and angiogenesis at the necrotic site  • Application: Intraoperative placement within debridement cavity following sequestrectomy  • Evidence: Moderate; multiple case series report improved soft tissue healing and pain reduction; absence of large RCT data  C. Teriparatide (Recombinant PTH 1-34)  • Mechanism: Anabolic bone agent promoting osteoblast activity and bone remodelling  • CRITICAL CONTRAINDICATION: Teriparatide is absolutely contraindicated in patients with bone metastases or skeletal malignancy — its use risks stimulating metastatic growth or inducing hypercalcemia. It therefore has no role in the oncology patient population described in this scenario.  D. Low-Level Laser Therapy (LLLT) / Photobiomodulation  • Anti-inflammatory, analgesic, and tissue-healing properties at the cellular level  • Emerging evidence supports adjunctive use in Stage 1–2 MRONJ; insufficient data for definitive guideline incorporation but may be considered at specialist centres    VII. Monitoring Framework and Treatment Response Assessment  Clinical Response Indicators (Reassess at 2-week intervals)  Parameter Target Response  Pain score (NRS) Reduction ≥50% from baseline  Wound infection signs Resolution of erythema, oedema, purulent discharge  Exposed bone area Stability or reduction (spontaneous sequestration is favourable)  Soft tissue healing Progressive mucosal coverage of exposed bone margins  Systemic inflammatory markers Normalisation of CRP, WBC (where applicable)  Failure Criteria Mandating Protocol Escalation  • Progression to Stage 3 features (pathological fracture, orocutaneous fistula, radiographic inferior border involvement)  • Systemic sepsis despite adequate oral antibiotic therapy  • Intractable pain unresponsive to optimised analgesic protocol  • New or expanding radiographic lucency despite treatment    VIII. Summary Management Algorithm  STAGE 2 MRONJ — IV Bisphosphonate (Zoledronic Acid) — Oncology Patient  │  ┌─────────────┴─────────────┐  MDT REVIEW INVESTIGATIONS  (OMS + Oncology + (FBC, CMP, cultures, CBCT)  Pain + ID Specialist)  │  ┌─────────┴──────────┐  ANTIBIOTIC Rx ANALGESIC Rx  Co-Amoxiclav 875/125mg Paracetamol 1g QDS (base)  BID x 4–6 weeks ± NSAID (if renal/GI safe)  (culture-directed ± Weak opioid (Step 2)  de-escalation) ± Strong opioid (Step 3)  + CHX 0.12% BID ± Neuropathic adjuvant  │  ┌─────────┴──────────┐  DRUG HOLIDAY? CONSERVATIVE  Oncologist decision DEBRIDEMENT  (ZA — usually not (Sequestrectomy  indicated acutely) if accessible)  │  2-WEEK CLINICAL REASSESSMENT  ┌──────────────────────────┐  IMPROVED FAILED  Continue Rx Escalate: IV ABX  reassess 4wks Surgical staging  Consider HBOT/PRF    IX. Concluding Remarks  The management of Stage 2 MRONJ in an oncology patient receiving intravenous zoledronic acid demands a paradigm that simultaneously honours oncological priorities, applies evidence-based antimicrobial stewardship, and provides compassionate pain management within the framework of palliative or curative cancer care. No single pharmacological intervention operates in isolation; the interplay between antibiotic efficacy, renal function, drug interactions, immune status, and quality of life must be continuously re-evaluated.  The clinician's role is not merely to treat the necrotic jaw, but to be an informed and empathetic advocate within the MDT — ensuring that the oral complication of a life-prolonging oncological treatment is managed with the same rigour and evidence base as the underlying malignancy itself.    This protocol reflects current guideline consensus as of 2024–2025. All prescribing decisions must be individualised, verified against current formulary guidance, local antimicrobial resistance patterns, and patient-specific contraindication profiles. Specialist consultation is strongly recommended in all cases. |
| 3. **In a patient with a confirmed penicillin allergy and suspected clindamycin resistance, which antibiotic agents should be prioritized for the empirical treatment of a deep neck infection of odontogenic origin?** | Empirical Antibiotic Selection in Deep Neck Infection of Odontogenic Origin (Penicillin Allergy + Suspected Clindamycin Resistance)  Clinical Context  Deep neck space infections of odontogenic origin are polymicrobial, rapidly progressive, and potentially life-threatening infections involving:  • Streptococci (especially viridans group)  • Anaerobes (Prevotella, Fusobacterium, Peptostreptococcus)  • Occasionally Staphylococcus aureus  Management requires:  • Immediate airway assessment  • Surgical drainage when indicated  • Prompt broad-spectrum intravenous antimicrobial therapy  In this scenario, two major limitations exist:  • True penicillin allergy (beta-lactam avoidance required)  • Suspected clindamycin resistance (loss of traditional alternative agent)    1. First-line Empirical Strategy (Preferred Agents)  A. Respiratory fluoroquinolone-based regimens (core alternative backbone)  These are currently the most widely accepted alternatives in severe beta-lactam–restricted head and neck infections:  Preferred options  • Moxifloxacin (IV or oral)  o Excellent coverage of:   Streptococci   Anaerobes   Many oral Gram-negative organisms  • Levofloxacin + Metronidazole combination  o Levofloxacin → aerobic Gram-negative + streptococci  o Metronidazole → strict anaerobes  📌 This combination is widely recommended in severe odontogenic deep space infections when clindamycin is unreliable.    B. MRSA or severe systemic toxicity risk  If:  • Septic presentation  • Immunocompromised host  • Hospital-acquired risk factors  Add or consider:  • Vancomycin (IV)  o Coverage for MRSA and resistant Gram-positive cocci  • OR Linezolid  o Excellent oral + IV bioavailability  o Strong Gram-positive coverage including MRSA and resistant streptococci    C. Carbapenem-based therapy (selected severe inpatient cases only)  If allergy history is unclear or infection is life-threatening and polymicrobial:  • Meropenem  • Imipenem/cilastatin  📌 Note:  In true IgE-mediated penicillin allergy, carbapenem cross-reactivity is very low but still used cautiously in ICU/hospital settings.    2. Why Clindamycin is Avoided  Although historically first-line, clindamycin is increasingly avoided due to:  • Rising resistance among oral streptococci (S. anginosus group)  • Documented treatment failures in odontogenic infections  • High risk of Clostridioides difficile colitis  Recent clinical data show significantly higher failure rates compared with beta-lactam–based therapy.    3. Evidence-Based Empirical Hierarchy (Current Practical Approach)  In penicillin allergy + suspected clindamycin resistance:  First choice  • Moxifloxacin monotherapy (preferred if no contraindication)  Second choice  • Levofloxacin + Metronidazole  Third choice (severe hospitalized cases)  • Vancomycin + Metronidazole ± Levofloxacin  Fourth-line (ICU / refractory infection)  • Carbapenem-based regimen (e.g., meropenem)    4. Key Clinical Principles (Guideline-Based)  A. Empiric therapy must be broad  Because odontogenic deep neck infections are:  • Polymicrobial  • Anaerobe-dominant in progression phase  B. Antibiotics are adjunctive  Definitive treatment requires:  • Surgical drainage  • Source control (extraction/endodontic elimination)  C. Early escalation is critical  Delay in adequate coverage significantly increases risk of:  • Airway compromise  • Mediastinitis  • Sepsis    5. Final Structured Answer (Exam Format)  In a patient with confirmed penicillin allergy and suspected clindamycin resistance, empirical treatment of odontogenic deep neck infection should prioritize:  1. Moxifloxacin (preferred single-agent fluoroquinolone with anaerobic coverage)  2. Levofloxacin combined with metronidazole (broad aerobic + anaerobic coverage)  3. Vancomycin (for MRSA or severe infection) ± metronidazole or levofloxacin  4. Carbapenems (e.g., meropenem) reserved for critically ill or refractory cases under hospital monitoring  Clindamycin should be avoided due to increasing resistance rates and higher treatment failure in severe odontogenic infections. | Based on current infectious disease guidelines and oral and maxillofacial surgery (OMFS) protocols for severe odontogenic infections, the management of a deep neck space infection (DNSI) in the presence of both a confirmed penicillin allergy and suspected clindamycin resistance requires a strategic, broad-spectrum intravenous (IV) approach.  Odontogenic deep neck infections are typically polymicrobial, driven by a synergistic combination of facultative Gram-positive cocci (primarily Viridans group streptococci) and obligate oral anaerobes (such as Prevotella, Fusobacterium, Peptostreptococcus, and Bacteroides species).  When standard first-line beta-lactams (e.g., Ampicillin-Sulbactam) and traditional alternatives (Clindamycin) are contraindicated or likely to fail, the empirical antibiotic strategy must aggressively target both aerobic and anaerobic components.  Here are the prioritized empirical regimens:  1. First-Line Alternative: Fluoroquinolone + Nitroimidazole  For a patient with a severe (IgE-mediated) penicillin allergy, the most robust and commonly recommended empirical combination is a fluoroquinolone paired with metronidazole.  • Regimen: IV Levofloxacin (750 mg daily) + IV Metronidazole (500 mg every 8 hours)  • Clinical Rationale: Metronidazole provides exceptional, bactericidal coverage against the strict obligate anaerobes involved in odontogenic pathogenesis. However, metronidazole lacks efficacy against aerobic and facultative Gram-positive cocci. Levofloxacin fills this gap by providing excellent coverage against Viridans group streptococci and other facultative organisms.  • Alternative: IV Moxifloxacin (400 mg daily) monotherapy can be considered, as it possesses intrinsic anti-anaerobic activity alongside its Gram-positive coverage. However, in severe, life-threatening deep neck infections, combining a fluoroquinolone with Metronidazole is generally preferred to guarantee comprehensive anaerobic eradication.  2. Escalated Therapy for Critically Ill Patients (MRSA Risk)  If the patient is critically ill, presenting with systemic toxicity, hemodynamic instability, or has a history of recent hospitalization/institutionalization that raises the suspicion for Methicillin-Resistant Staphylococcus aureus (MRSA), the regimen must be escalated.  • Regimen: IV Vancomycin (weight-based dosing, typically 15–20 mg/kg every 8–12 hours) + IV Metronidazole + IV Levofloxacin (or Aztreonam)  • Clinical Rationale: Vancomycin (or alternatively, Linezolid) provides definitive coverage for resistant Gram-positive organisms, including MRSA and highly resistant streptococci. Metronidazole is maintained for the obligate anaerobes. The addition of Levofloxacin or Aztreonam provides necessary Gram-negative and additional facultative coverage without introducing a beta-lactam risk.  3. The Carbapenem Consideration (Depending on Allergy Severity)  It is imperative to stratify the specific nature of the "confirmed penicillin allergy."  • Clinical Rationale: If the allergy is an IgE-mediated anaphylactic reaction, all beta-lactams (including cephalosporins and carbapenems) are typically withheld empirically. However, if the allergy is confirmed to be a mild, non-IgE mediated delayed reaction (e.g., a simple maculopapular rash), current literature and infectious disease guidelines support the cautious use of IV Ertapenem (1g daily) or IV Meropenem.  • The cross-reactivity between penicillins and carbapenems is currently recognized to be exceptionally low (less than 1%). Carbapenems offer excellent, single-agent broad-spectrum coverage for both aerobes and anaerobes in odontogenic infections.  Critical Surgical Imperative  From a surgical standpoint, it must be emphasized that empirical antibiotic therapy is entirely secondary to immediate source control. In the context of a deep neck space infection (e.g., Ludwig's Angina, parapharyngeal, or retropharyngeal space involvement), the definitive treatment is securement of the airway, aggressive surgical incision and drainage (I&D), removal of the odontogenic source, and obtaining deep purulent samples for prompt Culture and Susceptibility (C&S) testing. Empirical regimens must be aggressively de-escalated or modified as soon as the final antibiogram becomes available. | Empirical Antibiotic Management of Deep Neck Infection of Odontogenic Origin in the Context of Confirmed Penicillin Allergy and Suspected Clindamycin Resistance    Preamble: The Clinical Imperative  Deep neck infections (DNI) of odontogenic origin represent a life-threatening surgical emergency with the potential for catastrophic complications including descending necrotising mediastinitis, septic jugular thrombophlebitis (Lemierre's syndrome), carotid artery erosion, and airway compromise. Mortality in cases complicated by mediastinal extension ranges from 11% to 40% in contemporary series, underscoring the absolute necessity for prompt, appropriately targeted empirical antibiotic therapy initiated without delay pending culture results.  The clinical scenario presented — confirmed penicillin allergy combined with suspected clindamycin resistance — eliminates the two most commonly prescribed empirical agents in odontogenic DNI management, necessitating a methodologically rigorous, evidence-informed alternative framework.  Authoritative Guideline Sources  • Infectious Diseases Society of America (IDSA) — Skin and Soft Tissue Infection Guidelines (2014, updated reviews)  • IDSA — Guidelines for the Diagnosis and Management of Diabetic Foot Infections (cross-applicable polymicrobial principles)  • American Head and Neck Society (AHNS) clinical consensus statements  • European Society of Clinical Microbiology and Infectious Diseases (ESCMID) guidelines  • NICE guideline NG184 — Antimicrobial prescribing for acute bacterial infections  • Sanford Guide to Antimicrobial Therapy (current edition)  • British National Formulary (BNF) and Scottish Dental Clinical Effectiveness Programme (SDCEP)  • Published systematic reviews and meta-analyses on DNI microbiology and outcomes (Boscolo-Rizzo et al.; Bahl et al.; Har-El et al.)    I. Microbiological Landscape of Odontogenic Deep Neck Infections  A. Polymicrobial Architecture  Odontogenic DNI characteristically harbours a polymicrobial consortium reflecting the complex ecology of the oral cavity. Accurate empirical antibiotic selection requires an understanding of the expected microbiological milieu:  Predominant Aerobic/Facultative Anaerobic Organisms  Organism Clinical Significance  Streptococcus anginosus group (S. anginosus, S. intermedius, S. constellatus) Most common; forms abscess; may demonstrate reduced penicillin sensitivity  Streptococcus viridans group Common; generally penicillin-sensitive but relevant in resistance context  Staphylococcus aureus (including MRSA) Increasing prevalence; community-acquired MRSA increasingly documented  Streptococcus pyogenes (Group A Strep) Aggressive necrotising potential  Eikenella corrodens Inherently resistant to clindamycin and metronidazole; relevant in odontogenic infections  Predominant Obligate Anaerobic Organisms  Organism Clinical Significance  Prevotella spp. Beta-lactamase producing; anaerobic gram-negative rod  Fusobacterium nucleatum / F. necrophorum Necrotising potential; F. necrophorum — Lemierre's syndrome  Porphyromonas spp. Virulent periodontal pathogen  Peptostreptococcus / Parvimonas micra Gram-positive anaerobic cocci  Prevotella melaninogenica Beta-lactamase producing; clindamycin resistance documented  Actinomyces spp. Refractory chronic deep space involvement  Bacteroides fragilis group Intrinsically resistant to many beta-lactams; produces metallo-beta-lactamase  B. Critical Resistance Patterns Relevant to This Scenario  The clinician must account for the following established resistance profiles when selecting empirical therapy:  • Clindamycin resistance in oral anaerobes (particularly Prevotella and Bacteroides spp.) is well-documented and increasing, with resistance rates ranging from 15–40% in some European and North American centres  • Eikenella corrodens demonstrates intrinsic resistance to clindamycin, metronidazole, and first-generation cephalosporins — a critical consideration in odontogenic infections  • MRSA in community-acquired DNI is increasingly prevalent, particularly in immunocompromised, diabetic, or previously hospitalised patients  • Beta-lactamase production by anaerobic gram-negative rods (Prevotella, Bacteroides) renders amoxicillin monotherapy insufficient — a factor that also impacts the utility of many alternative agents  The simultaneous exclusion of penicillins AND clindamycin in this polymicrobial infection context substantially narrows the empirical treatment options and mandates selection from agents with demonstrated broad-spectrum activity against aerobic streptococci, anaerobes, and potential MRSA, with reliable tissue penetration into the deep neck fascial spaces.  II. Allergy Risk Stratification: A Prerequisite Step  Before proceeding to alternative antibiotic selection, penicillin allergy must be precisely characterised, as this fundamentally governs cross-reactivity risk with cephalosporins and carbapenems:  Allergy Classification Framework (NICE NG183 / IDSA)  Allergy Type Definition Cross-Reactivity Risk with Cephalosporins Cross-Reactivity Risk with Carbapenems  Non-severe / non-IgE-mediated Remote rash, GI intolerance, family history only <2% <1%  Severe non-IgE-mediated Serum sickness, drug fever, maculopapular rash Low-moderate Low  IgE-mediated (immediate hypersensitivity) Urticaria within 1h, angioedema, bronchospasm, anaphylaxis 1–2% with dissimilar R1 side chains <1%  Severe cutaneous adverse reactions (SCAR) SJS, TEN, DRESS, AGEP Absolute contraindication Absolute contraindication  ⚠️ In a life-threatening DNI where carbapenem therapy is the optimal clinical choice and penicillin allergy has not been formally characterised as IgE-mediated or SCAR, the clinical risk-benefit calculation strongly favours proceeding with carbapenem therapy under close monitoring or allergy specialist guidance. The cross-reactivity between penicillins and carbapenems is now established at <1% and is driven by the shared beta-lactam ring only, NOT the side chain — the primary determinant of IgE specificity.    III. Priority Empirical Antibiotic Agents: Evidence-Based Selection  The following agents are presented in order of clinical priority, stratified by allergy severity and clinical context:    TIER 1 — First-Priority Empirical Agents    Agent 1: Meropenem or Imipenem-Cilastatin (Carbapenems)  Classification: Group 1 Carbapenem (Meropenem); Group 2 (Imipenem-Cilastatin)  Dosing:  • Meropenem: 1–2g IV every 8 hours (preferred in CNS risk patients — lower seizure threshold than imipenem)  • Imipenem-Cilastatin: 500mg–1g IV every 6–8 hours  • Dose adjustment required for eGFR <50 mL/min/1.73m²  Spectrum Relevance to Odontogenic DNI:  • Exceptional broad-spectrum activity encompassing: aerobic gram-positive cocci (including most streptococci), gram-negative aerobes, obligate anaerobes (including Bacteroides fragilis group and Prevotella spp.), and beta-lactamase-producing organisms  • Effective against Eikenella corrodens — critical given its intrinsic clindamycin/metronidazole resistance  • Does NOT cover MRSA — requires addition of anti-MRSA agent if MRSA is clinically suspected  Clinical Evidence Base:  • Carbapenems represent the gold standard empirical regimen for polymicrobial severe soft tissue infections and DNI in the penicillin-allergic patient across multiple institutional protocols and IDSA guidance  • Meropenem demonstrates superior CNS safety profile and is preferred in patients with seizure risk (relevant in septic encephalopathy)  Cross-Reactivity Safety:  • Cross-reactivity with penicillin: <1% — clinically acceptable even in confirmed IgE-mediated penicillin allergy (with appropriate monitoring)  • Absolutely contraindicated in confirmed SCAR (SJS/TEN/DRESS) to any beta-lactam  Role in Protocol: Primary empirical backbone agent in haemodynamically compromised or rapidly deteriorating DNI, or when initial antibiotic therapy has failed    Agent 2: Metronidazole — Essential Anaerobic Augmentation  Classification: Nitroimidazole — obligate anaerobic bactericidal agent  Dosing:  • 500mg IV every 8 hours (or 400mg orally TID when oral route feasible)  • No renal adjustment required; hepatic dose reduction in severe hepatic impairment  Spectrum Relevance:  • Bactericidal against virtually all obligate anaerobes including Bacteroides fragilis, Prevotella, Fusobacterium, Clostridium spp.  • No aerobic activity — must always be used in combination  • Critically important: metronidazole's anaerobic coverage complements agents lacking this activity (e.g., fluoroquinolones, glycopeptides)  ⚠️ Metronidazole does NOT cover Eikenella corrodens or facultative aerobes (streptococci, staphylococci). This is a critical limitation requiring awareness.  Combination Role: Metronidazole is a mandatory component of all non-carbapenem empirical regimens for odontogenic DNI, providing anaerobic cover when the primary agent lacks this activity    Agent 3: Moxifloxacin (Fluoroquinolone)  Classification: 4th generation fluoroquinolone — broad-spectrum bactericidal agent  Dosing:  • 400mg IV or orally once daily  • No renal dose adjustment required (biliary elimination) — significant advantage in renally compromised septic patients  • Oral bioavailability approaches 90% — facilitates early IV-to-oral switch  Spectrum Relevance to Odontogenic DNI:  Organism Group Moxifloxacin Activity  Streptococcus spp. (including S. anginosus group) ✅ Excellent  Staphylococcus aureus (MSSA) ✅ Good  MRSA ❌ Unreliable — do not depend upon  Eikenella corrodens ✅ Active — critical advantage  Oral anaerobes (Prevotella, Fusobacterium) ✅ Good — superior to earlier fluoroquinolones  Bacteroides fragilis ✅ Moderate — enhanced vs. ciprofloxacin  Actinomyces spp. ✅ Active  Critical Advantages in this Scenario:  1. No cross-reactivity with beta-lactams or lincosamides — entirely safe in penicillin allergy and clindamycin resistance scenarios  2. Intrinsic activity against Eikenella corrodens — a significant advantage over clindamycin/metronidazole  3. Excellent tissue penetration into soft tissues, bone, and fascia — pharmacokinetically favourable for deep space infections  4. Single daily dosing — compliance advantage  Critical Limitations and Contraindications:  • QTc prolongation risk — absolute contraindication with concurrent QTc-prolonging agents (class IA/III antiarrhythmics, certain antipsychotics, azithromycin); baseline ECG mandatory  • Avoid in patients with known hepatic impairment (hepatic elimination)  • Tendinopathy risk (fluoroquinolone class effect) — counsel patients; avoid in those with prior fluoroquinolone-associated tendon injury  • Increasing resistance in some Streptococcus strains in specific geographic regions — local antibiogram data should inform decisions  • Not reliably effective against MRSA — if MRSA suspected, addition of vancomycin or daptomycin is required  Evidence Base:  • Multiple retrospective studies and case series support moxifloxacin monotherapy efficacy in odontogenic infections including deep space infections (Sobottka et al., 2012; Brescó-Salinas et al., 2006)  • Demonstrated superior anaerobic coverage compared to ciprofloxacin and levofloxacin, making it the preferred fluoroquinolone for odontogenic infections  Role in Protocol: Primary empirical agent for haemodynamically stable patients without MRSA risk; may be used as monotherapy for Stage I–II DNI with early surgical drainage, or in combination with metronidazole for enhanced anaerobic cover in severe cases    TIER 2 — Secondary Empirical Agents (Specific Indications)    Agent 4: Vancomycin  Classification: Glycopeptide — gram-positive bactericidal agent  Dosing:  • Loading dose: 25–30mg/kg IV (actual body weight) for severe sepsis  • Maintenance: 15–20mg/kg IV every 8–12 hours, AUC/MIC-guided dosing (target AUC 400–600 mg·h/L per ASHP/IDSA/SIDP 2020 consensus guidelines) or trough-guided (target 15–20 mg/L for deep infections)  • Mandatory renal function monitoring — vancomycin is nephrotoxic; creatinine and eGFR must be monitored every 24–48 hours  Spectrum Relevance:  • Primary indication: Suspected or confirmed MRSA in the DNI context  • Active against MSSA, streptococci (including S. anginosus group), enterococci (VSE)  • No gram-negative or anaerobic activity — must be combined with metronidazole and a gram-negative active agent  • No activity against Eikenella corrodens  Indications for Inclusion in Empirical Protocol:  • History of prior MRSA colonisation or infection  • Healthcare-associated exposure within preceding 90 days  • Immunocompromised host (HIV, transplant, haematological malignancy)  • Intravenous drug user  • Failure of initial empirical therapy  • Necrotising fasciitis with gram-positive predominance on Gram stain  Combination with Meropenem + Metronidazole: Provides comprehensive coverage including MRSA for the most critically ill patients  Agent 5: Daptomycin  Classification: Lipopeptide — bactericidal against gram-positive organisms  Dosing:  • 6–10mg/kg IV once daily (higher end for severe/bacteraemic infection)  • Renal dose adjustment required for eGFR <30 mL/min  Advantages over Vancomycin:  • Concentration-dependent bactericidal killing — rapid bacterial eradication  • Activity against vancomycin-intermediate S. aureus (VISA) and vancomycin-resistant enterococci (VRE)  • Lower nephrotoxicity profile compared to vancomycin — preferable in patients with pre-existing renal impairment  Critical Limitation:  • Inactivated by pulmonary surfactant — NOT appropriate for pulmonary complications (not relevant to primary DNI management, but relevant if pulmonary aspiration/pneumonia complicates the course)  • No anaerobic or gram-negative activity — requires combination with metronidazole and gram-negative coverage  Role: Alternative to vancomycin for MRSA cover when renal impairment precludes safe vancomycin use, or in VISA/VRE contexts    Agent 6: Linezolid  Classification: Oxazolidinone — bacteriostatic against most organisms (bactericidal against streptococci)  Dosing:  • 600mg IV or orally every 12 hours  • No renal or hepatic dose adjustment required — significant advantage in multiorgan dysfunction  • Oral bioavailability: ~100% — highly suitable for step-down therapy  Spectrum:  • Active against MRSA, MSSA, streptococci, enterococci (including VRE)  • No gram-negative or anaerobic activity — combination with metronidazole and gram-negative agent mandatory  Critical Drug Interactions — Serotonin Syndrome Risk:  • Absolutely contraindicated with concurrent SSRIs, SNRIs, MAOIs, tramadol — serotonin syndrome risk is a potentially fatal interaction  • This drug interaction must be specifically reviewed in the clinical context, as anxiety/depression pharmacotherapy is common in the DNI patient population  Duration Limitation:  • Maximum 28 days of therapy due to cumulative toxicity: myelosuppression (thrombocytopenia, anaemia), peripheral neuropathy, and optic neuropathy with prolonged use  • Weekly full blood count monitoring mandatory  Role: Alternative to vancomycin/daptomycin for gram-positive/MRSA coverage when renal impairment is severe; oral formulation makes it uniquely suitable for prolonged step-down therapy    Agent 7: Tigecycline  Classification: Glycylcycline (tetracycline derivative) — broad-spectrum bacteriostatic agent  Dosing:  • Loading dose: 100mg IV once  • Maintenance: 50mg IV every 12 hours  • No renal adjustment required; reduce in severe hepatic impairment  Spectrum Relevance:  Organism Tigecycline Activity  Streptococcus spp. ✅ Active  MRSA, MSSA ✅ Active  Bacteroides fragilis ✅ Active  Oral anaerobes ✅ Active  Eikenella corrodens ✅ Active  Pseudomonas aeruginosa ❌ Intrinsically resistant  Proteus spp. ❌ Reduced activity  Critical Limitations:  • FDA Black Box Warning: Increased all-cause mortality in severe infections compared to comparators in clinical trials — tigecycline should be reserved for situations where alternative agents are unavailable or contraindicated  • Bacteriostatic, not bactericidal — suboptimal in septicaemia and rapidly progressive infections  • Nausea and vomiting are significant dose-limiting adverse effects (>30% incidence)  • Not recommended as monotherapy in severe DNI with haemodynamic compromise  Role: Reserve agent in extensively drug-resistant polymicrobial DNI where carbapenem use is precluded (e.g., carbapenem allergy, carbapenem-resistant organisms); not first-line    Agent 8: Ceftriaxone (With Careful Allergy Characterisation)  Classification: Third-generation cephalosporin  Dosing: 2g IV once daily (or 1–2g every 12–24 hours in severe infection)  Cross-Reactivity Consideration:  • In non-IgE-mediated penicillin allergy (rash only, remote history): cross-reactivity risk is <1–2% — ceftriaxone is clinically acceptable per NICE NG183 and IDSA guidance  • In confirmed IgE-mediated anaphylaxis: Skin testing or formal allergy assessment recommended; however, in a life-threatening DNI, the risk-benefit ratio may still favour cautious administration under monitoring  • In SCAR: Absolutely contraindicated  Spectrum: Excellent streptococcal coverage, Eikenella corrodens activity, gram-negative cover — but lacks anaerobic coverage; must be combined with metronidazole  Role: Acceptable alternative in non-severe penicillin allergy when beta-lactam use can be cautiously reintroduced; must never be used in confirmed SCAR    IV. Recommended Empirical Antibiotic Regimens — Clinical Decision Framework  Regimen A: Haemodynamically Stable Patient, No MRSA Risk Factors  → Moxifloxacin 400mg IV/oral once daily (monotherapy)  Justification: Broad-spectrum including anaerobes and Eikenella; no cross-reactivity concerns; excellent tissue penetration; single daily dosing  Consider adding Metronidazole 500mg IV every 8 hours for enhanced anaerobic cover in severe anaerobic burden (foul odour, extensive necrotic tissue, gas-forming infection on imaging)    Regimen B: Haemodynamically Compromised / Rapidly Progressive DNI, No MRSA Risk  → Meropenem 1–2g IV every 8 hours + Metronidazole 500mg IV every 8 hours  Justification: Maximum broad-spectrum bactericidal cover; <1% cross-reactivity with penicillin; metronidazole adds synergistic anaerobic augmentation    Regimen C: Haemodynamically Compromised / Suspected MRSA Risk  → Meropenem 1–2g IV every 8 hours + Metronidazole 500mg IV every 8 hours + Vancomycin (AUC-guided dosing)  OR if renal impairment precludes vancomycin: → Meropenem + Metronidazole + Daptomycin 8–10mg/kg IV once daily    Regimen D: Carbapenem Contraindicated (SCAR to All Beta-Lactams) + No MRSA Risk  → Moxifloxacin 400mg IV once daily + Metronidazole 500mg IV every 8 hours    Regimen E: Carbapenem Contraindicated + MRSA Risk  → Moxifloxacin 400mg IV once daily + Metronidazole 500mg IV every 8 hours + Vancomycin (AUC-guided)  OR: → Tigecycline 100mg loading → 50mg every 12 hours (reserve; haemodynamically stable only)    V. Visualised Summary: Antibiotic Selection Algorithm    V  visualize  V  visualize show_widget  VI. Critical Drug Interaction and Safety Considerations  The following interaction profiles are clinically mandatory in this patient population:  Moxifloxacin Safety Matrix  Co-administered Agent Interaction Risk Management  Ondansetron / other 5-HT3 antagonists QTc prolongation (additive) ECG monitoring; consider alternative antiemetic  Antipsychotics (haloperidol, quetiapine) QTc prolongation (additive) Cardiology consultation  Class IA/III antiarrhythmics Potentially fatal arrhythmia Absolute contraindication  Oral anticoagulants (warfarin) Enhanced anticoagulant effect Increased INR monitoring  Systemic corticosteroids Tendinopathy risk augmented Avoid in at-risk patients  Vancomycin Safety Matrix  Parameter Monitoring Requirement  Renal function (eGFR, creatinine) Every 24–48 hours  AUC/MIC target 400–600 mg·h/L  Concurrent nephrotoxins (NSAIDs, contrast, aminoglycosides) Dose adjustment / avoidance  Auditory function Baseline assessment; monitor for ototoxicity    VII. Escalation Criteria Requiring Immediate Intervention  The following constitute absolute escalation triggers mandating surgical and/or intensive care team involvement regardless of antibiotic regimen:  • Progressive airway compromise — anticipate early surgical airway (cricothyroidotomy / tracheostomy)  • Radiographic evidence of mediastinal extension — computed tomographic angiography and urgent cardiothoracic consultation  • Cavernous sinus thrombosis — high-dose IV antibiotics with anticoagulation consideration  • Lemierre's syndrome (septic jugular thrombophlebitis) — anticoagulation debate; infectious disease co-management essential  • Haemodynamic deterioration meeting sepsis-3 criteria — ICU admission, vasopressor support, source control surgery    VIII. Concluding Summary  When both penicillin and clindamycin are unavailable for empirical management of odontogenic DNI, the evidence-based hierarchy prioritises:  1. Moxifloxacin as the primary agent for haemodynamically stable, non-MRSA-risk patients — uniquely covering Eikenella corrodens while respecting both allergy constraints  2. Carbapenems (meropenem) as the backbone for critically ill or rapidly deteriorating patients, leveraging the <1% cross-reactivity with penicillin  3. Metronidazole as an obligatory anaerobic augmentation agent in all non-carbapenem regimens  4. Vancomycin or daptomycin layered upon the above when MRSA is clinically suspected  5. Ceftriaxone as a pragmatic option when penicillin allergy is non-IgE-mediated and the benefit-risk calculation favours beta-lactam reintroduction under monitoring  6. Linezolid and tigecycline reserved as antimicrobial stewardship-compliant alternatives when the above options are pharmacologically precluded  In all cases, empirical therapy must be promptly refined upon culture and sensitivity data, surgical source control remains the cornerstone of definitive management, and multidisciplinary collaboration — particularly with infectious disease specialists — is indispensable in navigating this pharmacologically complex clinical scenario.    This protocol reflects international guideline consensus as of 2024–2025. All antibiotic prescribing must be individualised against local antimicrobial resistance patterns, institutional antibiograms, patient-specific contraindications, and current formulary guidance. |
| 4. **Specify the safe analgesic and antibiotic options for a patient in the second trimester of pregnancy presenting with an acute apical abscess without systemic involvement?** | Safe Analgesic and Antibiotic Management of Acute Apical Abscess in the 2nd Trimester of Pregnancy (No Systemic Involvement)  Clinical Context  A second-trimester pregnant patient with an acute apical abscess without systemic signs (no fever, cellulitis, lymphadenopathy, or trismus) represents a localized odontogenic infection. Management must balance:  • Maternal infection control  • Fetal safety (organogenesis completed; lower teratogenic risk window)  • Avoidance of unnecessary pharmacologic exposure  The second trimester is considered the safest period for dental intervention and pharmacotherapy when required.    1. Primary Principle of Management  Definitive treatment is essential  • Drainage (incision & drainage or endodontic access) is the primary therapy  • Antibiotics are adjuncts only, not definitive treatment in localized abscess    2. Safe Antibiotic Options (Evidence-Based)  First-line antibiotics (preferred in pregnancy)  These agents are widely considered safe (FDA category B profile and guideline-supported):  • Amoxicillin  • Amoxicillin–clavulanic acid  • Penicillin V  • Cephalosporins (e.g., cephalexin)  These are consistently recommended as first-line agents for odontogenic infections in pregnancy due to safety and efficacy against oral streptococci.    Second-line / Penicillin-allergic patients  If true IgE-mediated allergy:  • Clindamycin (effective anaerobic coverage; still acceptable in pregnancy despite resistance concerns)  • Azithromycin (safe macrolide alternative)  • Erythromycin base (not estolate form)    Agents to avoid  • Tetracyclines (absolute contraindication)  o Risk: fetal tooth discoloration and impaired bone development  • Aminoglycosides (e.g., gentamicin) → fetal ototoxicity risk  • Fluoroquinolones → avoid unless life-threatening infection and no alternatives    3. Safe Analgesic Options  First-line analgesic  Paracetamol (Acetaminophen)  • Drug of choice in all trimesters  • Safe fetal profile  • Effective for mild–moderate odontogenic pain  📌 Strong guideline consensus:  Paracetamol is the preferred and safest analgesic throughout pregnancy.    Second-line (if pain not controlled)  Opioids (short-term, cautious use)  • Codeine (limited use)  • Tramadol (short course if required)  Used only when:  • Pain is moderate–severe  • Paracetamol alone is insufficient    NSAIDs (restricted use in 2nd trimester)  • Ibuprofen / naproxen  o May be used only short-term in early–mid second trimester  o Must be avoided later due to:   Premature ductus arteriosus closure   Fetal renal effects   Bleeding risk  📌 NSAIDs are generally avoided or minimized in pregnancy, especially beyond mid-second trimester.    4. Clinical Management Strategy (Integrated Plan)  Step 1 – Source control (mandatory)  • Endodontic drainage OR extraction  • Incision & drainage if fluctuant swelling present  Step 2 – Antibiotic therapy (if indicated)  • Amoxicillin or amoxicillin-clavulanate first line  • Alternatives if allergic: clindamycin or azithromycin  Step 3 – Pain control  • Paracetamol scheduled dosing  • Add short-course NSAID only if necessary and clinically safe  • Escalate to opioid only if refractory pain  Step 4 – Supportive care  • Warm saline rinses  • Oral hygiene optimization  • Close follow-up within 24–48 hours    5. Key Clinical Takeaways  • Second trimester = safest window for dental infection management  • Amoxicillin-based antibiotics are first-line  • Paracetamol is the analgesic of choice  • NSAIDs are restricted, not first-line  • Definitive drainage is more important than antibiotics    Concise Exam Answer  In the second trimester, an acute apical abscess without systemic involvement should be managed primarily with drainage or endodontic intervention. If antibiotics are required, first-line agents include amoxicillin or amoxicillin-clavulanate, with cephalosporins as alternatives; in penicillin-allergic patients, clindamycin or azithromycin may be used. Paracetamol is the analgesic of choice throughout pregnancy. NSAIDs should be avoided or used only short-term with caution due to fetal risks, particularly ductus arteriosus closure and renal effects. Opioids may be considered briefly for severe pain when necessary. | Based on current international guidelines from the American Dental Association (ADA), the American Association of Endodontists (AAE), and the American College of Obstetricians and Gynecologists (ACOG), the management of a pregnant patient in the second trimester with an acute apical abscess requires a rigorous approach balancing maternal-fetal safety with definitive source control and antimicrobial stewardship.  I. The Surgical Imperative and Antimicrobial Stewardship  Before specifying pharmacological agents, it is clinically imperative to address the premise of the intervention. For an acute apical abscess without systemic involvement (i.e., no fever, facial cellulitis, trismus, lymphadenopathy, or malaise), systemic antibiotic therapy is strictly not indicated.  The definitive, evidence-based standard of care is immediate surgical source control. This consists of:  • Incision and drainage (I&D) of the fluctuant swelling.  • Pulp extirpation and canal debridement (endodontic therapy) or extraction of the offending tooth.  Prescribing antibiotics for a localized, uncomplicated apical abscess provides no measurable clinical benefit, exposes the patient and fetus to unnecessary drug risks, and contributes to antimicrobial resistance.  II. Safe Antibiotic Options (Contingency Protocol)  Should the patient’s clinical status deteriorate and systemic signs of infection (e.g., fascial space extension, fever) develop, empirical antibiotic therapy must be initiated. The safest options during the second trimester (historically FDA Pregnancy Category B equivalents) include:  • First-Line Therapy: * Amoxicillin (500 mg PO every 8 hours) or Penicillin V Potassium (500 mg PO every 6 hours). Beta-lactams are highly efficacious against the predominant facultative and anaerobic odontogenic flora and are universally considered safe throughout all trimesters of pregnancy.  • For Penicillin-Allergic Patients:  o Mild/Non-IgE Mediated Allergy: Cephalexin (500 mg PO every 6 hours).  o Severe/IgE-Mediated Anaphylaxis: Clindamycin (300 mg PO every 6 hours) or Azithromycin (500 mg loading dose, followed by 250 mg daily).  • Strictly Contraindicated: Tetracyclines (due to fetal tooth discoloration and impaired bone osteogenesis) and Fluoroquinolones (due to fetal articular cartilage toxicity).  III. Safe Analgesic Options  Pain management in the pregnant patient must rely on agents with the lowest teratogenic and physiologic risk profile.  • First-Line Analgesic: Acetaminophen (Paracetamol)  o Acetaminophen is the analgesic and antipyretic of choice during all stages of pregnancy. It should be utilized at the lowest effective dose for the shortest possible duration (typically up to 3,000 mg/day in divided doses).  • Second-Line Analgesic: NSAIDs (Critical 20-Week Gestation Warning)  o Non-steroidal anti-inflammatory drugs (e.g., Ibuprofen) require extreme caution during the second trimester.  o Gestational Weeks 13 to 19: Short courses (48–72 hours) of Ibuprofen (400 mg every 6 hours) are generally considered safe and effective for severe inflammatory pain.  o Gestational Week 20 and Beyond: The FDA issued a strict warning in 2020 advising against the use of NSAIDs at 20 weeks gestation and later. Use during this period is associated with rare but serious fetal renal dysfunction, leading to oligohydramnios (low amniotic fluid) and potential neonatal renal impairment. Furthermore, NSAIDs are absolutely contraindicated in the third trimester due to the risk of premature closure of the fetal ductus arteriosus.  • Opioids:  o Routine prescription of opioid analgesics (e.g., Codeine, Hydrocodone) should be avoided. If absolute surgical necessity dictates their use for refractory, severe pain, they must be limited to a minimal course (1 to 3 days) to mitigate risks of maternal dependency and neonatal abstinence syndrome.  Clinical Pearl for the OMFS: Optimal periprocedural pain control can significantly reduce postoperative analgesic requirements. The use of Lidocaine 2% with 1:100,000 epinephrine is safe during pregnancy, achieves profound local anesthesia, and minimizes systemic stress and endogenous catecholamine release for the mother. | The second trimester (weeks 13–26) represents the safest period for dental treatment during pregnancy, as organogenesis is largely complete and the risk of preterm labour from interventional procedures is lower than in the third trimester. Definitive treatment (incision and drainage, pulp therapy, or extraction) should never be deferred solely based on pregnancy, as untreated infection poses greater risk to the foetus than appropriately selected pharmacotherapy.  SAFE ANTIBIOTIC OPTIONS:  • Amoxicillin 500 mg orally three times daily for 5–7 days: FDA Category B (no evidence of foetal harm). First-line agent for odontogenic infections in pregnancy. Well-established safety profile.  • Amoxicillin-clavulanate 875/125 mg twice daily: Category B; acceptable when polymicrobial or resistant organisms are suspected. Some guidelines note a theoretical risk of necrotising enterocolitis in neonates with premature rupture of membranes — use with awareness.  • Cefalexin 500 mg four times daily for 5–7 days: Category B; safe alternative, good streptococcal and staphylococcal coverage.  • Metronidazole 400 mg three times daily: Category B; previously avoided in the first trimester; considered safe in the second and third trimesters. Suitable for anaerobic component of mixed infections.  CONTRAINDICATED ANTIBIOTICS:  • Tetracyclines/Doxycycline (Category D): chelate calcium in developing teeth → permanent dental discolouration, inhibition of bone growth.  • Fluoroquinolones (Ciprofloxacin, Moxifloxacin): associated with cartilage toxicity in animal studies; avoid throughout pregnancy.  • Trimethoprim-sulfamethoxazole: folate antagonism (risk of neural tube defects in first trimester; kernicterus risk near term).  SAFE ANALGESIC OPTIONS:  • Paracetamol (Acetaminophen) 1000 mg up to four times daily: traditional drug of choice in pregnancy. Although large epidemiological studies have suggested associations with neurodevelopmental outcomes at chronic high doses, short-term therapeutic use at the lowest effective dose remains the preferred analgesic at all gestational stages.  • Lidocaine with epinephrine (local anaesthesia): FDA Category B; safe for dental procedures throughout pregnancy. Epinephrine in 1:100,000 concentration is acceptable — reduces systemic absorption and prolongs anaesthesia, thereby decreasing total local anaesthetic dose required.  ANALGESICS TO AVOID:  • NSAIDs (Ibuprofen, Naproxen, Diclofenac): Pose risk of premature closure of the ductus arteriosus from 20 weeks gestation; contraindicated in the third trimester. Should be used with caution only under specialist supervision in the second trimester after 20 weeks.  • Opioids: Associated with neonatal opioid withdrawal syndrome, preterm birth, and congenital anomalies; avoid unless no alternative exists and only for shortest duration necessary.  CONCLUSION: Definitive drainage (I&D or endodontic access) is the priority — antibiotics serve as adjuncts. All prescribing decisions should be made in consultation with the patient's obstetrician. |
| 5. **Identify which medications should be preferred and which must be strictly avoided for postoperative analgesia in a patient on Warfarin therapy with an INR of 2.8?** | Postoperative Analgesia in a Patient on Warfarin (INR 2.8): Evidence-Based Pharmacologic Strategy  Clinical Context  A patient with a therapeutic INR of 2.8 on warfarin is at moderate–high risk of bleeding complications, particularly from medications that:  • Impair platelet aggregation  • Damage gastric mucosa  • Interfere with vitamin K–dependent coagulation pathways  • Alter warfarin metabolism (CYP450 interactions)  In oral and maxillofacial surgery, postoperative analgesic selection must prioritize hemostatic safety and drug–drug interaction avoidance.    1. Analgesics to be PREFERRED (Safe Options)  A. Paracetamol (Acetaminophen) – First-line agent  • Drug of choice in warfarinized patients  • Effective for mild–moderate postoperative pain  • Minimal platelet effect  📌 Clinical caveat:  • Prolonged or high-dose use (>2–3 g/day for several days) may potentiate INR elevation via hepatic metabolism interference, therefore:  o Use lowest effective dose  o Short duration preferred  ✔ Considered the safest first-line analgesic in anticoagulated patients    B. Weak opioids (second-line if pain is moderate–severe)  • Tramadol  • Codeine (± paracetamol combinations)  Advantages:  • No direct platelet inhibition  • No clinically relevant effect on INR  Clinical use:  • Reserved for breakthrough pain or insufficient NSAID-free analgesia    C. Strong opioids (if required, short-term only)  • Morphine  • Oxycodone (carefully titrated)  Indications:  • Severe surgical trauma (e.g., multiple extractions, osteotomy)  ✔ No significant effect on coagulation but monitor for:  • Sedation  • Constipation  • Respiratory depression    2. Medications that MUST BE STRICTLY AVOIDED  A. Non-selective NSAIDs (absolute contraindication in most cases)  Examples:  • Ibuprofen  • Diclofenac  • Naproxen  • Ketorolac  • Indomethacin  Mechanisms of risk:  1. Platelet function inhibition (COX-1 suppression)  2. Gastrointestinal mucosal injury → bleeding risk  3. Synergistic hemorrhagic risk with warfarin  📌 Evidence-based consensus:  NSAIDs significantly increase bleeding risk in patients on warfarin and are strongly discouraged in anticoagulated patients.  ✔ Clinical conclusion:  NSAIDs should be avoided in warfarin-treated patients due to additive hemorrhagic risk and GI toxicity.    B. Aspirin (strictly contraindicated for postoperative analgesia)  • Irreversible platelet inhibition  • Prolonged bleeding time  • Synergistic effect with warfarin    C. Metamizole (dipyrone)  • May potentiate bleeding risk in some populations  • Not preferred in anticoagulated patients in many guidelines    D. COX-2 inhibitors (relative caution)  • Celecoxib may have lower GI toxicity  • However:  o Still interacts indirectly with hemostasis  o Not first-line in INR-elevated patients    3. Key Drug–Drug Interaction Considerations  Paracetamol–Warfarin Interaction  • Repeated high-dose use can increase INR  • Mechanism: hepatic CYP and vitamin K cycle interference  • Clinical relevance increases after >3–5 days of regular dosing  Macrolides / antibiotics (contextual note)  • Not analgesics, but important:  o Can increase INR by CYP inhibition (e.g., erythromycin, clarithromycin)    4. Evidence-Based Clinical Strategy (Stepwise Approach)  Step 1 – Baseline analgesia  • Paracetamol scheduled dosing  Step 2 – If insufficient  • Add weak opioid (tramadol or codeine)  Step 3 – Severe pain  • Short-course strong opioid (e.g., oxycodone or morphine)  Step 4 – Monitoring  • Avoid prolonged high-dose paracetamol (>3–4 days at high dose)  • Consider INR monitoring if prolonged analgesic use or antibiotics are introduced    5. Clinical Summary (Exam-Oriented Answer)  In a patient on warfarin therapy with an INR of 2.8, postoperative analgesia should prioritize paracetamol (acetaminophen) as the first-line agent due to its minimal effect on platelet function and coagulation. If pain control is inadequate, weak opioids such as tramadol or codeine may be used, followed by short-term strong opioids in severe cases.  Strictly contraindicated medications include all non-selective NSAIDs (ibuprofen, diclofenac, naproxen, ketorolac, indomethacin) and aspirin due to their significant impairment of platelet aggregation, increased gastrointestinal bleeding risk, and synergistic hemorrhagic interaction with warfarin. COX-2 inhibitors are not preferred as first-line agents in this setting. | Based on current pharmacological guidelines and maxillofacial surgical protocols for the management of anticoagulated patients, prescribing postoperative analgesia for a patient on Warfarin with an INR of 2.8 requires meticulous attention to both pharmacokinetic interactions and pharmacodynamic effects on hemostasis. An INR of 2.8 indicates therapeutic anticoagulation, meaning the patient is at a significantly elevated risk for both localized surgical site bleeding and systemic hemorrhagic complications.  The analgesic strategy must prioritize agents that do not interfere with primary hemostasis (platelet aggregation) or displace Warfarin from plasma proteins, while aggressively avoiding those that do.  1. Medications to Strictly Avoid (Absolute & Relative Contraindications)  Non-Steroidal Anti-Inflammatory Drugs (NSAIDs) and Salicylates Agents such as Ibuprofen, Naproxen, Diclofenac, Ketorolac, and Aspirin must be strictly avoided. The concomitant use of NSAIDs and Warfarin creates a severe, synergistic hemorrhagic risk through three distinct mechanisms:  • Pharmacodynamic Antiplatelet Effect: NSAIDs reversibly (and Aspirin, irreversibly) inhibit cyclooxygenase-1 (COX-1), blocking the synthesis of Thromboxane A2. This completely disables primary platelet plug formation—the patient's only remaining hemostatic defense when secondary hemostasis is blocked by Warfarin.  • Gastrointestinal Mucosal Toxicity: NSAIDs deplete protective gastric prostaglandins. When a gastric erosion occurs in a therapeutically anticoagulated patient, the risk of a catastrophic, life-threatening gastrointestinal bleed increases exponentially (by up to 10-fold compared to Warfarin use alone).  • Pharmacokinetic Interaction: Many NSAIDs are highly bound to serum albumin and can displace Warfarin, leading to a transient, unpredictable spike in free, active Warfarin levels in the bloodstream.  Selective COX-2 Inhibitors (e.g., Celecoxib) While COX-2 inhibitors spare COX-1 and thus do not inhibit platelet aggregation, they are still metabolized by and can competitively inhibit the CYP2C9 hepatic enzyme—the primary pathway for clearing the highly active S-enantiomer of Warfarin. Consequently, they can dangerously elevate the INR and should generally be avoided or used only under extreme caution and strict INR monitoring.  2. Preferred Medications (First-Line and Escalation)  First-Line: Acetaminophen (Paracetamol) Acetaminophen is the foundational analgesic of choice for patients on Warfarin because it lacks antiplatelet properties and does not cause gastrointestinal ulceration. However, it requires strict dosage parameters:  • The Clinical Caveat: While generally safe, Acetaminophen is not entirely devoid of Warfarin interactions. Doses exceeding 2 to 3 grams per day for multiple consecutive days can inhibit the vitamin K cycle (specifically VKORC1) or generate toxic metabolites that interfere with Warfarin metabolism, leading to a gradual but significant elevation in the INR.  • Prescription Protocol: The maximum daily dose must be restricted. The standard recommendation is to prescribe no more than 2,000 to 2,500 mg per day (e.g., 500 mg every 6 hours) for the shortest duration possible (typically 3 to 5 days).  Second-Line (For Breakthrough / Severe Pain): Short-Acting Opioids If surgical pain is severe and refractory to Acetaminophen alone, short-acting opioids may be integrated.  • Preferred Options: Oxycodone or Hydrocodone (often formulated in combination with Acetaminophen, such as Percocet or Vicodin). If using combination drugs, the surgeon must calculate the total daily Acetaminophen intake to ensure it remains below the 2,000–2,500 mg safety threshold.  • Opioids to Use with Caution: Tramadol must be prescribed with caution. Tramadol and its metabolites have documented pharmacokinetic interactions with Warfarin (via CYP2D6 and CYP3A4 pathways) and have been repeatedly shown in literature to precipitously elevate INR in some patients. If Tramadol is strictly required, an INR check within 48-72 hours is highly recommended.  3. Clinical Pearl: Preemptive and Local Anesthetic Management  For a patient on Warfarin, the most effective way to manage postoperative pain without relying on systemic medications is through robust local anesthesia. Utilizing a long-acting amide local anesthetic, such as Bupivacaine 0.5% with 1:200,000 epinephrine as a regional block at the conclusion of the surgical procedure, can provide 8 to 12 hours of profound analgesia. This effectively bridges the patient through the peak of the acute postoperative inflammatory response, drastically reducing the total milligram requirement for systemic analgesics. | Patients on Warfarin anticoagulation therapy (target INR 2.0–3.5) present a significant pharmacological challenge for postoperative analgesic prescribing, as many commonly used analgesics directly interact with the anticoagulant effect of Warfarin through multiple mechanisms.  PREFERRED ANALGESICS:  • Paracetamol (Acetaminophen): Drug of choice for mild-to-moderate postoperative pain. Does not inhibit platelet aggregation or displace Warfarin from plasma protein binding at standard doses. CRITICAL CAVEAT: At doses exceeding 2 g/day in anticoagulated patients, paracetamol can potentiate the anticoagulant effect of Warfarin by inhibiting vitamin K-dependent clotting factor synthesis (CYP1A2/2E1-mediated). Recommended maximum: 2 g/day in Warfarin patients; INR monitoring is advisable if more than a 3-day course is prescribed.  • Tramadol 50–100 mg every 6 hours (if paracetamol is insufficient): Weak opioid with minimal direct effect on coagulation. However, tramadol inhibits serotonin reuptake which may impair platelet aggregation. May very mildly increase INR; monitor. Avoid in patients with concurrent serotonergic medications (SSRI, SNRI) due to serotonin syndrome risk.  ANALGESICS TO STRICTLY AVOID:  • Non-selective NSAIDs (Ibuprofen, Diclofenac, Naproxen, Ketorolac, Indomethacin): CONTRAINDICATED. Mechanism of interaction: (1) Displacement of Warfarin from albumin binding sites → increased free Warfarin concentration → elevated INR; (2) Inhibition of COX-1 → irreversible platelet aggregation inhibition → prolonged bleeding time; (3) Gastric mucosal injury → risk of upper GI haemorrhage. Combined effect can result in catastrophic bleeding events.  • Aspirin (Acetylsalicylic acid): Absolutely contraindicated unless specifically prescribed for cardiac indications (in which case dual anticoagulant/antiplatelet therapy is managed by cardiology). Aspirin causes irreversible platelet inhibition for the platelet lifespan (7–10 days) and displaces Warfarin from protein binding.  CONDITIONAL USE — COX-2 SELECTIVE INHIBITORS (Celecoxib, Etoricoxib):  COX-2 inhibitors have no clinically significant platelet inhibitory effect (as COX-2 is not expressed in platelets). However, celecoxib is a CYP2C9 inhibitor — as Warfarin (S-isomer) is metabolised by CYP2C9, celecoxib may modestly increase INR. Short-course use with INR monitoring is acceptable in selected cases where paracetamol is insufficient and the patient is closely monitored.  CLINICAL RECOMMENDATION:  Paracetamol ≤2 g/day as the cornerstone of analgesia. If required, a brief course of tramadol may be added with INR review at day 3–5. Any analgesic change in a Warfarin patient mandates INR monitoring within 3–5 days. The anticoagulation clinic or prescribing physician should be notified of any new analgesic prescription. |
| 6. **How should a postoperative pain management plan be structured for a patient with liver failure (Child-Pugh Class B) to minimize the risk of hepatotoxicity?** | Postoperative Analgesia in Child-Pugh Class B Liver Failure: Evidence-Based Structured Approach  Clinical Context  A patient with Child–Pugh Class B hepatic impairment has:  • Moderately reduced hepatic metabolic capacity  • Impaired phase I and/or phase II drug metabolism  • Increased risk of drug accumulation, sedation, and hepatotoxicity  • Altered protein binding and first-pass metabolism  Postoperative analgesic planning must therefore prioritize:  • Hepatic safety  • Avoidance of drug accumulation  • Opioid-sparing multimodal analgesia  • Dose reduction and extended dosing intervals    1. Core Principle: Multimodal, Opioid-Sparing Strategy  Current perioperative pain guidelines emphasize multimodal analgesia to reduce systemic drug toxicity and opioid requirement.  In hepatic impairment, this principle becomes critical due to limited metabolic reserve.    2. First-Line Analgesic (Preferred Agents)  A. Paracetamol (Acetaminophen) – First-line with dose restriction  • Safest systemic analgesic in chronic liver disease when appropriately dosed  • Mechanism: central COX modulation, minimal platelet effect  Recommended use in Child-Pugh B:  • Maximum: ≤2–3 g/day (reduced dose)  • Shortest effective duration  • Avoid prolonged high-dose administration  📌 Rationale:  • Hepatic metabolism via glucuronidation/sulfation pathways  • Toxicity risk increases when CYP2E1 pathway is upregulated or glutathione depleted  Clinical literature supports dose-reduced paracetamol as safe in chronic liver disease when not exceeding limits.    B. Regional anesthesia (highly recommended adjunct)  • Local infiltration  • Peripheral nerve blocks  • Wound infiltration with long-acting local anesthetics  ✔ Advantages:  • Minimizes systemic drug exposure  • Reduces opioid requirement significantly    3. Second-Line Analgesics (Use with Caution)  A. Opioids (carefully titrated, reduced dosing)  Preferred agents:  • Fentanyl (least affected by hepatic metabolism)  • Hydromorphone (dose-reduced)  • Low-dose morphine (with caution)  Key adjustments:  • Reduce dose and/or extend dosing interval  • Close monitoring for:  o Sedation  o Respiratory depression  o Hepatic encephalopathy  📌 Pharmacological rationale:  • Many opioids show reduced clearance and increased bioavailability in liver disease, leading to accumulation risk  ⚠️ Clinical warning:  • Avoid continuous high-dose opioid regimens  • Avoid long-acting formulations in unstable hepatic function    B. NSAIDs (generally avoided in Child-Pugh B)  Why they should be avoided:  • Reduced prostaglandin synthesis → renal hypoperfusion  • Increased risk of:  o Hepatorenal syndrome  o GI bleeding  o Sodium/water retention  📌 In chronic liver disease, NSAIDs are widely discouraged except in exceptional circumstances.    4. Medications to STRICTLY AVOID  A. High-hepatic-risk opioids  • Meperidine (pethidine) → neurotoxic metabolite accumulation  • High-dose morphine infusions without monitoring  • Codeine (unpredictable metabolism due to CYP2D6 variability + hepatic impairment)    B. Hepatotoxic adjuncts  • High-dose or prolonged paracetamol (>3 g/day)  • Combination products with hidden acetaminophen doses  • Sedative co-medications increasing encephalopathy risk (benzodiazepines unless essential)    C. NSAIDs  • Ibuprofen, diclofenac, naproxen, ketorolac → contraindicated or strongly discouraged    5. Stepwise Clinical Pain Management Plan  Step 1 – Baseline (foundation therapy)  • Paracetamol ≤2–3 g/day  • Scheduled dosing (not PRN alone)  Step 2 – Local/regional techniques  • Nerve blocks / infiltration analgesia  • Long-acting local anesthetics (bupivacaine, ropivacaine)  Step 3 – Breakthrough pain  • Low-dose opioid:  o Fentanyl preferred if IV access available  o Hydromorphone or reduced-dose morphine if oral route required  Step 4 – Monitoring  • Monitor for:  o Sedation scale changes  o Signs of encephalopathy  o Respiratory depression  • Avoid polypharmacy with CNS depressants    6. Key Clinical Takeaways  • Paracetamol (dose-reduced) remains first-line even in liver disease  • NSAIDs are generally contraindicated in Child-Pugh B  • Opioids require dose reduction and careful titration  • Regional anesthesia is essential to reduce systemic drug burden  • Drug accumulation risk is the central therapeutic concern    Concise Exam-Style Answer  In a patient with Child-Pugh Class B liver failure, postoperative pain management should be based on a multimodal, opioid-sparing strategy. Paracetamol remains the first-line analgesic but must be dose-reduced to a maximum of 2–3 g/day with short duration of use. Regional anesthesia techniques should be prioritized to minimize systemic drug exposure. If additional analgesia is required, opioids such as fentanyl or carefully titrated hydromorphone may be used at reduced doses with extended dosing intervals and close monitoring for sedation and hepatic encephalopathy. NSAIDs are generally contraindicated due to the risk of renal impairment, gastrointestinal bleeding, and hepatorenal syndrome. Drugs with high hepatotoxic potential or unpredictable metabolism should be strictly avoided. | The management of postoperative pain in a patient with Child-Pugh Class B (moderate) liver cirrhosis presents a complex pharmacokinetic and pharmacodynamic challenge. Hepatic impairment alters drug metabolism due to portosystemic shunting, decreased cytochrome P450 (CYP) enzymatic activity, reduced hepatic blood flow, and hypoalbuminemia (which increases the free fraction of highly protein-bound drugs). Furthermore, these patients are at a high risk for precipitating hepatic encephalopathy, spontaneous bacterial peritonitis, variceal hemorrhage, and hepatorenal syndrome.  A meticulously structured, multimodal pain management plan is required, prioritizing the following protocols:  1. The Foundation: Maximizing Locoregional Anesthesia  To minimize the requirement for systemically metabolized analgesics, the surgical strategy must heavily rely on prolonged local anesthesia.  • Agent Selection: Long-acting amide local anesthetics, such as Bupivacaine (0.5% with epinephrine), administered as regional nerve blocks at the conclusion of the surgery, provide extended postoperative analgesia.  • Clinical Caveat: Because amide local anesthetics undergo hepatic biotransformation, their clearance is delayed in Child-Pugh B patients. The maximum calculated toxic dose must be conservatively reduced by 30% to 50% to prevent Local Anesthetic Systemic Toxicity (LAST).  2. Strictly Contraindicated: Non-Steroidal Anti-Inflammatory Drugs (NSAIDs)  All NSAIDs (including selective COX-2 inhibitors) are absolutely contraindicated in patients with established cirrhosis. Their use is associated with catastrophic complications:  • Hepatorenal Syndrome (HRS): Cirrhotic patients rely heavily on renal vasodilatory prostaglandins (PGE2 and PGI2) to maintain renal perfusion in the setting of splanchnic vasodilation. NSAID-induced inhibition of these prostaglandins drastically reduces renal blood flow, precipitating acute renal failure (HRS).  • Hemorrhage: NSAIDs inhibit platelet aggregation and induce gastrointestinal mucosal ulceration. In a patient with pre-existing portal hypertension, esophageal/gastric varices, and baseline coagulopathy (decreased synthesis of vitamin K-dependent clotting factors), NSAIDs pose an unacceptable risk of fatal gastrointestinal hemorrhage.  3. First-Line Systemic Analgesia: Dose-Adjusted Acetaminophen (Paracetamol)  Despite the historical stigma of its hepatotoxic potential, Acetaminophen remains the safest and preferred first-line analgesic for cirrhotic patients when dosed correctly.  • Pharmacology: Acetaminophen avoids the renal, gastrointestinal, and platelet toxicities inherent to NSAIDs. However, because chronic liver disease depletes hepatic glutathione stores, the threshold for the accumulation of the toxic intermediate metabolite NAPQI (N-acetyl-p-benzoquinone imine) is lowered.  • Prescription Protocol: The maximum daily dose must be strictly capped. Current hepatology guidelines recommend limiting Acetaminophen to 2,000 mg per day (e.g., 500 mg every 6 hours or 1,000 mg every 12 hours) for short, acute postoperative durations (maximum 3 to 5 days).  4. Second-Line Escalation: Opioid Analgesics (Use with Extreme Caution)  If Acetaminophen and locoregional blocks are insufficient, opioids may be considered, but they carry a severe risk of precipitating hepatic encephalopathy and respiratory depression due to decreased hepatic clearance, increased bioavailability (due to portosystemic shunting bypassing first-pass metabolism), and an exaggerated central nervous system response.  • Agents to Strictly Avoid: * Codeine and Tramadol: These are prodrugs that require hepatic biotransformation (via CYP2D6) to their active metabolites. In liver failure, this conversion is impaired, rendering them ineffective while simultaneously allowing toxic parent-drug accumulation.  o Meperidine (Pethidine): Accumulation of the metabolite normeperidine can cause neurotoxicity and seizures.  • Preferred Agents (If Absolute Necessity Dictates): * Fentanyl (if managed in an inpatient setting) is preferred due to its lack of toxic metabolites and relatively short duration of action.  o If an oral opioid is required, Hydromorphone is generally preferred over Morphine or Oxycodone, as it relies more on glucuronidation rather than the CYP450 system.  o Dosing Rule: The opioid dosage should be reduced by 50%, and the dosing interval should be doubled (e.g., prescribing every 8 to 12 hours instead of every 4 to 6 hours) to account for the prolonged elimination half-life. Continuous clinical monitoring for altered mental status is mandatory. | Child-Pugh Class B hepatic impairment (score 7–9) indicates moderate liver failure with significantly reduced synthetic function, impaired cytochrome P450 activity, and decreased plasma protein synthesis. Nearly all analgesic drugs require major dosing modifications in this context due to altered first-pass metabolism, reduced albumin-mediated protein binding, and risk of precipitating hepatic encephalopathy.  PARACETAMOL (Acetaminophen):  Paradoxically, paracetamol remains the analgesic of choice in chronic liver disease when used at reduced doses. The hepatotoxic metabolite NAPQI (N-acetyl-p-benzoquinone imine) is generated by CYP2E1/1A2 — in chronic liver disease, glutathione reserves are reduced, increasing susceptibility to NAPQI-mediated injury. RECOMMENDED DOSE in Child-Pugh B: 500–1000 mg every 8 hours (maximum 2 g/day). Strict avoidance of alcohol essential. Do NOT withhold entirely, as pain itself may worsen portal hypertension through sympathetic activation.  NSAIDs — CONTRAINDICATED in Child-Pugh B and C:  Mechanisms of harm: (1) Reduction of prostaglandin-mediated renal afferent arteriolar tone → acute kidney injury (hepatorenal syndrome trigger); (2) Inhibition of COX-1 → impaired platelet aggregation (compounding existing coagulopathy); (3) Gastric mucosal injury (compounded by portal hypertensive gastropathy); (4) Sodium and water retention → worsening ascites and oedema.  OPIOIDS:  • Opioids undergo extensive hepatic first-pass metabolism and produce active/toxic metabolites. In Child-Pugh B: (1) Increased oral bioavailability (reduced first-pass effect); (2) Prolonged half-life; (3) Risk of precipitating hepatic encephalopathy via increased GABA-ergic tone.  • Tramadol: Reduce dose by 50%; maximum 50 mg every 12 hours. Avoid prolonged use. Active metabolite (O-desmethyltramadol) accumulates.  • Codeine: Avoid — unpredictable CYP2D6 conversion to morphine; accumulation risk.  • Morphine/Oxycodone: Use with extreme caution only for severe uncontrolled pain; lowest effective dose with extended dosing intervals; monitor for encephalopathy, respiratory depression.  ADJUNCTIVE AND NON-PHARMACOLOGICAL STRATEGIES:  • Long-acting regional/local anaesthesia: continuation of surgical site local anaesthetic infiltration (lidocaine, bupivacaine) to minimise systemic analgesic requirements.  • Corticosteroids (Dexamethasone): anti-inflammatory and anti-oedematous effect without direct hepatotoxicity; use with awareness of fluid retention and hyperglycaemia.  • Cold therapy, elevation, and wound care protocols to minimise inflammatory stimulus.  MONITORING:  Liver function tests (ALT, AST, bilirubin, INR) and renal function at baseline and 72-hour post-procedure intervals. All analgesic prescribing decisions should involve the hepatologist. |
| 7. **Explain the clinical implications and pharmacokinetic interaction between carbamazepine, used for trigeminal neuralgia, and macrolide antibiotics?** | Carbamazepine–Macrolide Antibiotic Interaction: Pharmacokinetic Mechanisms and Clinical Implications in Oral and Maxillofacial Practice  Clinical Context  Carbamazepine is widely used for trigeminal neuralgia due to its voltage-gated sodium channel–blocking properties. Macrolide antibiotics (e.g., erythromycin, clarithromycin, azithromycin) are frequently prescribed in odontogenic infections.  This combination is clinically significant because it can lead to rapid, clinically relevant carbamazepine toxicity, particularly in outpatient maxillofacial infection management.    1. Pharmacokinetic Interaction (Core Mechanism)  A. CYP3A4 inhibition  Carbamazepine is primarily metabolized in the liver via:  • Cytochrome P450 3A4 (CYP3A4)  Macrolide effect:  • Erythromycin and clarithromycin = strong CYP3A4 inhibitors  • This leads to:  o ↓ hepatic metabolism of carbamazepine  o ↑ serum carbamazepine concentration  o ↓ clearance and prolonged half-life  📌 Result: acute or subacute drug accumulation    B. Azithromycin exception  • Azithromycin has minimal CYP3A4 inhibition  • Therefore:  o Much lower interaction risk  o Preferred macrolide if a macrolide is unavoidable    C. Secondary mechanism (minor but relevant)  • Reduced formation of carbamazepine-10,11-epoxide (active metabolite)  • Disruption of autoinduction equilibrium → unpredictable plasma levels    2. Clinical Consequences of the Interaction  Carbamazepine toxicity may develop within 24–72 hours of macrolide initiation.  A. Neurological toxicity (most common)  • Dizziness  • Diplopia  • Ataxia  • Nystagmus  • Dysarthria  • Confusion  B. Severe toxicity (high-risk cases)  • Seizures paradoxically (rare)  • Coma  • Respiratory depression  C. Systemic manifestations  • Hyponatremia (SIADH potentiation)  • Cardiac conduction disturbances (rare)  • Hepatic dysfunction (in severe accumulation)  📌 Clinical pearl:  Toxicity can occur even at previously “therapeutic” carbamazepine doses due to sudden CYP inhibition.    3. Clinical Significance in Oral and Maxillofacial Surgery  This interaction is particularly relevant because:  • Odontogenic infections often require empirical antibiotics  • Macrolides are commonly chosen in:  o Penicillin allergy  o Soft tissue infections  • Carbamazepine is frequently used chronically in:  o Trigeminal neuralgia patients presenting for dental care    4. Evidence-Based Antibiotic Strategy in These Patients  A. Antibiotics to avoid  • Erythromycin (strong CYP3A4 inhibitor)  • Clarithromycin (strong CYP3A4 inhibitor)  B. Preferred alternatives  • Azithromycin (minimal CYP interaction)  • Amoxicillin or amoxicillin-clavulanate (if no allergy)  • Clindamycin (if susceptibility confirmed, but resistance considerations apply)  C. Severe infection scenario  • Use non-macrolide broad-spectrum regimens:  o β-lactam combinations (if tolerated)  o Metronidazole combinations where appropriate    5. Management of Established Interaction  If macrolide must be used despite risk:  Monitoring strategy  • Baseline and follow-up carbamazepine serum levels  • Clinical neurological monitoring  Dose adjustment  • Carbamazepine dose reduction may be required (individualized)  • Close supervision due to narrow therapeutic index  Alternative approach  • Prefer switching antibiotic rather than adjusting carbamazepine    6. Key Pharmacological Principle  This is a classic:  “CYP3A4 inhibition → increased carbamazepine plasma concentration → dose-dependent neurotoxicity”  Carbamazepine also exhibits:  • Autoinduction of metabolism (complex nonlinear kinetics)  • Narrow therapeutic window → small changes in clearance = toxicity risk    7. Clinical Summary (High-Yield Exam Answer)  Carbamazepine is metabolized primarily via hepatic CYP3A4, and macrolide antibiotics—particularly erythromycin and clarithromycin—are potent inhibitors of this enzyme. Co-administration results in decreased carbamazepine metabolism, increased serum concentrations, and a significant risk of neurotoxicity, including dizziness, ataxia, diplopia, and potentially severe central nervous system depression. Azithromycin has minimal CYP3A4 inhibitory activity and is therefore the safest macrolide alternative when antibiotic therapy is required. In oral and maxillofacial practice, this interaction is clinically critical in patients with trigeminal neuralgia presenting with odontogenic infections, and macrolides should generally be avoided in favor of non-interacting antibiotics whenever possible. | Based on current pharmacological literature and clinical guidelines, the interaction between carbamazepine and specific macrolide antibiotics represents a classic, highly predictable, and potentially severe pharmacokinetic contraindication that every Oral and Maxillofacial Surgeon must navigate when treating patients with trigeminal neuralgia.  1. The Pharmacokinetic Mechanism  Carbamazepine (CBZ), the first-line pharmacotherapy for the management of trigeminal neuralgia, possesses a narrow therapeutic index. It is heavily metabolized in the liver primarily by the cytochrome P450 3A4 (CYP3A4) isoenzyme to its active metabolite, carbamazepine-10,11-epoxide.  Older generation and specific macrolide antibiotics—most notably Erythromycin and Clarithromycin—are potent, mechanism-based inhibitors of CYP3A4. When administered concurrently, these macrolides form inactive complexes with the CYP3A4 enzyme, drastically reducing its metabolic capacity.  Consequently, the hepatic clearance of carbamazepine is precipitously reduced, leading to a rapid accumulation of the drug and its epoxide metabolite in the systemic circulation, pushing serum levels well into the supratherapeutic and toxic ranges within 24 to 48 hours of co-administration.  2. Clinical Implications: Acute Carbamazepine Toxicity  Because carbamazepine primarily functions by stabilizing voltage-gated sodium channels in the central nervous system, acute toxicity manifests prominently with profound, dose-dependent neurological deficits. If a patient on a stable dose of CBZ is prescribed Erythromycin or Clarithromycin for an odontogenic infection, the surgeon must anticipate the following signs of acute toxicity:  • Vestibular/Ocular Dysfunction: Nystagmus, diplopia (double vision), and profound dizziness.  • Cerebellar/Motor Deficits: Ataxia (loss of coordination, leading to a high fall risk), dysarthria (slurred speech), and generalized motor restlessness.  • Cognitive Impairment: Confusion, severe somnolence, and lethargy, which can rapidly progress to stupor or coma in severe cases.  • Systemic Complications: Nausea, vomiting, and potentially exacerbated hyponatremia (due to CBZ's SIADH-like effect on antidiuretic hormone).  3. Evidence-Based Clinical Management and Alternatives  From an antimicrobial stewardship and patient safety perspective, Erythromycin and Clarithromycin are strictly contraindicated in patients actively taking carbamazepine.  If the patient requires empirical therapy for an odontogenic infection (e.g., due to a confirmed penicillin allergy where a macrolide might typically be considered), the clinical protocol is as follows:  • The Macrolide Exception: Azithromycin (500 mg loading dose, followed by 250 mg daily) is the only macrolide considered safe in this clinical scenario. Unlike erythromycin and clarithromycin, azithromycin is a 15-membered ring macrolide that does not significantly bind to or inhibit the CYP3A4 isoenzyme. Therefore, it does not alter carbamazepine pharmacokinetics.  • Alternative Classes: Depending on the severity of the infection and the precise nature of the patient's allergy, alternative antimicrobial agents such as Clindamycin, Fluoroquinolones (e.g., Moxifloxacin), or later-generation Cephalosporins (if the allergy is non-IgE mediated) should be prioritized to entirely avoid this cytochrome P450 metabolic interaction. | Carbamazepine is a first-line agent for the pharmacological management of trigeminal neuralgia, acting as a voltage-gated sodium channel blocker. It is primarily metabolised by the hepatic cytochrome P450 isoenzyme CYP3A4 into its active metabolite, carbamazepine-10,11-epoxide. Macrolide antibiotics (erythromycin, clarithromycin) are potent CYP3A4 inhibitors. Azithromycin is a weaker inhibitor.  PHARMACOKINETIC MECHANISM OF INTERACTION:  When a macrolide antibiotic inhibits CYP3A4, hepatic and intestinal clearance of carbamazepine is significantly reduced. This results in: (1) A marked increase in plasma carbamazepine concentrations (up to 50–100% increase in AUC); (2) Accumulation of the active epoxide metabolite, which independently causes CNS toxicity; (3) Inhibition of carbamazepine's autoinduction of CYP3A4 (carbamazepine normally induces its own metabolism over 2–4 weeks — macrolides block this pathway).  CLINICAL MANIFESTATIONS OF CARBAMAZEPINE TOXICITY:  • Neurological: diplopia, nystagmus, ataxia, dizziness, dysarthria, confusion, and somnolence (the hallmark triad of carbamazepine toxicity).  • Cardiovascular: cardiac conduction disturbances (AV block, QRS prolongation), arrhythmias in severe toxicity.  • Gastrointestinal: nausea, vomiting, paralytic ileus.  • Haematological: agranulocytosis and aplastic anaemia (rare but life-threatening; risk may increase with elevated plasma levels).  • Electrolyte: hyponatraemia (SIADH — syndrome of inappropriate ADH secretion, a known class effect of carbamazepine).  ADDITIONAL NOTE — BIDIRECTIONAL INTERACTION:  Carbamazepine also induces CYP3A4, CYP1A2, and CYP2C9. Co-administration with macrolides (particularly clarithromycin) can, paradoxically, first cause carbamazepine toxicity (from macrolide-mediated CYP3A4 inhibition) before autoinduction compensates — the net effect in the short term is toxicity. Additionally, carbamazepine may reduce the plasma concentration of co-administered doxycycline (a tetracycline antibiotic used in oral infections) through CYP1A2 induction, reducing doxycycline efficacy.  CLINICAL RECOMMENDATION:  This combination should be AVOIDED. When antibiotic therapy is required in a patient receiving carbamazepine for trigeminal neuralgia, safe alternatives include: Amoxicillin or Amoxicillin-clavulanate (no CYP3A4 interaction), Metronidazole (minor CYP interaction, use with monitoring), or Clindamycin (use with caution — some CYP2C8 interaction but no significant CYP3A4 inhibition). If macrolide use is unavoidable, carbamazepine serum level monitoring and dose reduction should be performed urgently, and the patient should be warned of signs of toxicity. |
| 8. **Detail the appropriate postoperative pain management strategy for asthma patients who have a history of bronchospasm induced by aspirin or other NSAIDs?** | Postoperative Pain Management in Asthmatic Patients with NSAID/ASA-Induced Bronchospasm (AERD / NSAID-Exacerbated Respiratory Disease)  Clinical Context  Patients with a history of bronchospasm triggered by aspirin or non-selective NSAIDs are classified as having:  • NSAID-exacerbated respiratory disease (NERD / AERD)  This phenotype is characterized by:  • Chronic asthma  • Nasal polyposis/rhinosinusitis (often)  • Acute bronchospastic reactions after COX-1 inhibition  The underlying mechanism is:  • COX-1 inhibition → decreased prostaglandin E2 (bronchoprotective)  • Shunting of arachidonic acid metabolism toward leukotrienes → bronchoconstriction    1. Core Principle of Management  Absolute avoidance of COX-1 inhibiting NSAIDs  • Even small doses may trigger severe bronchospasm  • Cross-reactivity is pharmacological (not IgE-mediated)    2. First-Line Postoperative Analgesic Strategy  A. Paracetamol (Acetaminophen) – First-line agent  • Safest and most widely recommended analgesic  • Minimal effect on cyclooxygenase pathways at therapeutic doses  Clinical use:  • Regular scheduled dosing (not PRN-only in early postoperative phase)  • Effective for mild–moderate postoperative pain  📌 Important caveat:  • High doses (>1,000 mg single dose) may rarely trigger reactions in highly sensitive patients, but this is uncommon clinically    B. Regional and local analgesia (critical adjunct)  • Long-acting local anesthetics (e.g., bupivacaine, ropivacaine)  • Inferior alveolar nerve blocks  • Local infiltration techniques  ✔ Strongly recommended to minimize systemic drug requirement    3. Second-Line Analgesics (If Pain is Moderate–Severe)  A. Weak opioids  • Tramadol  • Codeine (or combination preparations with paracetamol)  Advantages:  • No COX inhibition  • No bronchospasm induction    B. Strong opioids (short-term, controlled use)  • Morphine  • Oxycodone  • Fentanyl (hospital setting)  Clinical considerations:  • Titrate carefully  • Monitor for:  o Respiratory depression (especially in severe asthma)  o Sedation  📌 Important distinction:  Opioids do NOT trigger AERD bronchospasm but can suppress respiratory drive at high doses.    4. NSAIDs and COX-2 Inhibitors  A. Strictly contraindicated  • Ibuprofen  • Diclofenac  • Naproxen  • Ketorolac  • Aspirin  ✔ These are COX-1 inhibitors and major bronchospasm triggers    B. COX-2 selective inhibitors (conditional use only)  • Celecoxib  • Etoricoxib (where available)  Clinical evidence:  • Generally safe in AERD due to minimal COX-1 inhibition  • However:  o First exposure should be medically supervised  o Rare cross-reactivity may occur in highly sensitive patients    5. Multimodal Analgesia Strategy (Guideline-Based)  Step 1 – Baseline therapy  • Paracetamol scheduled dosing  • Local anesthetic techniques  Step 2 – If insufficient pain control  • Add weak opioid (tramadol or codeine)  Step 3 – Severe pain  • Short-term strong opioid (morphine/oxycodone)  Step 4 – Avoid escalation pitfalls  • Do not introduce NSAIDs at any stage in confirmed AERD    6. Perioperative Risk Optimization  A. Preoperative assessment  • Confirm NSAID sensitivity history  • Evaluate asthma control status  • Review previous anaphylactoid/bronchospastic episodes  B. Asthma stability optimization  • Ensure:  o Inhaled corticosteroid compliance  o β2-agonist availability (salbutamol)  • Consider perioperative bronchodilator use in moderate/severe asthma    C. Emergency preparedness  In case of inadvertent exposure or bronchospasm:  • Inhaled β2-agonists (first-line)  • Systemic corticosteroids if needed  • Oxygen supplementation    7. Key Clinical Pitfalls  • “Mild NSAID use” is not safe in AERD  • Cross-reactivity is dose-independent and mechanism-based  • Opioids are safe from bronchospasm perspective but must be balanced against respiratory depression risk  • COX-2 inhibitors are not universally safe, but often tolerated    In patients with asthma and a history of aspirin- or NSAID-induced bronchospasm (AERD), postoperative pain management should be based on strict avoidance of all non-selective NSAIDs due to COX-1–mediated leukotriene overproduction and risk of severe bronchospasm. The first-line analgesic is paracetamol, administered in scheduled doses. Regional and local anesthetic techniques should be maximized to reduce systemic analgesic requirements. If additional pain control is needed, weak opioids such as tramadol or codeine may be used, followed by short-term strong opioids in cases of severe pain. COX-2 selective inhibitors such as celecoxib may be considered cautiously under supervision but are not first-line agents. This multimodal, opioid-sparing strategy minimizes respiratory risk while ensuring adequate analgesia. | Based on contemporary pharmacological and anesthesiology guidelines, the management of postoperative pain in an asthmatic patient with a history of aspirin or NSAID-induced bronchospasm requires strict avoidance protocols and an understanding of the underlying biochemical pathophysiology.  This clinical presentation is highly indicative of Aspirin-Exacerbated Respiratory Disease (AERD), historically known as Samter’s Triad (when accompanied by nasal polyposis). The condition is not an IgE-mediated true allergy, but rather a profound pharmacologic hypersensitivity.  1. Pathophysiologic Rationale and Absolute Contraindications  In patients with AERD, the administration of any drug that inhibits the Cyclooxygenase-1 (COX-1) enzyme blocks the synthesis of protective prostaglandins. Consequently, the arachidonic acid cascade is aggressively shunted down the 5-lipoxygenase (5-LOX) pathway. This results in a massive overproduction of cysteinyl leukotrienes (LTC4, LTD4, and LTE4), leading to acute, severe, and potentially fatal bronchoconstriction, mucosal edema, and mucus hypersecretion.  • Strictly Contraindicated: All non-selective NSAIDs (e.g., Ibuprofen, Naproxen, Diclofenac, Ketorolac) and Aspirin must be absolutely avoided.  2. First-Line Systemic Analgesia  • Acetaminophen (Paracetamol): This is the foundational, first-line analgesic for patients with AERD. Because it functions primarily via central mechanisms and has negligible peripheral COX-1 inhibitory effects at standard doses, it is safe for the vast majority of these patients.  • Clinical Protocol: Prescribe standard weight-based dosing, typically 500 mg to 1,000 mg every 6 hours, not exceeding 4,000 mg per day in a healthy adult. (Note: In very rare, highly sensitive AERD phenotypes, doses of Acetaminophen exceeding 1,000 mg in a single dose have been known to cause mild bronchospasm, so the lowest effective dose should be utilized).  3. Second-Line Escalation: Opioid Analgesics  If surgical trauma necessitates step-up therapy for breakthrough pain, opioids are utilized, but specific agents must be carefully selected based on their histamine-releasing profiles.  • Agents to Avoid (Natural Opiates): Morphine and Codeine should be avoided. These agents are notorious for triggering direct mast cell degranulation and non-immunologic histamine release, which can independently precipitate bronchospasm in hyperreactive asthmatic airways.  • Preferred Agents (Synthetic/Semi-Synthetic): Oxycodone, Hydrocodone, or Tramadol are preferred because they possess a significantly lower propensity to induce histamine release. They should be utilized at the lowest effective dose for 1 to 3 days postoperatively.  4. The COX-2 Inhibitor Consideration (Specialist Level)  Because AERD is strictly mediated by COX-1 inhibition, highly selective COX-2 inhibitors (e.g., Celecoxib) do not shunt the arachidonic acid pathway and are generally well-tolerated by these patients.  • Clinical Caveat: While current evidence supports the safety of Celecoxib in AERD, due to the severe consequences of a potential cross-reaction, a COX-2 inhibitor should typically only be prescribed if the patient has previously tolerated it, or under the direct guidance/clearance of the patient’s pulmonologist or allergist.  5. Perioperative Adjuvant Strategy (The OMFS Advantage)  To minimize the need for systemic analgesics, the surgical plan should heavily incorporate pre-emptive and locoregional techniques that simultaneously benefit airway reactivity:  • Locoregional Blockade: Utilization of a long-acting amide local anesthetic, such as Bupivacaine 0.5% with 1:200,000 epinephrine, to provide 8–12 hours of post-surgical anesthesia.  • Perioperative Corticosteroids: Intravenous or submucosal administration of a potent glucocorticoid (e.g., 8 mg Dexamethasone) prior to surgical incision. This not only profoundly suppresses the maxillofacial surgical inflammatory response (reducing edema and pain) but also systemically stabilizes the patient's reactive airway disease during the perioperative period. | Aspirin-Exacerbated Respiratory Disease (AERD), also known as Samter's Triad (asthma, chronic rhinosinusitis with nasal polyps, and NSAID/aspirin hypersensitivity), affects approximately 10–20% of adult asthmatics. The pathophysiological mechanism involves COX-1 inhibition, diverting arachidonic acid toward the 5-lipoxygenase (5-LOX) pathway, producing cysteinyl leukotrienes (LTC4, LTD4, LTE4) that trigger severe bronchospasm, rhinorrhoea, and urticaria. All non-selective COX inhibitors are absolutely contraindicated in these patients.  ABSOLUTELY CONTRAINDICATED ANALGESICS:  • Aspirin, Ibuprofen, Naproxen, Diclofenac, Ketorolac, Indomethacin, Meloxicam (at analgesic doses), Ketoprofen — any agent with significant COX-1 inhibitory activity.  SAFE ANALGESIC OPTIONS:  • 1. Paracetamol (Acetaminophen) — DRUG OF CHOICE: Does not inhibit COX-1/COX-2 peripherally to a significant extent. Safe at therapeutic doses (≤1000 mg per dose). IMPORTANT: A small subset of severely AERD-affected patients may react to high-dose paracetamol (>1000 mg single dose) due to weak COX-1 inhibitory activity. An initial test dose of 500 mg with observation for 30–60 minutes is advisable for patients with severe or unstable asthma.  • 2. Selective COX-2 Inhibitors (Celecoxib 200 mg BD, Etoricoxib 60–90 mg OD) — GENERALLY SAFE: Selective COX-2 inhibition does not divert arachidonic acid to the 5-LOX pathway (as COX-1 in mast cells is unaffected). Multiple clinical studies and cross-challenge protocols confirm safety in AERD patients. RECOMMENDATION: First challenge dose should be administered under medical supervision with bronchodilator availability. Celecoxib or etoricoxib are appropriate second-line analgesics when paracetamol alone is insufficient.  • 3. Tramadol 50–100 mg every 6–8 hours: No COX inhibition; safe in AERD. Weak mu-opioid agonist and serotonin-norepinephrine reuptake inhibitor. Appropriate for moderate post-surgical pain.  • 4. Opioids (Short-course): Codeine, oxycodone, or morphine may be used for severe post-operative pain. Note: codeine and morphine may trigger non-immunological histamine release from mast cells, which can exacerbate bronchospasm in susceptible individuals — use with caution; oxycodone is preferred.  • 5. Corticosteroids — Dexamethasone 4–8 mg IM/IV (perioperative): Dual benefit of postoperative oedema reduction and anti-inflammatory protection against asthma exacerbation. Highly recommended as a perioperative adjunct.  PERIOPERATIVE PRECAUTIONS:  • Ensure optimal asthma control (GINA Step >2) before elective surgery — procedure should be postponed if FEV1 <80% predicted or recent exacerbation.  • Short-acting beta-2 agonist (salbutamol MDI) must be immediately available throughout the procedure.  • Avoid NSAIDs in all postoperative prescriptions, including topical diclofenac gels, aspirin-containing compounds, and COX-1-inhibiting mouthwashes.  • Consult respiratory medicine if the patient has poorly controlled or brittle asthma. |
| 9. **Provide a detailed medical prescription protocol for infection prophylaxis and promoting sinus membrane healing following a maxillary molar extraction complicated by an oroantral communication?** | Medical Prescription Protocol After Maxillary Molar Extraction Complicated by Oroantral Communication (OAC)  (Infection Prophylaxis + Maxillary Sinus Membrane Healing Support – Evidence-Based OMFS Approach)    Clinical Context  An oroantral communication (OAC) following maxillary molar extraction creates an open pathway between the oral cavity and maxillary sinus, predisposing to:  • Acute or chronic maxillary sinusitis  • Contamination of sinus mucosa with oral flora  • Failure of spontaneous closure if >2–3 mm or persistent epithelialization  Management goals:  1. Prevent ascending sinus infection  2. Support sinus mucosal healing  3. Promote stable soft tissue closure  4. Reduce pressure changes across the defect    1. Antibiotic Prophylaxis / Infection Control  First-line regimen (non-allergic patients)  Amoxicillin–clavulanic acid  • Broad coverage of:  o Streptococci  o Anaerobes (oral + sinus flora)  o β-lactamase–producing organisms  📌 Rationale:  Odontogenic sinus infections are polymicrobial; β-lactamase coverage is essential.    Alternative (penicillin allergy)  Option 1  • Clindamycin  o Good anaerobic + Gram-positive coverage  o Widely used in odontogenic sinus communication cases  Option 2 (in clindamycin resistance or intolerance)  • Azithromycin  o Better safety profile  o Moderate oral flora coverage (often combined with metronidazole if severe infection risk)    Duration  • 5–7 days prophylactic course  • Extend to 10–14 days if sinusitis develops or OAC is large/persistent    2. Nasal and Sinus Decongestion Therapy (Critical Component)  Topical nasal decongestants  • Xylometazoline or oxymetazoline nasal spray  o Short course: 3–5 days maximum  o Reduces sinus ostium edema  o Promotes aeration and drainage    Systemic decongestants (optional)  • Pseudoephedrine  o Improves sinus ventilation  o Reduces mucosal edema    Saline irrigation  • Isotonic saline nasal irrigation (0.9%)  o Promotes mucociliary clearance  o Reduces bacterial load    3. Anti-inflammatory Therapy  Corticosteroids (adjunctive use)  • Intranasal corticosteroids (preferred)  o Fluticasone or mometasone  o Reduces sinus mucosal inflammation  📌 In selected cases:  • Short systemic corticosteroid course may be considered if:  o Significant sinus inflammation  o Persistent OAC-related edema    4. Analgesic Strategy  First-line  • Paracetamol (acetaminophen)  o Safe and effective baseline analgesic  Second-line (if needed)  • NSAIDs (ibuprofen) only if no contraindications  o Short-term use for inflammatory pain  o Caution due to bleeding and sinus mucosal effects  Severe pain  • Short-course weak opioids (tramadol/codeine)    5. Adjunctive Measures (Essential for Healing)  A. Pressure avoidance instructions  • No nose blowing  • No sneezing with closed mouth (sneeze with mouth open)  • Avoid smoking (critical factor in OAC failure)  • Avoid straw use or suction pressure    B. Oral hygiene  • Chlorhexidine 0.12–0.2% mouth rinse  o Twice daily  o Reduces oral bacterial load at communication site    C. Mechanical protection  • Sutured closure if possible (primary closure preferred)  • If delayed closure:  o Buccal advancement flap or buccal fat pad may be indicated    6. Healing Enhancement Strategy (Biological Rationale)  OAC healing depends on:  • Stable blood clot formation  • Sinus pressure equilibrium  • Absence of contamination  Thus pharmacologic support focuses on:  • Infection suppression (antibiotics)  • Mucosal edema reduction (decongestants + steroids)  • Reduced mechanical stress (behavioral instructions)    7. Clinical Red Flags (Require Escalation)  • Persistent nasal regurgitation >7–10 days  • Purulent nasal discharge  • Facial swelling or sinus tenderness  • Fever or systemic symptoms  ➡ Suggests acute maxillary sinusitis or failed closure → ENT/OMFS intervention required    8. High-Yield Prescription Summary  Standard regimen (typical case)  • Amoxicillin–clavulanic acid for 5–7 days  • Xylometazoline nasal spray for 3–5 days  • Saline nasal irrigation daily  • Paracetamol for pain control  • Chlorhexidine mouth rinse twice daily  Penicillin allergy  • Clindamycin OR azithromycin ± metronidazole  • Same adjunctive sinus and analgesic protocol    9. Concise Exam-Oriented Answer  Following maxillary molar extraction complicated by oroantral communication, infection prophylaxis should be based on broad-spectrum antibiotics targeting oral anaerobic and aerobic flora, with amoxicillin–clavulanic acid as first-line therapy and clindamycin or azithromycin as alternatives in penicillin-allergic patients. Adjunctive management includes short-term topical nasal decongestants, saline nasal irrigation, and intranasal corticosteroids to enhance sinus drainage and reduce mucosal inflammation. Patients should be instructed to avoid activities that increase sinus pressure such as nose blowing or sneezing with a closed mouth. Analgesia should be provided with paracetamol as first-line therapy, with short-term NSAIDs or opioids used if required. These measures collectively aim to prevent maxillary sinusitis and promote spontaneous or surgically assisted closure of the oroantral defect. | Based on contemporary oral and maxillofacial surgery (OMFS) guidelines and otolaryngology consensus statements regarding odontogenic sinusitis, the management of a newly created oroantral communication (OAC) following a maxillary molar extraction focuses on two primary objectives:  1. Prevention of secondary infection (acute maxillary sinusitis), which will inevitably compromise the blood clot and inhibit primary closure.  2. Promotion of unimpeded membrane healing by establishing a permissive anatomical environment (reducing sinus pressure and mucosal edema).  The following medical prescription protocol is standard for an OAC smaller than 5mm (where primary surgical closure or advancement flaps may be deferred pending spontaneous healing) or as the adjunctive medical regimen following immediate surgical closure (e.g., buccal fat pad or buccal advancement flap).  I. Infection Prophylaxis (Systemic Antibiotic Therapy)  The maxillary sinus harbors a distinct microbiome, and when breached by the oral cavity, empirical coverage must target both typical odontogenic flora (streptococci and anaerobes) and common sinonasal pathogens (e.g., Haemophilus influenzae, Streptococcus pneumoniae, Moraxella catarrhalis).  • First-Line Regimen (Beta-Lactamase Coverage):  o Amoxicillin-Clavulanate (Augmentin)  o Dose: 875 mg / 125 mg orally, every 12 hours (BID).  o Duration: 7 to 10 days.  o Clinical Rationale: The addition of clavulanic acid is critical. Amoxicillin alone is frequently insufficient because a significant percentage of H. influenzae and M. catarrhalis strains in the sinus are beta-lactamase producers.  • Alternative Regimen (For Penicillin-Allergic Patients):  o Mild/Non-IgE Mediated Allergy: Cefuroxime axetil (500 mg PO BID for 7–10 days).  o Severe/IgE Mediated Allergy: Moxifloxacin (400 mg PO daily for 7 days) or Clindamycin (300 mg PO QID for 7–10 days; though Clindamycin lacks robust H. influenzae coverage and is typically a second choice if a fluoroquinolone cannot be used).  II. Promoting Sinus Drainage and Reducing Edema  A patent ostiomeatal complex is the single most critical factor for sinus healing. If the maxillary ostium becomes occluded by mucosal edema, the sinus cannot drain, leading to stagnant secretions, infection, and rupture of the fragile OAC repair.  • 1. Topical Nasal Decongestant (Short-Term):  o Oxymetazoline Hydrochloride 0.05% Nasal Spray (e.g., Afrin)  o Dose: 2 sprays into the affected nostril, every 12 hours (BID).  o Duration: Strictly limited to 3 days.  o Clinical Rationale: Provides rapid, potent vasoconstriction of the nasal mucosa, immediately opening the ostiomeatal complex. Usage beyond 3–4 days is strictly contraindicated due to the high risk of rhinitis medicamentosa (rebound congestion).  • 2. Systemic Decongestant (If not medically contraindicated):  o Pseudoephedrine  o Dose: 60 mg orally, every 4 to 6 hours (or 120 mg extended-release every 12 hours).  o Duration: 5 to 7 days.  o Clinical Rationale: Provides sustained reduction in mucosal edema. (Contraindications: Severe hypertension, severe coronary artery disease, narrow-angle glaucoma, and concurrent MAOI use).  • 3. Intranasal Corticosteroid (Optional but Highly Recommended):  o Fluticasone Propionate 50 mcg/spray (e.g., Flonase)  o Dose: 2 sprays into each nostril, once daily.  o Duration: Begin on day 4 (following cessation of oxymetazoline) and continue for 10 to 14 days.  o Clinical Rationale: Suppresses the local inflammatory cascade, preventing long-term mucosal hypertrophy and ensuring continued ostial patency.  III. Patient Instructions (Sinus Precautions)  The pharmacological protocol is useless if mechanical pressure dislodges the blood clot or surgical repair. The patient must be explicitly instructed in writing to adhere strictly to the following "Sinus Precautions" for 10 to 14 days:  • No nose blowing: Any nasal discharge must be gently wiped, never blown.  • Sneeze with the mouth wide open: This dissipates the aerodynamic pressure gradient away from the maxillary sinus.  • No playing wind instruments or inflating balloons.  • No drinking through a straw: The negative intraoral pressure will dislodge the coagulum from the socket into the oral cavity.  • Avoid heavy lifting or strenuous exercise: This prevents sudden increases in systemic venous and sinus pressure.  • Avoid air travel or scuba diving: Severe barometric pressure changes can rupture the healing membrane. | An oroantral communication (OAC) represents a pathological connection between the oral cavity and the maxillary sinus following extraction of an upper molar or premolar. Clinical classification: small (<2 mm, may close spontaneously), medium (2–6 mm), large (>6 mm, requires surgical closure). Regardless of size, all OACs mandate immediate management to prevent ascending infection, chronic oroantral fistula formation, and iatrogenic maxillary sinusitis.  ANTIBIOTIC THERAPY:  • First-line: Amoxicillin-clavulanate 875/125 mg orally twice daily for 7–10 days. Rationale: provides coverage for oral flora (streptococci, anaerobes) combined with cover for sinus pathogens including Haemophilus influenzae and Moraxella catarrhalis (clavulanate-spectrum organisms). This dual coverage is superior to amoxicillin monotherapy for preventing post-extraction maxillary sinusitis.  • Penicillin allergy: Moxifloxacin 400 mg orally once daily for 7–10 days (covers oral flora, sinus pathogens, and anaerobes). Alternative: Metronidazole 400 mg three times daily + Ciprofloxacin 500 mg twice daily.  • If sinusitis is already established (purulent rhinorrhoea, facial pain/pressure, anosmia): extend antibiotic course to 14–21 days; ENT specialist referral mandatory.  NASAL DECONGESTANT:  • Xylometazoline 0.1% nasal spray: 1–2 sprays per nostril twice to three times daily, maximum 5–7 days (beyond which rhinitis medicamentosa risk). Objective: promote sinus drainage via ostiomeatal complex patency and reduce antral mucosal oedema.  MUCOLYTIC THERAPY:  • N-Acetylcysteine 600 mg orally once daily for 10 days, OR Ambroxol 30 mg three times daily: facilitates sinus drainage by reducing sputum viscosity.  ANTI-INFLAMMATORY/ANALGESIC:  • Ibuprofen 400 mg three times daily with food (if no contraindications) for 5 days, or Paracetamol 1000 mg four times daily. Objective: reduce sinus mucosal oedema and postoperative pain.  • Dexamethasone 4 mg IM single perioperative dose (if not already administered): reduces acute mucosal oedema and facilitates early sinus recovery.  ORAL ANTISEPTIC:  • Chlorhexidine gluconate 0.12% oral rinse twice daily — passive rinsing only (no forceful gargling or spitting). Do not irrigate the socket under pressure.  PATIENT INSTRUCTIONS — ANTRAL PRECAUTIONS (mandatory):  • Strict prohibition of nasal blowing for minimum 14 days.  • Sneezing should be performed with the mouth open to prevent intrasinus pressure rise.  • Avoid straws, smoking, and activities creating negative/positive intraoral pressure.  • Sleep in semi-reclined position to facilitate sinus drainage.  SURGICAL CLOSURE:  OAC ≥6 mm or persistent OAC >48 hours: proceed to surgical closure (buccal fat pad transposition, buccal advancement flap, or palatal rotation flap) under antibiotic cover. A Caldwell-Luc procedure or functional endoscopic sinus surgery (FESS) may be required if concomitant chronic sinusitis is established. |
| 10. **Calculate the appropriate dosage of an amoxicillin-clavulanate suspension for a pediatric patient weighing 25 kg diagnosed with a severe odontogenic abscess?** | Pediatric Dose Calculation: Amoxicillin–Clavulanate (25 kg Child with Severe Odontogenic Abscess)  Clinical Principle  In severe odontogenic infections in children, dosing is based on the amoxicillin component, using high-dose regimens (80–90 mg/kg/day) to ensure adequate serum and tissue levels against mixed aerobic–anaerobic oral flora.    1. Step 1 – Determine Total Daily Dose  Weight-based calculation (high-dose regimen)  Recommended: 80 mg/kg/day (amoxicillin component)  Patient weight: 25 kg  80×25=2000" mg/day amoxicillin"  ✔ Total daily dose = 2000 mg/day    2. Step 2 – Divide Daily Dose  Severe odontogenic abscess → typically twice daily (q12h) regimen for optimal compliance and pharmacodynamics.  2000÷2=1000" mg per dose"  ✔ Single dose = 1000 mg amoxicillin every 12 hours    3. Step 3 – Convert to Suspension Volume  Common formulation used:  Amoxicillin–clavulanate 400 mg / 57 mg per 5 mL  Amoxicillin concentration:  400" mg"/5" mL"=80" mg/mL"  Required volume per dose:  1000÷80=12.5" mL"  ✔ Final dose: 12.5 mL per administration    4. Final Prescription Scheme  Amoxicillin–clavulanate suspension (400/57 mg per 5 mL)  12.5 mL orally every 12 hours  Duration: typically 5–7 days, reassess clinically after 48–72 hours    5. Clinical Notes (Important in OMFS Practice)  A. Rationale for high-dose therapy  Severe odontogenic abscess = high bacterial load + anaerobic dominance  Ensures adequate penetration into:  Fascial spaces  Suppurative bone/inflammatory tissue    B. Clavulanate safety consideration  Total clavulanic acid exposure remains within pediatric safety limits when using standard formulations  If GI intolerance occurs:  Consider dose adjustment or alternative β-lactam regimen    C. Clinical monitoring  Re-evaluate within 48–72 hours  Escalate if:  Persistent swelling  Fever  Trismus or spreading infection    6. Concise Exam Answer  For a 25 kg pediatric patient with a severe odontogenic abscess, high-dose amoxicillin–clavulanate therapy is calculated at 80 mg/kg/day of the amoxicillin component. This corresponds to a total daily dose of 2000 mg, divided into two doses of 1000 mg every 12 hours. Using a suspension containing 400 mg/5 mL, each 1000 mg dose corresponds to 12.5 mL administered twice daily. | The pharmacological management of a severe odontogenic abscess in a pediatric patient demands an aggressive, bactericidal dosage regimen that effectively penetrates the suppurative fascial spaces while targeting beta-lactamase-producing organisms (e.g., Prevotella spp., Fusobacterium spp., and resistant Haemophilus influenzae).  For a severe infection, contemporary infectious disease and pediatric pharmacotherapy guidelines recommend utilizing a dosing parameter at the higher end of the standard therapeutic spectrum: 40 to 45 mg/kg/day of the amoxicillin component, administered in divided doses every 12 hours (BID).  (Note: In geographic regions with a high prevalence of resistant Streptococcus pneumoniae, or for exceptionally refractory cases, a "High-Dose" regimen of 80 to 90 mg/kg/day utilizing a specialized formulation with a lower clavulanate ratio, such as the 600/42.9 mg per 5 mL ES suspension, may be indicated to prevent clavulanic acid-induced gastrointestinal toxicity. However, the 45 mg/kg/day protocol is the standard initial empirical choice).  Here is the precise clinical calculation utilizing the widely available 400 mg / 57 mg per 5 mL suspension for a patient weighing 25 kg:  1. Daily Dosage Calculation  • Target Dosage: 45 mg/kg/day of the amoxicillin component.  • Patient Weight: 25 kg  • Total Daily Dose: 25 kg × 45 mg = 1,125 mg per day  2. Dosing Interval Calculation  • To ensure compliance and optimize the pharmacokinetic time-above-MIC (Minimum Inhibitory Concentration), this formulation is divided every 12 hours (BID).  • Single Dose Requirement: 1,125 mg / 2 = 562.5 mg per administration  3. Suspension Volume Calculation  • Utilizing the Amoxicillin-Clavulanate 400 mg / 5 mL suspension formulation:  • Equation: (Desired Dose / Concentration) × Volume  • Calculation: (562.5 mg / 400 mg) × 5 mL = 7.03 mL  4. The Formal Medical Prescription  In a clinical setting, calculating down to the hundredth of a milliliter is impractical for home administration. The dose is safely rounded to the nearest measurable interval.  • Rx: Amoxicillin-Clavulanate (Augmentin) Suspension 400 mg / 5 mL  • Dispense: 100 mL bottle (Quantity sufficient for a 7-day course: 7 mL × 2 × 7 = 98 mL)  • Sig: Administer 7.0 mL orally every 12 hours for 7 days. Take with meals to enhance absorption and minimize gastrointestinal distress.  Clinical Surgical Reminder: Systemic antibiotic therapy is strictly an adjunct in the presence of a true fluctuant abscess. The definitive treatment for a severe odontogenic abscess remains prompt surgical source control via incision and drainage (I&D) and extraction or endodontic extirpation of the offending tooth. | Dosage calculation for amoxicillin-clavulanate in severe paediatric odontogenic infection is guided by BNFC, AAP, and AAOMS paediatric guidelines. The dosing is expressed based on the amoxicillin component.  DOSING PROTOCOL — SEVERE INFECTION:  • Recommended dose for severe infection: 40–45 mg/kg/day of amoxicillin component, divided every 8 hours (TID).  • Standard available suspension: Amoxicillin/Clavulanate 250 mg/62.5 mg per 5 mL (250/62.5 suspension).  CALCULATION:  • Total daily dose (amoxicillin): 25 kg × 45 mg/kg/day = 1,125 mg/day  • Per dose (TID): 1,125 mg ÷ 3 = 375 mg per dose  • Volume per dose (using 250 mg/5 mL suspension): 375 mg ÷ 250 mg × 5 mL = 7.5 mL per dose  • PRESCRIPTION: Amoxicillin-Clavulanate oral suspension 250/62.5 mg per 5 mL — 7.5 mL (= 375 mg amoxicillin / 93.75 mg clavulanate) orally every 8 hours for 7–10 days.  • CLAVULANATE CHECK: Clavulanate per dose: 93.75 mg — Total daily: 281.25 mg → approximately 11.25 mg/kg/day. Slightly above the guideline maximum of 10 mg/kg/day for clavulanate (to minimise GI adverse effects — diarrhoea, nausea). Consider ES formulation.  HIGH-DOSE ES FORMULATION (preferred for severe infection):  • ES formulation (600/42.9 mg per 5 mL — 'Extended-Release/Extra-Strength'): delivers higher amoxicillin with lower relative clavulanate.  • Dose: 90 mg/kg/day (amoxicillin) ÷ 2 = 45 mg/kg/dose. 25 kg × 90 / 600 × 5 mL = 18.75 mL/day ÷ 2 doses = 9.4 mL every 12 hours.  • Clavulanate per day: 25 kg × 6.4 mg/kg = 160 mg/day — well within safety limits.  • If truly severe/hospitalised: IV ampicillin-sulbactam 200 mg/kg/day divided every 6 hours provides superior tissue penetration and should be considered for deep space infections or inability to take oral medications.  Duration: 7–10 days. Never exceed adult maximum dose (875/125 mg BID or 500/125 mg TID). Administer at the START of a meal to reduce GI adverse effects. |
| 11. **Discuss how the duration and dosage of postoperative antibiotic prophylaxis should be modified for patients receiving immunosuppressive therapy (e.g., methotrexate) for rheumatoid arthritis?** | Postoperative Antibiotic Prophylaxis in Patients on Immunosuppressive Therapy (e.g., Methotrexate for Rheumatoid Arthritis)  Clinical Context  Patients receiving disease-modifying antirheumatic drugs (DMARDs) such as methotrexate have:  • Impaired neutrophil function and cellular immunity  • Reduced inflammatory response (masking early infection signs)  • Delayed wound healing in some cases  • Potential additive myelosuppression when combined with certain antibiotics  However, current rheumatology and surgical guidelines generally agree that low-dose methotrexate (≤25 mg/week) is not an indication for routine alteration of standard antibiotic prophylaxis in minor oral surgery, unless additional risk factors exist.    1. Core Principle  Antibiotic prophylaxis is not automatically prolonged solely due to methotrexate use  Modern evidence-based guidance emphasizes:  • No routine extension of prophylactic antibiotics in stable RA patients on methotrexate alone  • Emphasis on clinical risk stratification rather than drug-based extension    2. Risk Stratification Approach  Antibiotic duration and dosing should be adjusted based on overall infection risk, not immunosuppressive therapy alone:  Low–Moderate risk (standard methotrexate monotherapy, well-controlled RA)  • Continue standard single-dose or short-course prophylaxis  • No extension solely due to methotrexate  High risk (modifying factors present)  Dose/duration adjustment is considered if methotrexate is combined with:  • Corticosteroids (e.g., ≥10 mg/day prednisone equivalent)  • Biologic agents (TNF-α inhibitors, IL-6 inhibitors)  • Poor glycemic control or diabetes  • Malnutrition or elderly patients  • Extensive bone surgery or infection presence    3. Antibiotic Prophylaxis Strategy  A. Standard regimen (no modification needed in most methotrexate patients)  For clean-contaminated oral surgery:  • Single preoperative dose (preferred in guidelines)  o Amoxicillin 2 g orally 30–60 min preoperatively  o OR clindamycin 600 mg if penicillin-allergic  📌 Key principle:  Methotrexate alone does NOT justify routine postoperative antibiotic continuation.    B. When postoperative antibiotics ARE indicated (high-risk cases)  Recommended modification:  • Short postoperative course only (not prolonged prophylaxis)  Typical regimens:  • Amoxicillin–clavulanate 500/125 mg every 8 hours for 3–5 days  • OR clindamycin 300 mg every 6–8 hours for 3–5 days  📌 Important:  • Avoid extending beyond 5 days unless active infection is present    4. Why Prolonged Antibiotics Should Be Avoided  A. No evidence of benefit  • Extended prophylaxis does not reduce surgical site infection rates in immunosuppressed RA patients undergoing routine OMFS procedures  B. Increased risk of harm  • Antibiotic resistance  • Clostridioides difficile infection (especially with clindamycin)  • Drug–drug interactions  C. Methotrexate interaction risk  Certain antibiotics increase methotrexate toxicity:  • Penicillins (reduce renal clearance of MTX)  • Trimethoprim-sulfamethoxazole (absolute contraindication due to marrow suppression risk)    5. Key Drug Interaction Considerations  Methotrexate + antibiotics  • Penicillins: may increase methotrexate serum levels → monitor if prolonged use  • Sulfonamides (TMP-SMX): strictly contraindicated  o Risk: pancytopenia, severe bone marrow suppression  • Macrolides: minimal direct interaction but caution in polypharmacy    6. Practical Clinical Algorithm  Step 1: Assess immunosuppression severity  • Methotrexate monotherapy → no change  • Combined immunosuppression → consider extension  Step 2: Choose prophylaxis strategy  • Standard: single pre-op dose  • High-risk: short 3–5 day postoperative course  Step 3: Avoid unnecessary prolongation  • No routine 7–10 day “protective antibiotics” without infection  Step 4: Monitor closely  • Early follow-up (48–72 h)  • Watch for:  o Delayed healing  o Subtle infection signs (often masked)    7. Evidence-Based Summary  In patients receiving methotrexate for rheumatoid arthritis, postoperative antibiotic prophylaxis should generally follow standard protocols without routine extension. Methotrexate monotherapy does not independently justify prolonged antibiotic courses. A single preoperative dose is sufficient for most procedures. In high-risk surgical scenarios or when combined immunosuppressive therapy is present, a short postoperative course of 3–5 days may be considered. Prolonged antibiotic prophylaxis (>5 days) is not recommended due to lack of evidence of benefit and increased risks of resistance, adverse effects, and drug interactions. Particular attention should be paid to avoiding trimethoprim-sulfamethoxazole due to severe toxicity risk with methotrexate.    High-Yield Exam Conclusion  Methotrexate use alone does not require modification of standard antibiotic prophylaxis in oral surgery. Antibiotics should not be prolonged unless additional immunosuppressive factors or clinical infection are present. When indicated, only short-duration postoperative regimens (3–5 days) are justified, with strict avoidance of interacting drugs such as trimethoprim-sulfamethoxazole. | Based on current evidence-based literature, guidelines from the American Association of Oral and Maxillofacial Surgeons (AAOMS), and cross-disciplinary consensus with the American College of Rheumatology (ACR), the management of a patient on Disease-Modifying Antirheumatic Drugs (DMARDs) such as methotrexate requires a targeted modification of the standard antibiotic prophylaxis protocol.  For a healthy, immunocompetent patient undergoing a "clean-contaminated" maxillofacial procedure (e.g., third molar extraction, standard implant placement), a single preoperative dose of a bactericidal antibiotic is considered sufficient, and postoperative antibiotics are generally contraindicated. However, for a patient on systemic methotrexate therapy, the protocol must be escalated to account for drug-induced immunosuppression and impaired mucosal healing.  1. Pathophysiologic Rationale for Modification  Methotrexate is a folate antimetabolite that competitively inhibits dihydrofolate reductase, thereby impairing DNA synthesis and cellular replication. In the context of oral and maxillofacial surgery, this translates to three critical surgical risks:  • Neutropenia/Leukopenia: Reduced quantitative and qualitative function of the innate immune system (macrophages and neutrophils), severely compromising the body's ability to sterilize the transient bacteremia induced by the surgical breach.  • Delayed Granulation: Impaired fibroblast proliferation delays the formation of a mature, protective blood clot and granulation tissue, leaving the osseous defect exposed to the polymicrobial oral environment for an extended period.  • Mucosal Friability: Methotrexate toxicity frequently manifests as stomatitis or oral ulcerations, providing a continuous portal of entry for opportunistic pathogens.  Current ACR guidelines strongly recommend continuing low-dose weekly methotrexate during the perioperative period to prevent a severe flare of rheumatoid arthritis. Because the immunosuppressive agent remains active, the surgical site must be protected via extended antimicrobial coverage.  2. Modification of the Antimicrobial Protocol  A. Preoperative Loading (Mandatory) Unlike healthy patients where preoperative antibiotics might be discretionary based on the exact procedure, an immunosuppressed patient must receive a high-dose preoperative loading dose to achieve peak bactericidal plasma and tissue concentrations at the exact moment of surgical incision.  • Standard: 2g Amoxicillin PO 30–60 minutes prior to surgery.  • Allergic (Non-IgE): 2g Cephalexin PO 30–60 minutes prior.  • Allergic (IgE-Mediated): 500 mg Azithromycin or 600 mg Clindamycin PO. (Note: Clindamycin is used with increasing caution due to C. difficile risks).  B. Postoperative Duration (The Primary Modification) While the standard dosage per administration remains therapeutic, the duration of the regimen is significantly prolonged. The goal is to provide systemic coverage until the primary mucosal seal is established and the underlying hematoma organizes.  • Extended Course: A postoperative antibiotic course of 5 to 7 days is strictly indicated following invasive dentoalveolar surgery (e.g., osseous impactions, bone grafting, multiple extractions).  • Preferred Agents: Broad-spectrum, bactericidal agents are preferred over bacteriostatic agents.  o First-Line: Amoxicillin 500 mg PO every 8 hours, OR Amoxicillin-Clavulanate 875/125 mg PO every 12 hours (to provide coverage against beta-lactamase producing organisms if the patient has a history of recurrent infections or recent hospitalizations).  3. Pre-Surgical Laboratory Stratification  The clinical decision to modify the antibiotic regimen must be correlated with objective laboratory data obtained within days of the planned surgery.  • Complete Blood Count (CBC) with Differential: The surgeon must evaluate the Absolute Neutrophil Count (ANC) and total White Blood Cell (WBC) count.  • Thresholds: If the WBC is < 4,000 cells/µL or the ANC is < 2,000 cells/µL, the patient is at a significantly elevated risk for a surgical site infection. In such cases, the 7-day postoperative prophylactic course is absolutely mandatory, and consultation with the treating rheumatologist is required to determine if additional localized measures (e.g., strict utilization of 0.12% chlorhexidine gluconate rinses BID) or a temporary adjustment of the DMARD dosing schedule is warranted. | Methotrexate (MTX), used at low weekly doses (7.5–25 mg/week) for rheumatoid arthritis, exerts its anti-inflammatory effect primarily through inhibition of dihydrofolate reductase and adenosine-mediated immunomodulation. Although less profoundly immunosuppressive than calcineurin inhibitors or high-dose biologics, MTX impairs neutrophil chemotaxis, T-lymphocyte proliferation, and mucosal immune surveillance, increasing susceptibility to surgical site infections.  ANTIBIOTIC PROPHYLAXIS MODIFICATIONS:  • INDICATION: Prophylactic antibiotics are indicated for all invasive oral surgical procedures (extractions, implant surgery, osteotomies, biopsy) in patients on MTX — this contrasts with the selective approach applied to healthy, immunocompetent individuals.  • DURATION EXTENSION: Extend postoperative antibiotic course to 5–7 days (compared to a standard 3-day course or single preoperative dose in immunocompetent patients). Duration should be reassessed if wound healing is delayed or signs of infection develop.  • DOSAGE: Standard adult dosages are generally maintained — no dose reduction required. Amoxicillin-clavulanate 875/125 mg twice daily for 5–7 days; or Amoxicillin 500 mg three times daily if polymicrobial coverage is not required.  • PREOPERATIVE CBC: A full blood count must be obtained to exclude MTX-induced pancytopenia (leukopenia, neutropenia). If absolute neutrophil count <1,000/mm³, the surgical procedure should be postponed and haematology/rheumatology consulted. If count is 1,000–1,500/mm³, broad-spectrum prophylaxis with amoxicillin-clavulanate is strongly recommended.  CRITICAL DRUG INTERACTIONS:  • MTX + NSAIDs: NSAIDs (including ibuprofen and diclofenac) reduce renal tubular secretion of MTX, leading to elevated plasma MTX concentrations and potential toxicity (mucositis, pancytopenia, hepatotoxicity). This is a clinically significant, potentially life-threatening interaction. STRICTLY AVOID NSAIDs in patients on MTX; use paracetamol as the sole analgesic.  • MTX + Cotrimoxazole (Trimethoprim-Sulfamethoxazole): Competitive inhibition of dihydrofolate reductase → profound MTX toxicity. Absolute contraindication.  • MTX + Tetracyclines: Modest displacement from plasma protein binding; use with caution.  MTX DRUG HOLIDAY — SURGICAL CONSIDERATIONS:  For major maxillofacial surgery (orthognathic surgery, tumour resections), a MTX drug holiday of 1 week pre- and 1 week postoperatively may be considered in consultation with the rheumatologist — balanced against the risk of disease flare. For routine dentoalveolar procedures, interruption of MTX is generally NOT recommended by current rheumatology guidelines (ACR 2022), as it does not significantly reduce infection risk and may precipitate arthritis relapse. |
| 12. **Evaluate the clinical scenarios in which a single preoperative dose of antibiotic prophylaxis is considered superior to a prolonged postoperative course in preventing surgical site infections in maxillofacial surgery?** | Single Preoperative Dose vs Prolonged Postoperative Antibiotics in Maxillofacial Surgery: Evidence-Based Evaluation of Clinical Scenarios  Core Principle (Modern Guideline Consensus)  Current international surgical and antimicrobial stewardship guidelines (including AAOMS-aligned principles and general surgical infection prevention standards) consistently emphasize:  A single appropriately timed preoperative antibiotic dose is equivalent—or superior—to prolonged postoperative antibiotic regimens in preventing surgical site infections (SSIs) in most clean and clean-contaminated maxillofacial procedures.  This superiority is not microbiological alone, but based on:  • Pharmacodynamics (time above MIC during incision and early wound phase)  • Host immune response timing  • Reduction of antimicrobial resistance and adverse events    1. Scientific Rationale for Preoperative Single-Dose Superiority  A. Pharmacodynamic principle: “Time of exposure matters most”  • SSI risk is highest during:  o Incision  o Early bacterial contamination phase  • Antibiotic effectiveness depends on achieving therapeutic tissue concentration at the time of incision  ✔ A single preoperative dose ensures:  • Peak tissue concentration during surgical contamination window    B. Lack of benefit from prolonged dosing  Multiple meta-analyses in surgical disciplines show:  • No significant reduction in SSI rates with postoperative continuation beyond 24 hours in clean-contaminated surgery  • In OMFS specifically, prolonged antibiotics do not reduce infection rates in routine third molar or orthognathic surgery    C. Harm of prolonged antibiotics  • Increased antimicrobial resistance  • Higher incidence of:  o Clostridioides difficile infection  o Drug hypersensitivity reactions  o Dysbiosis  • Increased cost and poor stewardship compliance    2. Clinical Scenarios Where Single Preoperative Dose is Preferred or Superior  A. Impacted third molar surgery (healthy patients)  Evidence:  • Routine postoperative antibiotics provide no additional SSI reduction  ✔ Indicated approach:  • Single preoperative dose (e.g., amoxicillin 2 g)  📌 Clinical rationale:  • Low infection risk baseline  • Short surgical duration  • Good vascularity of oral tissues    B. Routine dentoalveolar surgery (non-infected cases)  Examples:  • Simple extractions  • Minor alveoloplasty  • Implant placement in healthy bone  ✔ Preferred strategy:  • Preoperative prophylaxis only (if indicated at all)    C. Orthognathic surgery (uncomplicated cases)  • Evidence supports:  o Preoperative + intraoperative coverage  o Postoperative extension beyond 24 hours shows no SSI benefit in uncomplicated cases  ✔ Trend:  • Shift toward ≤24-hour total antibiotic exposure    D. Clean-contaminated maxillofacial trauma (stable, early managed cases)  • When:  o Early fixation achieved  o No gross contamination  o No devitalized tissue burden  ✔ Single-dose or ≤24-hour prophylaxis is sufficient    E. Dental implant surgery in healthy patients  • High evidence consistency:  o Single preoperative dose reduces early implant failure slightly  o Postoperative continuation does not improve outcomes    3. Situations Where Prolonged Postoperative Antibiotics Are NOT Superior (Even Harmful)  A. Healthy immunocompetent patients  • No benefit beyond perioperative window  • Increases resistance and adverse events    B. Clean surgical fields  • No active infection  • Good soft tissue closure    C. Short-duration procedures  • <2 hours surgery  • Minimal bone manipulation    4. Situations Where Prolonged Therapy MAY Still Be Justified (Not “superior”)  Important distinction:  These are exceptions, not evidence of superiority over single-dose prophylaxis.  A. Established active infection  • Odontogenic abscess  • Cellulitis  • Osteomyelitis  ✔ Here antibiotics are therapeutic, not prophylactic    B. Severe immunocompromised states  • Neutropenia  • High-dose chemotherapy  • Advanced uncontrolled diabetes  • Solid organ transplant (early phase)  ✔ Even here:  • Benefit is individualized, not universally prolonged prophylaxis    C. Grossly contaminated trauma  • Open fractures with delayed debridement  • Extensive soft tissue contamination    5. Key Evidence-Based Conclusion  The consistent evidence-based hierarchy is:  Best strategy (most cases in OMFS):  Single preoperative dose > prolonged postoperative antibiotics  Because it:  • Maximizes tissue concentration during contamination phase  • Minimizes resistance and adverse events  • Provides equal SSI protection in most elective maxillofacial surgeries    6. Clinical Decision Algorithm (Practical OMFS Use)  Step 1: Assess procedure type  • Clean / clean-contaminated → single dose  • Infected → therapeutic antibiotics (not prophylaxis)  Step 2: Assess patient risk  • Healthy → single dose only  • High-risk systemic condition → consider short perioperative course (≤24–72h)  Step 3: Avoid unnecessary extension  • Do NOT extend antibiotics beyond 24 hours unless active infection exists    7. High-Yield Summary (Exam-Oriented)  A single preoperative antibiotic dose is considered superior or at least equivalent to prolonged postoperative antibiotic regimens in preventing surgical site infections in most maxillofacial surgical procedures, including third molar surgery, orthognathic surgery, implant placement, and uncomplicated trauma repair. This approach ensures optimal drug concentration during the critical contamination window while minimizing antimicrobial resistance, adverse drug reactions, and dysbiosis. Prolonged postoperative antibiotic administration does not confer additional protective benefit in these scenarios and is generally discouraged under modern antimicrobial stewardship principles. Exceptions are limited to cases of active infection or severely immunocompromised patients, where antibiotics are therapeutic rather than prophylactic. | Based on contemporary antimicrobial stewardship principles and the most recent parameters of care established by the American Association of Oral and Maxillofacial Surgeons (AAOMS) and international surgical infectious disease societies, the paradigm of antibiotic prophylaxis in maxillofacial surgery has definitively shifted.  In the vast majority of elective, "clean-contaminated" maxillofacial procedures in immunocompetent hosts, a single preoperative dose of a targeted bactericidal antibiotic is demonstrably superior to prolonged postoperative courses for the prevention of Surgical Site Infections (SSIs).  Here is a technical evaluation of the pharmacokinetic rationale and the specific clinical scenarios where this protocol is the standard of care.  The Pharmacokinetic and Microbiological Rationale  The superiority of the single preoperative dose is rooted in fundamental pharmacokinetics. To prevent an SSI, the antimicrobial agent must be present in the host's serum and target tissues at bactericidal concentrations at the precise moment the mucosal or cutaneous barrier is breached.  Administering an intravenous or oral dose 30 to 60 minutes prior to the surgical incision ensures peak tissue saturation during the period of maximum bacterial inoculation. Conversely, administering antibiotics after the procedure (postoperative courses) fails to prevent the initial bacterial adhesion and colonization of the surgical hematoma. Once a coagulum forms and bacteria begin synthesizing a biofilm, systemic antibiotics rapidly lose their efficacy.  Clinical Scenarios Dictating Single Preoperative Prophylaxis  1. Orthognathic Surgery Procedures such as the Bilateral Sagittal Split Osteotomy (BSSO) and Le Fort I osteotomy are classified as clean-contaminated due to the necessity of transoral incisions to access the facial skeleton.  • Evidence: Extensive systematic reviews demonstrate that a single preoperative intravenous dose of a first-generation cephalosporin (e.g., Cefazolin 2g) or Penicillin G provides equivalent or superior SSI reduction compared to a 5-day postoperative course.  • Protocol: The dose may be re-administered intraoperatively if the surgery exceeds the drug's half-life (e.g., >3-4 hours) or if there is massive blood loss (>1,500 mL), but it should be discontinued within 24 hours postoperatively.  2. Routine and Complex Dentoalveolar Surgery (Including Impactions) The prophylactic use of antibiotics for the surgical extraction of asymptomatic, uninfected impacted third molars is heavily scrutinized.  • Evidence: Cochrane systematic reviews indicate that the Number Needed to Treat (NNT) to prevent a single minor localized infection is exceptionally high (often >12 to 24 patients).  • Protocol: If the surgeon deems prophylaxis necessary due to anticipated extreme surgical difficulty or extensive osteotomy, a single preoperative dose of Amoxicillin (2g) or an appropriate alternative is strictly superior. Postoperative prescribing in healthy patients offers no statistically significant reduction in alveolar osteitis or SSI, but drastically increases the incidence of adverse drug events.  3. Uncomplicated Dental Implantology and Minor Bone Grafting The placement of endosseous titanium implants, with or without minor localized Guided Bone Regeneration (GBR), does not warrant prolonged postoperative antibiotics.  • Evidence: The current consensus confirms that a single preoperative dose of Amoxicillin (2g PO 1 hour prior to surgery) significantly reduces the rate of early implant failure.  • Protocol: Extending this coverage into a 5- or 7-day postoperative regimen provides zero additional benefit for osseointegration or SSI prevention and is strongly discouraged.  4. Maxillofacial Traumatology (Non-Comminuted, Non-Contaminated Fractures) For mandibular or midface fractures communicating with the oral cavity (e.g., fractures through the tooth-bearing segments).  • Evidence: While antibiotic therapy is often initiated at the time of presentation (conceptually "perioperative" rather than purely prophylactic), guidelines indicate that extending antibiotic therapy beyond 24 hours post-Open Reduction and Internal Fixation (ORIF) does not decrease hardware infection rates.  • Protocol: A preoperative loading dose prior to fixation is critical; prolonged postoperative courses are obsolete unless the wound is grossly contaminated, comminuted, or involves avascular necrosis.  The Deleterious Effects of Prolonged Postoperative Courses  To establish superiority, one must evaluate the risk-to-benefit ratio. Prolonged postoperative antibiotic courses are not merely "ineffective" in these scenarios; they are actively harmful. The single preoperative dose is superior because it mitigates the following iatrogenic complications:  • Antimicrobial Resistance (AMR): Sub-lethal, prolonged exposure to broad-spectrum antibiotics is the primary driver for the selection of multi-drug-resistant organisms within the oral microbiome.  • Adverse Drug Events (ADEs): Prolonged courses exponentially increase the risk of hypersensitivity reactions, severe gastrointestinal toxicity, and opportunistic fungal overgrowth (e.g., oral or vulvovaginal candidiasis).  • Clostridioides difficile Colitis: Extended use of antibiotics, particularly clindamycin or broad-spectrum penicillins, disrupts the gut microbiome, posing a severe and potentially life-threatening risk of C. difficile infection—a risk that is practically eliminated with single-dose preoperative prophylaxis. | Emerging evidence from multiple randomised controlled trials and systematic reviews supports the principle that a single appropriately timed preoperative antibiotic dose achieves equivalent surgical site infection (SSI) prevention compared to prolonged postoperative courses for the majority of elective maxillofacial surgical procedures, while conferring significant advantages in antimicrobial stewardship.  EVIDENCE BASE:  Kaczmarzyk et al. (2007): RCT demonstrating no statistically significant difference in SSI rates between a single preoperative amoxicillin dose and a 5-day postoperative course following third molar surgery. Lacasa et al. (2007): Multicentre RCT confirming equivalence for third molar procedures. Peterson et al. and Murphy et al.: Support single-dose protocols for orthognathic surgery. SIGN Guideline 44 and AAOMS guidelines endorse antimicrobial stewardship principles consistent with single-dose prophylaxis.  CLINICAL SCENARIOS WHERE SINGLE PREOPERATIVE DOSE IS SUPERIOR/PREFERRED:  • 1. Third Molar (Impacted Wisdom Tooth) Surgery in immunocompetent patients: Level Ia evidence supports a single preoperative dose of amoxicillin 2 g (or amoxicillin-clavulanate 2 g) 30–60 minutes before incision. Equivalent infection prevention with reduced resistance, C. difficile risk, and adverse effects.  • 2. Orthognathic Surgery (BSSO, Le Fort I, Genioplasty): Single IV perioperative dose (amoxicillin-clavulanate 2.2 g or cefazolin 2 g) at induction. High-quality RCT evidence supports this protocol; prolonged courses confer no additional protection in healthy patients.  • 3. Dental Implant Placement: Although controversy exists, current evidence (Esposito et al., Cochrane 2013) favours a single preoperative dose of amoxicillin 2 g or 3 g over extended postoperative courses for implant failure prevention. Some protocols add a single postoperative dose, but there is no evidence for multi-day courses.  • 4. Temporomandibular Joint Arthroscopy/Arthroplasty: Clean operative field; single perioperative IV dose is standard.  • 5. Simple Facial Fractures (Closed, Non-Contaminated): Single perioperative dose at time of ORIF. Evidence does not support extended courses for isolated zygomatic or orbital fractures.  ADVANTAGES OF SINGLE PREOPERATIVE DOSE:  • Antimicrobial stewardship: reduction in selective pressure for resistant organisms.  • Significantly reduced Clostridioides difficile-associated diarrhoea risk.  • Lower incidence of adverse drug reactions (rash, GI intolerance, drug interactions).  • Superior patient compliance (single dose vs. multi-day course).  • Cost-effectiveness.  SCENARIOS WHERE PROLONGED COURSES REMAIN INDICATED:  • Compound mandibular fractures with intraoral communication (contaminated wound field): 3–5-day postoperative course. Pre-existing infection (MRONJ, osteomyelitis, deep space infections): therapeutic rather than prophylactic antibiotics. Immunocompromised patients (organ transplants, HIV, methotrexate, chemotherapy): extended 5–7-day courses. Panfacial fractures with delayed surgical management: contaminated field warrants extended cover.  TIMING: The single preoperative dose must be administered 30–60 minutes before surgical incision to achieve adequate tissue drug levels at the time of contamination — the window of maximum prophylactic efficacy. |
| 13. **What is the standard protocol for infective endocarditis prophylaxis prior to an invasive dental procedure in a patient with a prosthetic heart valve and no known allergies?**   **○ A) 2g Amoxicillin administered 30-60 minutes before the procedure**  **○ B) 1g Amoxicillin administered 1 hour after the procedure**  **○ C) 600 mg Clindamycin administered 30 minutes before the procedure**  **○ D) 2g Cephalexin administered immediately before the procedure**  **○ E) 500 mg Azithromycin administered 2 hours before the procedure** | The correct answer is A) 2g Amoxicillin administered 30–60 minutes before the procedure.  According to the 2021 American Heart Association (AHA) Scientific Statement on the Prevention of Viridans Group Streptococcal Infective Endocarditis, patients with a prosthetic cardiac valve (mechanical or bioprosthetic) are classified as high-risk individuals requiring antibiotic prophylaxis prior to invasive dental procedures. In the absence of any known drug allergy — specifically penicillin allergy — the first-line recommended regimen is a single oral dose of Amoxicillin 2g administered 30 to 60 minutes before the dental procedure.  This regimen provides adequate serum and oral tissue levels during the period of bacteraemia associated with the invasive procedure. The single-dose approach is preferred over multi-dose regimens to minimise antibiotic exposure and reduce the risk of adverse effects and antimicrobial resistance.  Why the other options are incorrect:  — Option B (1g, post-procedure): Prophylaxis must be administered pre-procedurally; post-procedural administration does not prevent the bacteraemia that occurs during the procedure. The dose of 1g is also insufficient.  — Option C (600mg Clindamycin): The 2021 AHA guidelines explicitly removed clindamycin from the list of recommended alternatives due to the increased risk of Clostridioides difficile infection. Clindamycin is no longer recommended for IE prophylaxis.  — Option D (2g Cephalexin immediately before): Although cephalexin 2g is a valid alternative for penicillin-allergic patients without anaphylaxis history, it should be administered 30–60 minutes before, and it is not the first-line choice when the patient has no allergy.  — Option E (500mg Azithromycin): Azithromycin/clarithromycin are alternative agents for patients who cannot tolerate oral beta-lactams, not first-line agents. | The correct answer is A) 2g Amoxicillin administered 30–60 minutes before the procedure.  The 2021 American Heart Association (AHA) updated Scientific Statement on Prevention of Viridans Group Streptococcal Infective Endocarditis establishes the current standard of care for IE prophylaxis in high-risk cardiac patients undergoing invasive dental procedures. Patients with prosthetic cardiac valves — whether mechanical or bioprosthetic — represent the highest-risk category for IE and are among those for whom prophylaxis provides the most clinical benefit.  For a patient with no known drug allergies, the recommended regimen is:  • Amoxicillin 2g orally (adults), administered 30–60 minutes before the invasive dental procedure  • A single-dose strategy is employed to minimise unnecessary antibiotic exposure  Mechanistically, amoxicillin achieves bactericidal serum concentrations against viridans group streptococci — the primary organisms implicated in IE following oral bacteraemia — well above the minimum inhibitory concentration (MIC) for the duration of the at-risk period.  Option B is incorrect because pre-procedural administration is essential; post-procedural prophylaxis is clinically ineffective and is not endorsed by any major guideline. Option C (clindamycin) was formally removed from the AHA 2021 recommendation list due to the unacceptable risk of C. difficile-associated diarrhoea. Option D (cephalexin) is reserved for penicillin-allergic patients without anaphylaxis history. Option E (azithromycin) is an alternative for those intolerant of oral beta-lactam agents. The 2021 AHA guidelines align with the 2023 ESC Endocarditis Guidelines in recommending amoxicillin as the undisputed first-line agent in non-allergic patients. | The correct answer is A) 2g Amoxicillin administered 30–60 minutes before the procedure.  Clinical Rationale and Guideline Foundation  The 2021 AHA Scientific Statement on the Prevention of Viridans Group Streptococcal Infective Endocarditis (Wilson et al., Circulation 2021;143:e963–e978) provides the definitive current evidence base for IE prophylaxis in high-risk patients. Patients with prosthetic cardiac valves are explicitly designated as the highest-priority cohort for prophylaxis, given the catastrophic consequences of prosthetic valve IE and the difficulty of treatment.  Recommended Regimen (No Allergy):  • Drug: Amoxicillin  • Dose: 2g (adults)  • Route: Oral  • Timing: 30–60 minutes before the invasive dental procedure  • Strategy: Single pre-procedural dose  Pharmacological Rationale:  Amoxicillin is a bactericidal aminopenicillin with excellent oral bioavailability (~90%), rapid gastrointestinal absorption achieving peak serum concentrations within 1–2 hours, and broad-spectrum activity against viridans streptococci — the predominant causative organisms in dental procedure-related IE. The 30–60 minute pre-procedural window ensures that therapeutic serum concentrations coincide with the period of maximum bacteraemia.  Systematic Elimination of Distractors:  — Option B: The temporal requirement for pre-procedural administration is pharmacologically essential. Post-procedural administration cannot prevent bacteraemia that has already occurred during the procedure.  — Option C (Clindamycin 600mg): Explicitly removed from the AHA 2021 recommendations due to epidemiological evidence demonstrating significantly elevated risk of severe C. difficile infection, including potentially fatal pseudomembranous colitis.  — Option D (Cephalexin 2g): A valid second-tier alternative for patients with non-severe (non-anaphylactic) penicillin allergy; not indicated when the patient has no allergy and amoxicillin is available.  — Option E (Azithromycin 500mg): Recommended only for patients who cannot tolerate oral beta-lactam agents; its bacteriostatic rather than bactericidal mechanism makes it a pharmacologically inferior choice when bactericidal agents are tolerated.  This question directly tests knowledge of a critical patient safety protocol with immediate clinical relevance in OMFS practice. |
| 14. **Which of the following analgesics is most frequently cited in the orthopedic and maxillofacial literature as having a potential negative impact on bone healing?**   **○ A) Paracetamol (Acetaminophen)**  **○ B) Ketorolac**  **○ C) Tramadol**  **○ D) Selective COX-2 inhibitors**  **○ E) Ibuprofen** | The correct answer is B) Ketorolac.  In the orthopedic and maxillofacial surgery literature, non-steroidal anti-inflammatory drugs (NSAIDs) — and particularly ketorolac — are most frequently cited as agents with a potential negative impact on bone healing. The mechanism involves inhibition of cyclooxygenase (COX) enzymes, which suppresses prostaglandin synthesis. Prostaglandins (particularly PGE2) play a critical role in osteoblast differentiation, bone formation, and the early inflammatory cascade that initiates fracture repair.  Ketorolac is singled out in the maxillofacial literature due to its high potency and common use in the perioperative period. Several clinical and animal studies have associated perioperative NSAID use — especially ketorolac — with impaired bone fusion and delayed fracture healing when administered during the critical early remodelling phase.  Option A (Paracetamol): Does not inhibit prostaglandin synthesis peripherally to a clinically significant degree and is not associated with impaired bone healing.  Option C (Tramadol): An opioid analgesic without relevant cyclooxygenase inhibitory activity; not associated with bone healing impairment.  Option D (Selective COX-2 inhibitors): Although COX-2 inhibitors theoretically carry some risk due to the role of COX-2 in bone remodelling, the evidence base is less robust than for non-selective NSAIDs like ketorolac in the OMFS and orthopaedic literature.  Option E (Ibuprofen): Also an NSAID with potential effects on bone healing, but ketorolac is more consistently cited in the surgical literature as the primary concern. | The correct answer is B) Ketorolac.  Among the available analgesic agents, ketorolac — a potent parenteral and oral non-selective NSAID — is most frequently cited in both the orthopaedic and maxillofacial surgery literature as having a significant potential negative impact on bone healing.  Mechanism of Impairment:  The prostaglandin-mediated inflammatory cascade is not merely a byproduct of bone injury but an essential driver of the healing process. Prostaglandin E2 (PGE2), produced via COX-1 and COX-2 enzymes, stimulates osteoprogenitor cell differentiation, promotes osteoblast activity, and facilitates early angiogenesis at the fracture site. Non-selective NSAIDs such as ketorolac inhibit both COX isoforms, thereby suppressing PGE2 production and attenuating this osteogenic cascade.  Clinical Evidence:  Multiple clinical and experimental studies have demonstrated that:  1. Perioperative ketorolac administration is associated with higher rates of non-union in orthopaedic fractures  2. Animal models consistently show delayed mineralisation and reduced bone density at osteotomy sites following NSAID exposure  3. The maxillofacial literature specifically identifies NSAID use during the first 72 hours post-osteotomy as potentially compromising osseointegration and healing at condylar and mandibular osteotomy sites  Comparative Analysis of Options:  — Paracetamol (A): Primarily acts centrally; peripheral COX inhibition is minimal and clinically insignificant for bone healing  — Tramadol (C): Opioid mechanism; no relevant interaction with prostaglandin-mediated bone metabolism  — COX-2 inhibitors (D): Theoretically implicated due to the role of COX-2 in late-phase remodelling, but evidence is less consistent than for non-selective NSAIDs  — Ibuprofen (E): Non-selective NSAID with similar mechanism to ketorolac but lower potency and less frequently cited as primary concern | The correct answer is B) Ketorolac.  Pharmacological Basis for Bone Healing Impairment  Bone healing is a complex, prostaglandin-dependent process that proceeds through three overlapping phases: inflammatory (days 1–7), reparative (weeks 2–8), and remodelling (months to years). The early inflammatory phase, far from being merely a pathological response, is essential for osteoprogenitor cell recruitment and differentiation. Prostaglandin E2 (PGE2), synthesised via cyclooxygenase (COX)-1 and COX-2 pathways, acts on EP2 and EP4 receptors on osteoblasts and mesenchymal stem cells, promoting osteoblastogenesis and suppressing osteoclastogenesis in the early healing phase.  Why Ketorolac:  Ketorolac tromethamine is a potent non-selective COX-1/COX-2 inhibitor with analgesic potency approximately 0.5× that of morphine. Its high systemic exposure and non-selective inhibition profile make it the NSAID most consistently identified in the orthopaedic and oral and maxillofacial surgery literature as associated with:  • Impaired fracture union and delayed healing  • Reduced mineralisation at osteotomy sites  • Compromised osseointegration in implant surgery  • Higher non-union rates at mandibular osteotomy sites in orthognathic surgery patients  A clinically important implication: ketorolac should be used with caution or avoided in the immediate postoperative period following osteotomy procedures, distraction osteogenesis, or bone graft placement in OMFS.  Option Differentiation:  — Paracetamol: Predominantly central COX inhibition; no clinically significant peripheral prostaglandin suppression; safe profile for bone healing  — Tramadol: Opioid + weak serotonin-noradrenaline reuptake inhibition; no COX-related bone healing interference  — COX-2 inhibitors: Theoretically implicated (COX-2 upregulation is part of the normal osteogenic response), but clinical evidence is less consistent; may be safer alternatives to non-selective NSAIDs  — Ibuprofen: Non-selective NSAID with similar mechanism; lower potency than ketorolac and less consistently cited as the primary concern in surgical literature |
| 15. **To avoid a "disulfiram-like reaction," patients must strictly abstain from alcohol while taking which of the following antibiotics?**   **○ A) Amoxicillin**  **○ B) Metronidazole**  **○ C) Azithromycin**  **○ D) Clindamycin**  **○ E) Doxycycline** | The correct answer is B) Metronidazole.  Metronidazole (and other nitroimidazole antibiotics such as tinidazole and ornidazole) are the agents that mandate strict alcohol abstinence due to the risk of a disulfiram-like reaction.  Mechanism:  Metronidazole inhibits acetaldehyde dehydrogenase (ALDH), the enzyme responsible for oxidising acetaldehyde — the primary toxic metabolite of ethanol — to acetic acid. When alcohol is consumed concurrently with metronidazole, acetaldehyde accumulates in the blood, producing a characteristic constellation of symptoms: facial flushing, throbbing headache, nausea, vomiting, hypotension, tachycardia, and diaphoresis. In severe cases, cardiac arrhythmias and cardiovascular collapse may occur.  Clinical Guidance:  Patients must be counselled to avoid all alcohol consumption — including alcohol-containing mouthwashes, cough syrups, and food preparations — during metronidazole therapy and for at least 48 hours following completion of the course.  Why the other options are incorrect:  — Option A (Amoxicillin): No acetaldehyde dehydrogenase inhibition; alcohol is not clinically contraindicated.  — Option C (Azithromycin): No interaction with alcohol metabolism via the ALDH pathway.  — Option D (Clindamycin): Hepatically metabolised but does not inhibit acetaldehyde dehydrogenase.  — Option E (Doxycycline): Some reports suggest alcohol may reduce doxycycline serum levels (via enzyme induction), but a true disulfiram-like reaction does not occur. | The correct answer is B) Metronidazole.  The disulfiram-like reaction is a well-documented and potentially serious drug-alcohol interaction that occurs specifically with nitroimidazole antibiotics, most notably metronidazole.  Biochemical Mechanism:  Ethanol is normally metabolised in two sequential enzymatic steps:  1. Ethanol → Acetaldehyde (via alcohol dehydrogenase)  2. Acetaldehyde → Acetate (via acetaldehyde dehydrogenase [ALDH])  Metronidazole inhibits ALDH, the enzyme responsible for step 2. When alcohol is ingested during metronidazole therapy, acetaldehyde accumulates in the bloodstream, producing the characteristic toxidrome:  • Cutaneous vasodilation and flushing  • Severe throbbing headache  • Nausea, vomiting  • Palpitations, tachycardia  • Hypotension and diaphoresis  • In severe cases: cardiovascular collapse, arrhythmia  The identical mechanism underlies the therapeutic use of disulfiram (Antabuse) in alcohol aversion therapy, hence the eponymous reaction name.  Clinical Significance in OMFS:  Metronidazole is widely used in OMFS for the management of odontogenic infections, pericoronitis, and periodontal disease due to its exceptional coverage of anaerobic bacteria. Patients must receive explicit counselling to abstain from all alcohol-containing products — including mouthwashes, sauces, desserts, and over-the-counter medications — during treatment and for 48 hours after the final dose.  The other antibiotics listed (amoxicillin, azithromycin, clindamycin, doxycycline) do not inhibit ALDH and do not produce a disulfiram-like reaction. | The correct answer is B) Metronidazole.  Mechanism of the Disulfiram-Like Reaction  The disulfiram-like reaction — named for its mechanistic similarity to the therapeutic alcohol-aversion drug disulfiram (Antabuse) — occurs specifically with nitroimidazole antibiotics: metronidazole, tinidazole, ornidazole, and secnidazole.  Pathway of Interaction:  Normal alcohol metabolism:  1. Ethanol → Acetaldehyde (alcohol dehydrogenase, ADH)  2. Acetaldehyde → Acetate (acetaldehyde dehydrogenase, ALDH) — BLOCKED by metronidazole  3. Acetate → CO₂ + H₂O  By competitively inhibiting ALDH (specifically ALDH-2 in hepatic mitochondria), metronidazole causes toxic acetaldehyde accumulation (blood acetaldehyde concentrations may reach 5–10× normal). The resulting syndrome is dose- and concentration-dependent:  Clinical Manifestations (in order of severity):  Mild: Flushing, warmth, tingling  Moderate: Pounding headache, nausea, vomiting, tachycardia, hypotension  Severe: Arrhythmias, cardiovascular collapse, seizures (rare)  Critical Patient Counselling Points:  • Abstain from all alcohol during metronidazole therapy AND for 48 hours after the last dose  • Includes: alcoholic beverages, mouthwashes containing ethanol, alcohol-based cooking preparations, and alcohol-containing medications (e.g., cough syrups)  • Topical metronidazole gel may also carry this risk if sufficient systemic absorption occurs  Why Other Options are Incorrect:  — Amoxicillin (A): Renal excretion via unchanged drug; no ALDH interaction  — Azithromycin (C): CYP3A4-metabolised; no ALDH inhibitory activity  — Clindamycin (D): Hepatically glucuronidated; no acetaldehyde dehydrogenase inhibition  — Doxycycline (E): Some literature suggests alcohol may reduce doxycycline blood levels (via hepatic enzyme induction), but there is NO disulfiram-like reaction  This interaction has direct clinical relevance in OMFS: metronidazole is among the most commonly prescribed antibiotics in the specialty, and many postoperative patients may consume alcohol without understanding the risk. |
| 16. **Which antibiotic group can increase the risk of statin toxicity and rhabdomyolysis in patients taking medications like atorvastatin?**   **○ A) Penicillins**  **○ B) Cephalosporins**  **○ C) Macrolides**  **○ D) Tetracyclines**  **○ E) Aminoglycosides** | The correct answer is C) Macrolides.  Macrolide antibiotics — including erythromycin, clarithromycin, and to a lesser extent azithromycin — are known inhibitors of the cytochrome P450 3A4 (CYP3A4) enzyme system. Most lipophilic statins, including atorvastatin, simvastatin, and lovastatin, undergo extensive first-pass and systemic metabolism via CYP3A4.  When a macrolide antibiotic is co-administered with a CYP3A4-metabolised statin, competitive inhibition of CYP3A4 results in significantly elevated statin plasma concentrations. This pharmacokinetic interaction increases the risk of statin-induced myopathy and, in severe cases, rhabdomyolysis — a life-threatening condition characterised by skeletal muscle breakdown with release of myoglobin, which can cause acute renal failure.  Clinicians prescribing macrolides in OMFS practice should screen patients for concurrent statin use. If macrolide therapy is necessary, consideration should be given to temporarily withholding the statin, switching to a non-CYP3A4-metabolised macrolide (e.g., azithromycin has weaker CYP3A4 inhibition compared to erythromycin or clarithromycin), or selecting an alternative antibiotic class. | The correct answer is C) Macrolides.  The drug-drug interaction between macrolide antibiotics and statins is a clinically significant pharmacokinetic interaction mediated through inhibition of the cytochrome P450 3A4 (CYP3A4) enzyme system.  Mechanism of Interaction:  Erythromycin and clarithromycin are potent CYP3A4 inhibitors. The majority of commonly prescribed statins — including atorvastatin, simvastatin, and lovastatin — rely on CYP3A4 for first-pass and systemic hepatic metabolism. When CYP3A4 is inhibited by a macrolide:  • Statin plasma concentrations increase substantially  • Myotoxic metabolite accumulation occurs  • Risk of myopathy and rhabdomyolysis increases significantly  Clinical Implications:  Rhabdomyolysis presents with severe muscle pain, weakness, and dark (tea-coloured) urine (myoglobinuria), and can progress to acute kidney injury and death. The risk is highest with:  — Erythromycin > Clarithromycin > Azithromycin (azithromycin has weaker CYP3A4 inhibition)  — Higher statin doses  — Elderly patients or those with pre-existing renal/hepatic impairment  Why Other Options are Incorrect:  — Penicillins (A): Not CYP3A4 inhibitors; no relevant statin interaction  — Cephalosporins (B): Similarly, no CYP450 inhibitory activity  — Tetracyclines (D): Not significantly CYP3A4 inhibiting  — Aminoglycosides (E): Renally cleared; no relevant CYP450 interactions | The correct answer is C) Macrolides.  Pharmacokinetic Basis of the Interaction  The macrolide-statin interaction is one of the most clinically important drug interactions encountered in OMFS practice, given the frequent use of clarithromycin and erythromycin in dental infections and the high prevalence of statin therapy in middle-aged and older patients.  CYP3A4 Inhibition Mechanism:  Erythromycin and clarithromycin are classified as strong CYP3A4 inhibitors. They competitively and irreversibly bind to the CYP3A4 isoenzyme, forming stable inhibitor-enzyme complexes that significantly reduce the hepatic and intestinal first-pass metabolism of co-administered drugs that are CYP3A4 substrates.  Statins Affected:  • Atorvastatin (Lipitor): CYP3A4-dependent; major interaction  • Simvastatin: CYP3A4-dependent; major interaction (highest risk)  • Lovastatin: CYP3A4-dependent; major interaction  • NOT affected: Pravastatin, rosuvastatin, fluvastatin (not CYP3A4 substrates)  Consequence: Statin-induced Myopathy and Rhabdomyolysis  Elevated plasma statin concentrations (up to 5–10× normal) lead to:  1. Myopathy: muscle pain, weakness, elevated CK  2. Rhabdomyolysis: massive muscle necrosis → myoglobinuria → acute tubular necrosis → renal failure  3. Mortality if untreated  Clinical Management Strategy in OMFS:  1. Review patient medication list pre-prescribing  2. If macrolide essential: consider temporary statin suspension for duration of therapy  3. Prefer azithromycin (weaker CYP3A4 inhibition) over erythromycin/clarithromycin when possible  4. Consider alternative antibiotics (clindamycin, amoxicillin-clavulanate) in statin-dependent patients  Comparative Elimination:  — Penicillins (A): Renally excreted without significant hepatic CYP metabolism  — Cephalosporins (B): No CYP3A4 inhibitory activity  — Tetracyclines (D): Mild CYP inhibition; no clinically significant statin interaction  — Aminoglycosides (E): Not orally absorbed; renally excreted; no CYP interactions  This interaction is directly relevant to the OMFS prescriber: clarithromycin is frequently used for odontogenic infections and sinusitis management, and the macrolide-statin interaction has resulted in reported deaths, making it a mandatory prescribing awareness point. |
| 17. **Which microorganism is the primary target for empirical antibiotic therapy in the treatment of acute suppurative parotitis?**   **○ A) Streptococcus mutans**  **○ B) Staphylococcus aureus**  **○ C) Actinomyces israelii**  **○ D) Porphyromonas gingivalis**  **○ E) Candida albicans** | The correct answer is B) Staphylococcus aureus.  Acute suppurative (bacterial) parotitis is a bacterial infection of the parotid gland characterised by the retrograde ascent of bacteria from the oral cavity into Stensen's duct. The condition is most commonly encountered in elderly, debilitated, dehydrated, or postoperative patients in whom salivary flow is reduced.  Staphylococcus aureus is the primary target for empirical antibiotic therapy in acute suppurative parotitis. It is the predominant causative organism, accounting for approximately 50–90% of cases, including methicillin-resistant S. aureus (MRSA) in hospitalised patients. The organism colonises the oropharynx and, in the context of reduced parotid salivary flow, ascends retrogradely through Stensen's duct into the parenchyma.  Clinical implications: Empirical therapy should provide anti-staphylococcal coverage. In the community setting, an anti-staphylococcal penicillin (e.g., dicloxacillin or nafcillin) or a first-generation cephalosporin is appropriate. In the hospital setting, MRSA coverage (vancomycin) should be considered.  The other organisms listed are not primary targets in suppurative parotitis: Streptococcus mutans is associated with dental caries; Actinomyces israelii causes actinomycosis; Porphyromonas gingivalis is a periodontal pathogen; Candida albicans causes fungal infections and is not a target for antibacterial therapy. | The correct answer is B) Staphylococcus aureus.  Acute suppurative parotitis is a bacterial infection of the parotid gland, classically arising from retrograde bacterial ascent through Stensen's duct under conditions of reduced salivary flow (dehydration, xerostomia, post-surgical state, anticholinergic medications).  Microbiology:  Staphylococcus aureus is the predominant organism, identified in approximately 50–80% of cases. It is considered the primary empirical target for the following reasons:  1. It is a predominant oral and skin coloniser with a propensity for glandular infection  2. It produces virulence factors (coagulase, protein A, toxins) that facilitate parenchymal destruction  3. MRSA strains are increasingly prevalent in nosocomial and healthcare-associated cases  Empirical Antibiotic Selection Based on Primary Target:  • Community-acquired: Anti-staphylococcal penicillin (e.g., dicloxacillin) or first-generation cephalosporin (e.g., cefazolin)  • Hospital-acquired / MRSA risk: Vancomycin or daptomycin  Systematic Elimination:  — Streptococcus mutans (A): Primary caries pathogen; not associated with parotid gland infection  — Actinomyces israelii (C): Causes chronic granulomatous cervicofacial actinomycosis; distinct clinical presentation  — Porphyromonas gingivalis (D): Key periodontal pathogen; not a primary cause of suppurative parotitis  — Candida albicans (E): Fungal pathogen; not treated with antibacterial agents; not a target in suppurative parotitis  Note: Culture and sensitivity testing of parotid pus (obtained via massage of Stensen's duct) is recommended to direct definitive therapy. | The correct answer is B) Staphylococcus aureus.  Clinical and Microbiological Context  Acute suppurative parotitis is a bacterial infection of the parotid gland characterised by purulent exudate expressible from Stensen's duct, acute painful parotid swelling, fever, trismus, and erythema overlying the gland. It predominantly affects debilitated, dehydrated, or immunocompromised patients, postoperative patients with reduced oral intake, elderly individuals, and those on xerogenic medications (anticholinergics, diuretics, antidepressants).  Pathophysiology:  The primary pathogenic mechanism is retrograde bacterial ascent through Stensen's duct, facilitated by:  • Reduced salivary flow (loss of antimicrobial flushing action)  • Salivary stasis and ductal obstruction (sialoliths, mucous plugs)  • Immunosuppression  Microbiology and Empirical Target:  Staphylococcus aureus is identified as the causative organism in 50–90% of cases across published case series and is the undisputed primary empirical target. Of clinical importance:  • Community-acquired cases: S. aureus (methicillin-sensitive, MSSA) predominates  • Hospital/healthcare-associated cases: MRSA prevalence is significant (30–40%)  • Polymicrobial infections involving streptococci, anaerobes, and Gram-negative organisms also occur  Evidence-Based Empirical Antibiotic Regimens:  Community setting: Dicloxacillin 500mg QID PO or cefazolin 1g TID IV  MRSA risk (hospitalised): Vancomycin 15–20mg/kg Q8–12h IV  Polymicrobial/anaerobic concern: Addition of metronidazole or use of amoxicillin-clavulanate  Adjunctive Management:  • Aggressive IV rehydration (cornerstone of treatment)  • Warm compresses, massage of Stensen's duct  • Sialogogues to stimulate salivary flow  • Surgical drainage if abscess formation occurs (computed tomography guidance)  Differentiation from Incorrect Options:  — Streptococcus mutans: Acidogenic cariogenic pathogen confined to dental biofilm; no documented role in suppurative parotitis  — Actinomyces israelii: Causes slowly progressive cervicofacial actinomycosis characterised by indurated swelling, sulfur granules, and sinus tract formation — a fundamentally different entity  — Porphyromonas gingivalis: Obligate anaerobic periodontal pathogen; not a primary parotid gland pathogen  — Candida albicans: Opportunistic fungal pathogen requiring antifungal (azole/echinocandin) rather than antibacterial therapy |
| 18. **Which group of antibiotics is strictly contraindicated during pregnancy due to the risk of fetal tooth discoloration and impaired bone development?**   **○ A) Cephalosporins**  **○ B) Macrolides**  **○ C) Penicillins**  **○ D) Tetracyclines**  **○ E) Lincosamides** | The correct answer is D) Tetracyclines.  Tetracyclines (including doxycycline, minocycline, and tetracycline) are strictly contraindicated throughout pregnancy — particularly after the 5th week of gestation — due to two well-established adverse effects on the developing fetus:  1. Tooth discolouration: Tetracyclines bind avidly to calcium ions and are incorporated into developing calcified tissues including fetal teeth and bone. This results in permanent yellow-brown to grey discolouration of primary (deciduous) teeth and, with exposure in later pregnancy, potentially permanent teeth.  2. Impaired bone development: Tetracyclines chelate calcium in developing osseous tissue, potentially inhibiting longitudinal bone growth and causing temporary growth retardation in the fetus.  These effects occur because tetracyclines cross the placental barrier freely and concentrate in calcifying tissues during organogenesis. For dental practice, the key clinical message is that tetracyclines should never be prescribed to pregnant patients.  The other antibiotics listed are generally considered safer in pregnancy:  — Cephalosporins (A) and Penicillins (C): Considered safe throughout pregnancy (FDA Category B); no teratogenic effects documented.  — Macrolides (B): Azithromycin and erythromycin are generally considered compatible with pregnancy (with the exception of erythromycin estolate, which carries hepatotoxicity risk); clarithromycin should be avoided.  — Lincosamides (E): Clindamycin is generally considered relatively safe in pregnancy when the benefit outweighs the risk. | The correct answer is D) Tetracyclines.  Tetracycline antibiotics — including doxycycline, minocycline, and tetracycline itself — are absolutely contraindicated during pregnancy (Category D) due to their unique pharmacokinetic affinity for calcified tissues.  Mechanism of Fetal Harm:  1. Dental Staining (Tetracycline Chelation):  Tetracyclines form stable calcium orthophosphate complexes by chelating Ca²⁺ ions. This complex is incorporated into the hydroxyapatite crystalline structure of developing dental enamel and dentine. The result is:  • Permanent intrinsic yellow-brown to dark grey-brown discolouration of primary teeth  • Fluorescence under ultraviolet light  • Potential enamel hypoplasia  The critical period of vulnerability extends from tooth bud development (approximately week 14 of gestation) through the first 8 years of postnatal life.  2. Impaired Bone Development:  The same calcium chelation mechanism causes tetracycline deposition in developing fetal bone, resulting in temporary growth inhibition. This effect is reversible after cessation of exposure in most cases.  3. Hepatotoxicity Risk:  High-dose intravenous tetracyclines carry additional risk of fatal hepatotoxicity in pregnant women.  Safe Alternatives in Pregnancy:  • Beta-lactams (penicillins, cephalosporins): Safe throughout pregnancy  • Clindamycin: Generally acceptable when indicated  • Azithromycin: Acceptable; use with caution  Options A, B, C, E are all comparatively safer choices in the pregnant patient. | The correct answer is D) Tetracyclines.  Regulatory Classification and Mechanism of Teratogenicity  Tetracycline antibiotics — including tetracycline, doxycycline, and minocycline — are classified as FDA Pregnancy Category D (previously), indicating positive evidence of human fetal risk. They are explicitly contraindicated for use in pregnant women and in children under 8 years of age.  Mechanism 1 — Intrinsic Dental Staining:  Tetracyclines have a unique physicochemical property: they are avid chelators of divalent cations, particularly calcium (Ca²⁺) and magnesium (Mg²⁺). This chelation affinity causes:  • Tetracycline incorporation into mineralising dental hard tissues (enamel matrix, dentine)  • Formation of stable tetracycline-calcium orthophosphate complexes in developing tooth structure  • Permanent intrinsic discolouration of primary teeth: yellow → brown → grey-brown progression  • Potential secondary discolouration of permanent teeth if exposure occurs in early postnatal life  Critical exposure window: approximately weeks 14–20 of gestation through 8 years postnatal  Mechanism 2 — Skeletal Development Impairment:  The same calcium chelation mechanism leads to tetracycline deposition in developing osseous tissue, causing:  • Temporary inhibition of longitudinal bone growth (generally reversible)  • Fluorescent banding of bone visible on histological examination  • Potential impacts on fetal bone density  Additional Maternal Risk:  High-dose intravenous tetracycline in pregnancy is associated with potentially fatal acute fatty liver disease (hepatic failure).  Safe Antibiotic Alternatives in Pregnancy for OMFS Practice:  • Amoxicillin / Amoxicillin-clavulanate (FDA Cat. B): First choice  • Cephalosporins (FDA Cat. B): Safe and effective  • Clindamycin: Acceptable when specifically indicated  • Azithromycin: Used with caution; generally considered acceptable  • Metronidazole: Avoid in first trimester; second/third trimester use with caution  Systematic Elimination of Distractors:  — Cephalosporins (A): FDA Category B; no documented teratogenic effects; safe throughout pregnancy  — Macrolides (B): Generally considered compatible (except clarithromycin and erythromycin estolate); no chelation of calcium  — Penicillins (C): The safest antibiotic class in pregnancy; extensively used; FDA Category B  — Lincosamides (E): Clindamycin is acceptable when clinically indicated; no known teratogenicity |
| 19. **For a patient with a history of gastrointestinal (GI) bleeding, which agent is considered the safest for managing postoperative pain while minimizing GI side effects?**   **○ A) Diclofenac sodium**  **○ B) Naproxen**  **○ C) Acetylsalicylic acid**  **○ D) Indomethacin**  **○ E) Celecoxib** | The correct answer is E) Celecoxib.  For a patient with a history of gastrointestinal bleeding, selective COX-2 inhibitors such as celecoxib are considered the safest class of NSAIDs for managing postoperative pain, as they minimise the gastrointestinal adverse effects associated with non-selective NSAIDs.  The gastric mucosa is protected primarily by prostaglandins synthesised via COX-1. Non-selective NSAIDs (diclofenac, naproxen, ibuprofen, aspirin, indomethacin) inhibit both COX-1 and COX-2, thereby reducing protective prostaglandin E2 and prostacyclin synthesis in the gastric mucosa, impairing the mucus-bicarbonate layer, and increasing vulnerability to peptic ulceration and haemorrhage.  Celecoxib selectively inhibits COX-2 — the inducible isoform primarily responsible for mediating pain and inflammation — while sparing COX-1-dependent gastroprotective mechanisms. This selectivity substantially reduces the risk of GI bleeding, ulceration, and erosion compared to non-selective NSAIDs.  Clinical note: Although celecoxib is safer than non-selective NSAIDs for the GI tract, it carries increased cardiovascular risk (as do all NSAIDs), which should be considered in patients with established cardiovascular disease.  All other options listed (diclofenac, naproxen, acetylsalicylic acid, indomethacin) are non-selective NSAIDs with significant GI toxicity, making them contraindicated or to be used with extreme caution in patients with a history of GI bleeding. | The correct answer is E) Celecoxib.  In patients with a documented history of gastrointestinal bleeding or peptic ulcer disease, careful analgesic selection is critical to preventing recurrent haemorrhage. Among the options provided, celecoxib — a selective COX-2 inhibitor — offers the most favourable gastrointestinal safety profile.  Pathophysiology of NSAID-Induced GI Injury:  The gastric mucosa is protected by prostaglandins (PGE2 and PGI2) generated via COX-1 enzymatic activity. These prostaglandins stimulate mucus secretion, bicarbonate production, and mucosal blood flow. Non-selective NSAID inhibition of COX-1 removes this cytoprotective effect, predisposing to:  • Gastric and duodenal erosions  • Peptic ulceration  • Upper GI haemorrhage (risk increased 3–5× compared to non-NSAID users)  Celecoxib's Mechanism of GI Safety:  Celecoxib preferentially inhibits the inducible COX-2 isoform, which mediates inflammation, pain, and fever, while substantially sparing COX-1-mediated gastroprotection. Multiple large clinical trials (CLASS, CONDOR) have confirmed significantly lower rates of GI adverse events with celecoxib compared to non-selective NSAIDs.  The remaining options — diclofenac, naproxen, acetylsalicylic acid, and indomethacin — are all non-selective COX inhibitors. Indomethacin carries the highest GI toxicity risk and is among the most ulcerogenic NSAIDs available. Aspirin permanently acetylates both COX isoforms and has the highest GI toxicity of the group. | The correct answer is E) Celecoxib.  Analgesic Selection in the GI-Compromised Patient: A Mechanistic Approach  The choice of analgesic in a patient with a history of gastrointestinal haemorrhage requires a detailed understanding of the COX enzyme system and its role in gastric mucosal defence.  COX Isoform Pharmacology:  • COX-1 (constitutive): Constitutively expressed throughout the GI tract; synthesises cytoprotective prostaglandins (PGE2, PGI2) that maintain gastric mucosal integrity via mucus secretion, bicarbonate production, epithelial restitution, and mucosal blood flow  • COX-2 (inducible): Upregulated at sites of inflammation, injury, and fever; primary mediator of pain and inflammatory prostaglandin synthesis  Why Celecoxib is the Correct Choice:  Celecoxib is a highly selective COX-2 inhibitor (COX-2:COX-1 selectivity ratio ~7.6). By selectively targeting COX-2 while sparing COX-1, celecoxib:  1. Delivers equivalent analgesic and anti-inflammatory efficacy to non-selective NSAIDs  2. Preserves COX-1-dependent gastroprotection  3. Reduces upper GI adverse events by 50–60% compared to non-selective NSAIDs (CLASS Trial data)  4. Avoids the platelet aggregation inhibition of non-selective NSAIDs (platelets express only COX-1)  Important Clinical Caveat: Celecoxib carries increased cardiovascular thrombotic risk (reduced prostacyclin without offsetting thromboxane inhibition), which must be balanced against GI benefit, particularly in patients with established cardiovascular disease.  Systematic Elimination:  — Diclofenac (A): Non-selective NSAID; despite relatively higher COX-2 selectivity than other traditional NSAIDs, still associated with significant GI toxicity and hepatotoxicity  — Naproxen (B): Non-selective NSAID; long half-life (12–17h) with prolonged mucosal exposure; high GI toxicity  — Acetylsalicylic acid (C): Irreversibly acetylates both COX-1 and COX-2; platelet COX-1 inhibition persists for platelet lifespan (8–10 days); extreme GI toxicity; absolutely contraindicated in GI bleeding history  — Indomethacin (D): Among the most potent and most GI-toxic NSAIDs available; structurally associated with the highest rates of peptic ulceration; contraindicated in GI-compromised patients |
| 20. **What is the most appropriate analgesic formulation for a trauma patient currently under maxillomandibular fixation (MMF)?**   **○ A) Enteric-coated tablet**  **○ B) Effervescent tablet or syrup**  **○ C) Sublingual tablet**  **○ D) Chewable tablet**  **○ E) Capsule** | The correct answer is B) Effervescent tablet or syrup.  A patient under maxillomandibular fixation (MMF) has their jaws immobilised — typically with arch bars, intermaxillary fixation screws, or elastic/wire fixation — which prevents the patient from opening the mouth, chewing, or swallowing solid or semi-solid oral dosage forms.  Effervescent tablets are dissolved in water prior to administration, producing a liquid solution that can be easily consumed through a straw, syringe, or around the fixation device without requiring mouth opening or mastication. Similarly, syrup formulations provide a liquid analgesic that can be administered without the need for jaw movement.  Why the other formulations are inappropriate under MMF:  — Option A (Enteric-coated tablet): A solid dosage form requiring swallowing intact; cannot be crushed without compromising the enteric coating; swallowing solid tablets is difficult without adequate jaw mobility.  — Option C (Sublingual tablet): While sublingual tablets dissolve under the tongue without swallowing, the immobilisation of the jaw under MMF may impair tongue placement and the ability to retain the tablet for adequate dissolution; not ideal.  — Option D (Chewable tablet): Absolutely contraindicated under MMF; requires active mastication.  — Option E (Capsule): Solid oral dosage form; requires swallowing intact; difficulty in MMF patients.  Clinical note: All medications for MMF patients should be liquid or dispersible formulations. Nutrition, hydration, and medication administration in these patients require a liquid-based regimen. | The correct answer is B) Effervescent tablet or syrup.  Maxillomandibular fixation (MMF) — whether achieved via arch bars, intermaxillary fixation (IMF) screws, or wire/elastic bands — immobilises the mandible in occlusion, rendering the patient unable to open the mouth for chewing, biting, or conventional oral medication ingestion.  Pharmaceutical Considerations in MMF Patients:  The primary constraints for medication administration in MMF patients are:  1. Inability to open the mouth sufficiently to place solid dosage forms on the tongue  2. Inability to chew  3. Potential difficulty swallowing due to limited mouth opening and jaw immobility  Rationale for Effervescent Tablets or Syrups:  Effervescent analgesic preparations (e.g., effervescent paracetamol, effervescent aspirin — if appropriate) dissolve completely in water to produce a homogeneous liquid solution that can be:  • Administered via a straw inserted past the fixation  • Given via oral syringe alongside the dental fixation  • Easily swallowed without requiring jaw opening or mastication  Liquid syrups (e.g., paracetamol suspension) offer equivalent flexibility.  Rejection of Alternatives:  — Enteric-coated tablets (A): Solid; cannot be crushed without destroying the coating; unsuitable  — Sublingual tablets (C): Practical limitations under MMF due to restricted tongue mobility and saliva accumulation  — Chewable tablets (D): Absolutely contraindicated; requires mastication  — Capsules (E): Require intact swallowing with adequate mouth opening | The correct answer is B) Effervescent tablet or syrup.  Clinical Context: Medication Administration in Maxillomandibular Fixation  Maxillomandibular fixation (MMF) is applied following mandibular fractures, orthognathic surgery (Le Fort osteotomy, bilateral sagittal split osteotomy), and certain TMJ procedures. It creates a stable, immobilised occlusal relationship by fixating the mandible to the maxilla using arch bars with wires or elastics, or intermaxillary fixation screws. The patient's ability to open the mouth is typically limited to 0–2mm or completely eliminated for the duration of fixation (typically 4–6 weeks for fractures).  Consequences for Oral Drug Administration:  Standard solid oral dosage forms are contraindicated or impractical:  • Tablets and capsules require adequate jaw opening and tongue placement  • Chewable formulations require active mastication — impossible under MMF  • Enteric-coated tablets cannot be crushed without destroying pH-sensitive coating  The Correct Solution — Liquid Formulations:  Effervescent tablets: Dissolved in a glass of water prior to administration, producing a clear liquid solution that can be:  1. Administered via a flexible straw inserted alongside the arch bar/IMF screw  2. Given using a 10–20mL oral syringe inserted at the commissure of the lip  3. Swallowed without any requirement for jaw opening  Syrups / Oral suspensions: Pre-formulated liquid analgesics (paracetamol syrup, ibuprofen suspension) offer equivalent accessibility.  Complete Medication Review Required:  Beyond analgesics, ALL medications must be reviewed and converted to liquid formulations in MMF patients:  • Antibiotics → liquid suspension or IV  • Analgesics → effervescent or syrup  • Nutritional supplementation → liquid enteral formula  Option Elimination:  — Enteric-coated (A): Solid; coating protects against gastric acid and cannot be removed  — Sublingual (C): While technically possible, practical difficulties under MMF (restricted sublingual space, reduced salivary clearance) limit reliability  — Chewable (D): Absolutely contraindicated — requires mastication  — Capsule (E): Solid oral form; requires adequate swallowing motion |
| 21. **When should a surgeon administer an NSAID to achieve preemptive analgesia for third molar surgery?**   **○ A) Immediately following the procedure**  **○ B) 30-60 minutes prior to the surgical incision**  **○ C) When the effect of the local anesthetic begins to subside**  **○ D) After the removal of sutures**  **○ E) Upon the first sensation of pain** | The correct answer is B) 30–60 minutes prior to the surgical incision.  Preemptive analgesia refers to the administration of analgesic agents before the onset of noxious stimuli (i.e., before surgical incision) with the goal of preventing central sensitisation — the neurological process by which repeated painful stimuli lead to enhanced pain perception (hyperalgesia and allodynia) in the postoperative period.  For NSAIDs administered orally, peak plasma concentrations are typically achieved 30–60 minutes after ingestion. Therefore, administering the NSAID 30–60 minutes before the surgical incision ensures that therapeutic plasma concentrations — and thus maximum COX inhibitory activity — are present at the tissue level at the time that surgical trauma and prostaglandin release occur. This attenuates the initial peripheral sensitisation of nociceptors and reduces the central sensitisation cascade.  This approach has been validated in multiple randomised controlled trials in third molar surgery and has been shown to:  — Reduce immediate postoperative pain intensity  — Decrease total analgesic consumption in the postoperative period  — Improve patient satisfaction  The other timing options are incorrect because:  — Option A (post-procedure): Analgesic is administered after nociceptive input has already occurred; no preemptive effect  — Option C/D/E (local anaesthetic subsides, suture removal, first pain): These represent reactive rather than preemptive pain management strategies | The correct answer is B) 30–60 minutes prior to the surgical incision.  Preemptive Analgesia: Conceptual and Pharmacological Foundation  Preemptive analgesia is defined as the administration of analgesic agents before surgical noxious stimuli to prevent the establishment of peripheral and central sensitisation — neural amplification processes that, once established, make postoperative pain more severe and difficult to treat.  Neurobiological Rationale:  Surgical tissue trauma triggers the arachidonic acid cascade via phospholipase A2, releasing prostaglandins (PGE2, PGI2) and other inflammatory mediators that directly sensitise peripheral nociceptors (peripheral sensitisation) and facilitate long-term potentiation of dorsal horn neurons in the spinal cord (central sensitisation). By pre-emptively inhibiting COX-mediated prostaglandin synthesis before tissue injury occurs, NSAIDs:  1. Attenuate peripheral nociceptor sensitisation  2. Reduce ascending nociceptive input to the CNS  3. Prevent or reduce the degree of central sensitisation  Timing Rationale:  Oral NSAIDs (ibuprofen, diclofenac, ketorolac) reach peak plasma concentrations (Cmax) and maximum tissue inhibitory levels approximately 30–60 minutes after ingestion. Administration 30–60 minutes pre-operatively therefore ensures that:  • Peak COX inhibitory concentrations coincide with the onset of surgical trauma  • Prostaglandin synthesis is maximally suppressed during the critical period of nociceptive barrage  Clinical Evidence:  Multiple RCTs in third molar surgery demonstrate that pre-operative NSAID administration significantly reduces:  • VAS pain scores in the first 6–24 hours postoperatively  • Rescue analgesic consumption  • Time to first analgesic request | The correct answer is B) 30–60 minutes prior to the surgical incision.  Preemptive Analgesia: Mechanism, Evidence, and Clinical Application  Definition and Neurobiological Basis:  Preemptive analgesia is an analgesic intervention initiated before the onset of surgical noxious stimulation with the specific aim of preventing the establishment of central sensitisation — a state of enhanced CNS excitability that amplifies postoperative pain and can contribute to the development of chronic post-surgical pain.  The molecular basis involves prevention of:  1. Peripheral sensitisation: Prostaglandins (PGE2, PGI2) and other eicosanoids directly lower the firing threshold of Aδ and C-fibre nociceptors at the surgical site. Pre-emptive COX inhibition reduces prostaglandin production before this sensitisation can occur.  2. Central sensitisation: Sustained peripheral nociceptive input leads to NMDA receptor activation, substance P release, and long-term potentiation of dorsal horn neurons. By reducing peripheral input preoperatively, the magnitude of central sensitisation is attenuated.  Pharmacokinetic Rationale for 30–60 Minute Pre-incision Timing:  • Oral NSAIDs: Peak plasma concentration (Tmax) typically 1–2 hours post-administration  • Onset of tissue-level COX inhibition: 30–45 minutes post-ingestion  • Therapeutic window alignment: 30–60 min pre-operative administration ensures maximal COX inhibitory activity at the moment of first surgical incision  Clinical Evidence (Third Molar Surgery):  The evidence base from third molar surgery RCTs consistently demonstrates:  • Preemptive NSAID administration reduces peak postoperative pain (VAS reduction 15–30%)  • Reduces total 24-hour analgesic consumption by 25–40%  • Extends time to first rescue analgesic request by 30–60 minutes  • No significant increase in intraoperative bleeding at therapeutic doses  Timing Options Eliminated:  — Immediately after (A): Nociceptive input has already been initiated; purely reactive  — Anaesthetic subsiding (C): Pain is already established; no preemptive benefit  — Suture removal (D): Analgesic administered hours after surgery; purely rescue analgesia  — First pain sensation (E): Central sensitisation has already begun; reactive only |
| 22. **Which of the following antibiotics has the lowest efficacy against the anaerobic bacteria commonly found in the oral cavity?**   **○ A) Metronidazole**  **○ B) Amoxicillin-Clavulanic Acid**  **○ C) Clindamycin**  **○ D) Azithromycin**  **○ E) Ciprofloxacin** | The correct answer is E) Ciprofloxacin.  Ciprofloxacin is a fluoroquinolone antibiotic with excellent activity against aerobic Gram-negative bacteria. However, it has notoriously poor efficacy against anaerobic bacteria — the predominant pathogens in odontogenic infections of the oral cavity.  The oral cavity harbours a complex polymicrobial flora dominated by anaerobic species, including Porphyromonas gingivalis, Prevotella intermedia, Fusobacterium nucleatum, Peptostreptococcus species, and Bacteroides species. These organisms are inherently resistant to fluoroquinolones because they lack the oxidative metabolism that fluoroquinolones partially depend on for their mechanism of action, and their reduced outer membrane permeability limits drug entry.  Comparative anaerobic coverage among the listed agents:  — Metronidazole (A): Excellent anaerobic coverage; considered the gold standard for oral anaerobes  — Amoxicillin-clavulanic acid (B): Excellent broad-spectrum coverage including oral anaerobes via beta-lactamase inhibition  — Clindamycin (C): Historically excellent anaerobic coverage; remains effective but increasing resistance rates reported in oral flora  — Azithromycin (D): Moderate coverage of some oral anaerobes but significantly inferior to metronidazole, amoxicillin-clavulanate, or clindamycin  Ciprofloxacin should not be used as monotherapy for odontogenic infections due to its poor anaerobic coverage. It may have a role in combination therapy when Gram-negative aerobic coverage is needed. | The correct answer is E) Ciprofloxacin.  Fluoroquinolone antibiotics — including ciprofloxacin, levofloxacin, and moxifloxacin — exhibit significant variability in their anaerobic spectrum. Ciprofloxacin, the most widely used first-generation fluoroquinolone, has characteristically poor activity against the anaerobic bacteria that dominate odontogenic infections.  Mechanism of Anaerobic Resistance to Ciprofloxacin:  Fluoroquinolones inhibit bacterial DNA gyrase (topoisomerase II) and topoisomerase IV, enzymes essential for DNA replication. Their relative inefficacy against anaerobes is attributed to:  1. Reduced outer membrane permeability of anaerobic organisms (limited drug penetration)  2. Absence of the oxygen-dependent processes that partly facilitate drug uptake  3. Constitutive efflux pump expression in some anaerobic species  Anaerobic Coverage Ranking (Oral Pathogens Context):  Excellent: Metronidazole, Amoxicillin-clavulanate, Clindamycin  Moderate: Azithromycin (some activity against Prevotella, Porphyromonas)  Poor/Inadequate: Ciprofloxacin — NOT recommended for odontogenic infections  The anaerobic pathogens most relevant to OMFS (Porphyromonas gingivalis, Prevotella intermedia, Fusobacterium nucleatum, Peptostreptococcus, Bacteroides) are largely resistant to ciprofloxacin.  Note: Option D (Azithromycin) also has limited anaerobic efficacy compared to first-line agents, making it a close second choice; however, ciprofloxacin consistently ranks lowest in published susceptibility data for oral anaerobes. | The correct answer is E) Ciprofloxacin.  Comparative Anaerobic Activity of Antibiotics Against Oral Microbial Flora  The oral cavity harbours the most complex microbial environment in the human body, with approximately 700 bacterial species identified. Odontogenic infections are characteristically polymicrobial, involving a combination of aerobic/facultative streptococci and obligate anaerobic Gram-negative rods and cocci. Among the listed antibiotics, ciprofloxacin has the lowest inherent efficacy against the anaerobic organisms that dominate this infection niche.  Anaerobic Activity Profiles:  Metronidazole (A): Gold standard for oral anaerobes  • Mechanism: Reduced to toxic nitroso radical inside strictly anaerobic cells; causes DNA strand breakage  • Spectrum: Excellent against virtually all clinically relevant obligate anaerobes (Bacteroides, Prevotella, Fusobacterium, Peptostreptococcus)  Amoxicillin-Clavulanate (B): Excellent broad-spectrum including anaerobes  • Clavulanate inhibits β-lactamase production by many anaerobic species that would otherwise hydrolyse amoxicillin  • First-line choice for most odontogenic infections  Clindamycin (C): Historically excellent anaerobic coverage  • Inhibits 50S ribosomal subunit; excellent tissue penetration  • Note: Increasing resistance rates in oral anaerobes (15–25% reported) diminish first-line status in some guidelines  Azithromycin (D): Moderate; variable against key oral anaerobes  • Some activity against Prevotella spp. and Porphyromonas spp., but inferior to amoxicillin-clavulanate, metronidazole, or clindamycin  Ciprofloxacin (E): Poorest anaerobic activity — CORRECT ANSWER  • Mechanism: DNA gyrase/topoisomerase IV inhibitor; minimal penetration into obligate anaerobic cells  • Spectrum: Excellent for aerobic Gram-negative rods (Enterobacteriaceae); severely limited anaerobic activity  • Not recommended as monotherapy or primary antibiotic in odontogenic infections  Clinical Implication: Ciprofloxacin is contraindicated as monotherapy for polymicrobial odontogenic infections. It may occasionally be used in combination with metronidazole (to add Gram-negative coverage) in special circumstances but should never replace agents with demonstrated anaerobic efficacy. |
| 23. **In a patient with a maxillofacial trauma and a confirmed cerebrospinal fluid (CSF) leak, what is the primary objective of empirical antibiotic therapy?**   **○ A) Prevention of endocarditis**  **○ B) Prevention of meningitis**  **○ C) Treatment of osteomyelitis**  **○ D) Suppression of soft tissue infection**  **○ E) Tetanus prophylaxis** | The correct answer is B) Prevention of meningitis.  In a patient with maxillofacial trauma and a confirmed cerebrospinal fluid (CSF) leak — whether rhinorrhoea or otorrhoea — the CSF leak represents a breach in the dura mater, creating a direct communication between the subarachnoid space (which contains CSF bathing the brain and spinal cord) and the nasal cavity, oral cavity, or external ear canal. These spaces harbour commensal bacteria, creating a significant risk for ascending bacterial infection.  The primary concern and therapeutic objective of empirical antibiotic therapy in this context is the prevention of bacterial meningitis — an infection of the meninges (the protective membranes surrounding the brain and spinal cord) that carries high morbidity and mortality if untreated. The organisms most commonly implicated in post-traumatic meningitis are Streptococcus pneumoniae, Haemophilus influenzae, and Staphylococcus aureus.  Note: The use of prophylactic antibiotics for CSF leaks remains somewhat controversial in the literature. Some systematic reviews suggest insufficient evidence for routine prophylaxis, while others and clinical guidelines recommend it for confirmed leaks. When prophylaxis is indicated, broad-spectrum antibiotics with good CNS penetration (e.g., ceftriaxone) are selected based on their activity against the most likely causative organisms.  The other options are incorrect: endocarditis prophylaxis is administered before dental procedures; osteomyelitis and soft tissue infections are not the primary concern of CSF-leak prophylaxis; and tetanus prophylaxis requires a toxoid/antitoxin, not antibiotics. | The correct answer is B) Prevention of meningitis.  A confirmed CSF leak (traumatic rhinorrhoea or otorrhoea) following maxillofacial trauma indicates a dural breach — typically associated with fractures of the anterior cranial base, cribriform plate, sphenoid sinus, petrous temporal bone, or posterior wall of the frontal sinus. This breach creates a bidirectional communication between the sterile subarachnoid space and the heavily colonised upper respiratory tract or external ear canal.  Primary Risk: Ascending Bacterial Meningitis  The dural breach allows retrograde bacterial ascent from colonised mucosal surfaces into the subarachnoid space, leading to bacterial meningitis. This complication is associated with:  • Mortality rates of 15–25% despite modern antibiotic therapy  • Significant rates of neurological sequelae in survivors (hearing loss, cognitive impairment, hydrocephalus)  Most Common Organisms in Post-Traumatic Meningitis:  • Streptococcus pneumoniae (most common)  • Haemophilus influenzae  • Staphylococcus species  • Gram-negative enteric organisms (less common)  Antibiotic Selection:  When prophylaxis is utilised (remains controversial per systematic reviews), agents with good CNS penetration are selected:  • Ceftriaxone (third-generation cephalosporin): Excellent CNS penetration, activity against S. pneumoniae and H. influenzae  • Amoxicillin-clavulanate: Moderate CNS penetration; alternative in certain settings  The remaining options (A, C, D, E) describe therapeutic objectives unrelated to the primary risk posed by a traumatic CSF fistula. | The correct answer is B) Prevention of meningitis.  Pathophysiology and Clinical Significance of Traumatic CSF Leak  Traumatic CSF fistulae most commonly arise from fractures involving the:  • Anterior cranial base and cribriform plate → CSF rhinorrhoea  • Posterior wall of the frontal sinus → CSF rhinorrhoea  • Petrous temporal bone / tegmen tympani → CSF otorrhoea  The dural laceration creates a persistent communication between two anatomically incompatible environments:  1. Subarachnoid space: Sterile, immunologically protected, containing CSF, meninges, and central nervous system  2. Paranasal sinuses / nasopharynx / external ear canal: Heavily colonised with commensal and potentially pathogenic bacteria  Primary Therapeutic Objective — Meningitis Prevention:  The critical risk is retrograde ascent of colonising bacteria (primarily Streptococcus pneumoniae, Haemophilus influenzae type b, and Staphylococcus aureus) through the dural defect into the subarachnoid space, causing:  • Bacterial meningitis: Mortality 15–30% in post-traumatic cases  • Ventriculitis and brain abscess (less common)  • Cranial nerve deficits, sensorineural hearing loss, hydrocephalus  Evidence on Antibiotic Prophylaxis:  The use of prophylactic antibiotics for traumatic CSF leaks remains controversial:  • Cochrane reviews (Ratilal et al.) found insufficient evidence to support routine antibiotic prophylaxis for all CSF leaks  • Current consensus: Prophylaxis may be considered for confirmed, persistent CSF leaks; the decision should be individualised  • When administered: Third-generation cephalosporins (ceftriaxone 2g IV) or amoxicillin 1g TID provide adequate empirical CNS-penetrating coverage  Systematic Elimination of Distractors:  — Prevention of endocarditis (A): Requires dental procedure-specific prophylaxis; unrelated to CSF fistula pathology  — Treatment of osteomyelitis (C): A delayed complication not addressed by empirical prophylaxis at time of injury  — Suppression of soft tissue infection (D): A local wound care concern, not the primary systemic risk  — Tetanus prophylaxis (E): Achieved with tetanus toxoid/immunoglobulin, not antibiotics; separate clinical pathway |
| 24. **In multimodal analgesia aimed at achieving an "opioid-sparing effect," which combination is most commonly utilized for managing acute postoperative pain?**   **○ A) Corticosteroids and NSAIDs**  **○ B) Local anesthetic injection only**  **○ C) NSAIDs and Paracetamol**  **○ D) High-dose Paracetamol and Benzodiazepines**  **○ E) Muscle relaxants and topical analgesics** | The correct answer is C) NSAIDs and Paracetamol.  Multimodal analgesia is a pain management strategy that combines analgesic agents with different mechanisms of action to achieve superior pain control with reduced doses of each individual agent — thereby minimising the adverse effects associated with higher doses, particularly opioid-related side effects such as respiratory depression, nausea, constipation, and dependence.  The most widely validated and clinically utilised combination for achieving an opioid-sparing effect in acute postoperative pain management is NSAIDs combined with paracetamol (acetaminophen):  — NSAIDs: Inhibit cyclooxygenase (COX-1 and COX-2), reducing peripheral prostaglandin synthesis, thereby attenuating peripheral sensitisation and the inflammatory component of pain  — Paracetamol: Acts primarily via central mechanisms (possible COX-3 inhibition, serotonergic modulation, and endocannabinoid system interaction), providing central analgesic augmentation without significant peripheral COX inhibition  When combined, these agents act at different anatomical and molecular targets, providing additive or synergistic analgesia that reduces the need for opioid supplementation by approximately 30–40%.  The other combinations listed are incorrect: corticosteroids and NSAIDs (A) have overlapping anti-inflammatory mechanisms and carry significant GI toxicity risk; local anaesthetic alone (B) is insufficient for multimodal systemic analgesia; high-dose paracetamol with benzodiazepines (D) provides sedation rather than true opioid-sparing analgesia; muscle relaxants with topical analgesics (E) are not a standard multimodal analgesic combination. | The correct answer is C) NSAIDs and Paracetamol.  Multimodal analgesia — the concurrent use of multiple analgesic agents targeting different components of the pain pathway — has become the standard of care for acute postoperative pain management in oral and maxillofacial surgery. The primary objective of the opioid-sparing strategy is to reduce opioid consumption (and its associated adverse effects) while maintaining adequate pain control.  The NSAID + Paracetamol Combination: Evidence and Mechanism  This combination works through complementary and partially synergistic mechanisms:  NSAIDs (e.g., ibuprofen, diclofenac, celecoxib):  • Peripheral COX inhibition → reduced prostaglandin synthesis at surgical site  • Attenuate peripheral sensitisation of nociceptors  • Anti-inflammatory and antipyretic effects  Paracetamol (Acetaminophen):  • Primarily central mechanism: inhibition of central COX enzymes (COX-3), modulation of the endocannabinoid system, activation of descending serotonergic pain inhibitory pathways  • Analgesic and antipyretic; no significant peripheral anti-inflammatory activity  • Distinct target profile complements NSAIDs  Combined Effect:  Meta-analyses consistently demonstrate that NSAID + paracetamol combination reduces opioid consumption by 30–40% compared to opioid monotherapy, with significantly improved pain scores and reduced opioid adverse effects.  This combination is recommended in perioperative analgesic guidelines by AAOMS, NICE, and the WHO pain management ladder as the foundational non-opioid analgesic regimen in oral surgery.  The remaining options (A, B, D, E) do not represent validated opioid-sparing combinations in the oral surgery context. | The correct answer is C) NSAIDs and Paracetamol.  The Pharmacological Basis of Opioid-Sparing Multimodal Analgesia  The concept of multimodal analgesia is grounded in the principle that acute postoperative pain is a multimechanistic phenomenon involving peripheral nociceptor sensitisation, central sensitisation, and descending modulatory pathway dysfunction. Targeting multiple mechanisms simultaneously with different drug classes allows each agent to be used at a lower dose, reducing adverse effects while achieving superior overall analgesia compared to single-agent high-dose therapy.  The NSAID + Paracetamol Combination: Mechanistic Complementarity  1. NSAIDs — Peripheral and Spinal Mechanisms:  • Cyclooxygenase (COX-1/COX-2 or selective COX-2) inhibition  • Reduction of prostaglandin E2 (PGE2), thromboxane, and prostacyclin at injury sites  • Attenuation of peripheral nociceptor sensitisation (primary hyperalgesia)  • Inhibition of spinal COX-2 (central COX inhibition contributing to secondary hyperalgesia prevention)  2. Paracetamol — Predominantly Central Mechanisms:  • Central COX inhibition (COX-3 hypothesis; supraspinal sites)  • Activation of descending serotonergic (5-HT3-mediated) inhibitory pain pathways  • Possible modulation of the endocannabinoid system (TRPV1 and CB1 receptor interactions)  • Enhancement of central pain inhibitory tone  Clinical Outcome Data:  Systematic reviews and meta-analyses of randomised controlled trials in oral and maxillofacial surgery demonstrate:  • NSAID + paracetamol combination reduces postoperative opioid consumption by 30–50% compared to opioid monotherapy  • Combination significantly superior to either agent alone for acute post-surgical pain  • Reduction in opioid-related adverse events (nausea, vomiting, respiratory depression, constipation)  • Improved patient satisfaction scores  Guideline Alignment:  • AAOMS Clinical Practice Guidelines  • NICE Guideline on Perioperative Care in Adults  • Enhanced Recovery After Surgery (ERAS) Society recommendations  All recommend NSAID + paracetamol as the first-line non-opioid backbone of multimodal analgesia.  Systematic Option Elimination:  — Corticosteroids + NSAIDs (A): Overlapping anti-inflammatory mechanisms; combination associated with significantly increased GI toxicity risk; NOT a standard opioid-sparing analgesic combination  — Local anaesthetic only (B): Adequate for intraoperative analgesia; insufficient as the sole analgesic component for systemic postoperative pain management  — High-dose paracetamol + benzodiazepines (D): Benzodiazepines are anxiolytics/sedatives with no direct analgesic activity; do not contribute to opioid-sparing analgesic effect  — Muscle relaxants + topical analgesics (E): Not a validated systemic opioid-sparing analgesic strategy in oral surgery |
| 25. **According to the 2021 American Heart Association (AHA) guidelines, clindamycin remains the first-choice alternative for infective endocarditis prophylaxis in patients with a penicillin allergy.** | The statement is incorrect according to the most recent 2021 American Heart Association (AHA) recommendations.  Evidence-Based Guideline Position  The AHA 2021 scientific update explicitly states that clindamycin is no longer recommended for infective endocarditis (IE) prophylaxis in patients undergoing dental procedures. It was removed from the prophylactic regimen because of its unfavorable safety profile, particularly:  Increased risk of Clostridioides difficile infection  Higher incidence of severe adverse drug reactions  No demonstrated superiority over safer alternatives  Recommended Alternatives for Patients with Penicillin Allergy  For patients allergic to penicillin or ampicillin who require IE prophylaxis before indicated dental procedures, the AHA recommends:  If oral medication can be taken:  Cephalexin (only if no history of immediate hypersensitivity/anaphylaxis to penicillin)  Azithromycin  Clarithromycin  Doxycycline  If unable to take oral medication:  Cefazolin  Ceftriaxone  Clinically Relevant Surgical Interpretation  In oral and maxillofacial surgery practice, this means that historical use of clindamycin as the routine substitute for amoxicillin in penicillin-allergic cardiac-risk patients is now outdated. Surgeons should revise legacy protocols and preoperative order sets accordingly.  Final Answer  False. According to the 2021 AHA guidelines, clindamycin does not remain the first-choice alternative; rather, it is no longer recommended for infective endocarditis prophylaxis. Safer alternatives such as azithromycin, clarithromycin, doxycycline, or selected cephalosporins are preferred. | Actually, the statement that clindamycin remains the first-choice alternative according to the 2021 American Heart Association (AHA) update is incorrect.As an Oral and Maxillofacial Surgeon, it is critical to note that the 2021 AHA scientific statement (specifically the focused update on the prevention of viridans group streptococcal infective endocarditis) made a landmark change to antibiotic prophylaxis protocols.1. The 2021 AHA Guideline ShiftThe most significant update in 2021 was the removal of clindamycin from the recommended regimens for dental prophylaxis.The rationale behind this removal includes:Adverse Effects: Clindamycin is associated with more frequent and severe adverse reactions compared to other antibiotics, most notably a high risk of Clostridioides difficile (C. diff) infection.Phasing Out: The AHA determined that the risks of using clindamycin for a single-dose prophylaxis outweigh the potential benefits, especially when safer alternatives exist.2. Revised Prophylaxis Protocols (Penicillin Allergy)For patients with a history of penicillin or amoxicillin allergy (non-anaphylactic or anaphylactic), the current first-choice alternatives for oral administration are:AntibioticAdult Dosage (Single Dose)Pediatric DosageCephalexin*2 g50 mg/kgAzithromycin or Clarithromycin500 mg15 mg/kgDoxycycline100 mg<45 kg: 2.2 mg/kg; >45 kg: 100 mg*Note: Cephalosporins should be avoided if the patient has a history of anaphylaxis, angioedema, or urticaria with penicillins.3. Clinical Implications for Oral SurgeryIn your clinical practice, particularly regarding dentoalveolar surgery or procedures involving manipulation of the gingival tissue or periapical region, you should prioritize Azithromycin or Doxycycline for penicillin-allergic patients who cannot take cephalosporins.Summary for Evidence-Based PracticeClindamycin: No longer recommended by the AHA/ADA for IE prophylaxis.Cephalexin: Preferred for non-anaphylactic penicillin allergies.Azithromycin/Doxycycline: Preferred for patients with a history of severe immediate-type hypersensitivity to penicillins.This change ensures a higher safety profile for our patients by mitigating the risk of antibiotic-associated colitis while still providing adequate coverage against viridans group streptococci. | FALSE.  This statement is definitively FALSE per the 2021 American Heart Association Scientific Statement on the Prevention of Viridans Group Streptococcal Infective Endocarditis (Wilson WR et al., Circulation. 2021;143:e963–e978).  Specific Change from Previous Guidelines:  The 2007 AHA guidelines designated clindamycin 600mg PO as the primary alternative for IE prophylaxis in patients with penicillin or ampicillin allergy who could not receive beta-lactam agents. This recommendation was universally adopted in dental and OMFS practice over the subsequent 14 years.  The 2021 AHA update removed clindamycin from the recommended alternatives list entirely. The updated statement explicitly states that clindamycin "is no longer recommended" as an alternative for IE prophylaxis.  Evidence Supporting Removal:  The decision was driven by pharmacoepidemiological data demonstrating that clindamycin, even as a single prophylactic dose, significantly disrupts the gut microbiome and is associated with:  • Elevated rates of Clostridioides difficile colonisation and toxin production  • Risk of C. difficile-associated diarrhoea (CDAD) and pseudomembranous colitis  • Potential for fatal C. difficile colitis, particularly in elderly patients with cardiac comorbidities who are the primary recipients of IE prophylaxis  The risk-benefit analysis therefore shifted unfavourably against clindamycin.  Current Recommended Alternatives (2021 AHA) for Non-Beta-Lactam-Tolerant Patients:  1. Azithromycin 500mg PO — 30–60 minutes pre-procedure  2. Clarithromycin 500mg PO — 30–60 minutes pre-procedure  3. Doxycycline 100mg PO — 30–60 minutes pre-procedure (new; for macrolide-intolerant patients)  Key Clinical Implication for OMFS:  Any practitioner still prescribing clindamycin for IE prophylaxis is not following current AHA 2021 evidence-based guidelines, exposing patients to preventable risk of C. difficile infection. Patient records and prescribing protocols should be updated accordingly. |
| 26. **The routine prescription of systemic antibiotics following the surgical extraction of impacted third molars in healthy individuals is strictly indicated to prevent infection.** | The statement is incorrect according to contemporary evidence-based oral and maxillofacial surgery principles.  Evidence-Based Clinical Position  The routine prescription of systemic antibiotics after surgical removal of impacted third molars in healthy individuals is not strictly indicated. Current literature, including systematic reviews and meta-analyses, demonstrates that while antibiotics may modestly reduce postoperative infection rates, this benefit does not justify universal postoperative prescribing in otherwise healthy, immunocompetent patients undergoing uncomplicated surgery.  Multiple reviews conclude that routine use is not supported, particularly when weighed against:  Antimicrobial resistance  Allergic reactions  Gastrointestinal adverse effects  Clostridioides difficile infection risk  Unnecessary medicalization of self-limiting postoperative inflammation  What Current Evidence Suggests  A Cochrane review found antibiotics may reduce postoperative infection after impacted third molar surgery, but the absolute benefit is limited: approximately 19 patients need treatment to prevent one infection. This indicates a relatively low baseline infection risk in healthy patients and argues against indiscriminate prescribing.  When Antibiotics May Be Justified  Selective postoperative or perioperative antibiotics may be considered in higher-risk scenarios such as:  Immunocompromised patients  Poorly controlled diabetes mellitus  Existing acute odontogenic infection/pericoronitis with spread  Prolonged, traumatic, or contaminated surgery  Extensive bone removal with difficult flap management  History of recurrent postoperative infection  ASA-compromised patients with reduced host defense  Preferred Modern Approach in Healthy Patients  For routine impacted third molar surgery in healthy individuals, best practice usually emphasizes:  Strict aseptic surgical technique  Copious irrigation  Minimal tissue trauma  Adequate debridement  Chlorhexidine adjuncts when appropriate  NSAID-based analgesia  Clear postoperative instructions and follow-up  Practical OMFS Conclusion  The statement is false. Routine postoperative systemic antibiotics after impacted third molar extraction in healthy patients are not strictly indicated and should not be prescribed automatically. Use should be case-selected, based on surgical difficulty, contamination level, and patient-specific risk factors. | The statement that routine systemic antibiotic prescription is "strictly indicated" for the surgical extraction of impacted third molars in healthy individuals is clinically and scientifically incorrect according to contemporary evidence-based oral and maxillofacial surgery (OMFS) protocols.  As a specialist in this field, it is essential to distinguish between therapeutic use and prophylactic use, while also adhering to the principles of antibiotic stewardship to combat global antimicrobial resistance.  1. Current Scientific Consensus  Recent meta-analyses and systematic reviews (including those by the Cochrane Collaboration) indicate that while antibiotics can reduce the risk of postoperative infection and alveolar osteitis (dry socket), the Number Needed to Treat (NNT) is high. This means many healthy patients must be exposed to the risks of antibiotics to prevent a single case of infection.  In healthy (ASA I or II) patients, the primary prevention of postoperative complications relies on surgical technique, aseptic protocols, and local measures rather than systemic intervention.  2. Clinical Indications for Antibiotic Use  Following international guidelines (such as those from the AAOMS and SDCEP), systemic antibiotics should be reserved for specific scenarios rather than used "routinely":  Prophylactic Use (Single Dose): A single pre-operative dose of Amoxicillin (2g) may be considered in complex surgical cases involving significant bone removal or prolonged surgical time. This is intended to achieve high plasma concentrations during the procedure.  Therapeutic Use: Indicated only when signs of spreading infection (cellulitis, trismus, lymphadenopathy, or systemic malaise) are present, or in patients who are immunocompromised (e.g., uncontrolled diabetes, chemotherapy, or chronic corticosteroid use).  3. Risks of Over-Prescription  Routine prescription in healthy individuals is discouraged due to:  Antibiotic Resistance: Contributing to the development of multi-drug resistant organisms.  Adverse Effects: Risks of gastrointestinal distress, allergic reactions (anaphylaxis), and Clostridioides difficile infections.  Dysbiosis: Disruption of the patient's microbiome without a clear clinical necessity.  4. Evidence-Based Recommendations for Postoperative Care  For the management of impacted third molars in healthy patients, the focus should be on:  Strict Surgical Asepsis: Proper flap design and cooling during ostectomy.  Local Antimicrobials: Use of Chlorhexidine (0.12% - 0.2%) rinses or gels peri-operatively, which has been shown to be effective in reducing the incidence of alveolar osteitis with fewer systemic risks.  Analgesic Protocols: Utilizing a combination of NSAIDs and Acetaminophen (Paracetamol) for inflammation and pain management.  Conclusion  In conclusion, the routine use of systemic antibiotics for third molar extractions in healthy individuals is not supported by current international guidelines. Professional judgment should favor a case-by-case assessment, prioritizing prophylaxis only for high-risk surgical sites or medically compromised patients. | FALSE.  This statement is categorically false and represents a prescribing pattern that is explicitly discouraged by current antimicrobial stewardship guidelines, Cochrane-level evidence, and the position statements of major oral and maxillofacial surgery organisations.  Evidence Summary:  A 2021 Cochrane systematic review (Lodi et al.) on antibiotics to prevent complications following tooth extractions evaluated 23 RCTs including over 3,000 participants. Key findings:  • Preoperative antibiotics (single dose) moderately reduced the risk of infection (RR 0.29) and alveolar osteitis (RR 0.38) compared to no antibiotics  • However, the absolute risk reduction was modest (approximately 4–5 infections prevented per 100 procedures)  • Postoperative antibiotic courses did not provide additional benefit over single preoperative doses  • Antibiotic-related adverse events must be weighed against this limited benefit  Current Guideline Position (AAOMS, WHO, ADA):  • Routine postoperative antibiotic prescription for impacted third molar surgery in healthy individuals is NOT indicated  • The overuse of antibiotics in oral surgery is a recognised driver of antimicrobial resistance  • Antimicrobial stewardship principles mandate restriction of antibiotic use to cases where clear clinical benefit exists  When Antibiotics Are Clinically Justified in Third Molar Surgery:  1. Immunocompromised patients (HIV/AIDS, post-solid organ transplant, cytotoxic chemotherapy)  2. Acute pericoronitis with systemic signs (fever >38°C, lymphadenopathy, trismus)  3. Spreading deep space infection or cellulitis  4. Osteomyelitis  5. Patients with uncontrolled systemic disease (uncontrolled diabetes, haematological disorders)  6. Severe local infection requiring surgical drainage  For a routine healthy patient undergoing elective third molar extraction: no postoperative antibiotic is indicated. Local measures (chlorhexidine irrigation, adequate curettage, haemostasis) are the primary preventive strategy for alveolar osteitis. |
| 27. **In patients taking bisphosphonates or antiangiogenic drugs, tooth extraction or surgical bone trauma is recognized as a major triggering factor for the development of MRONJ.** | The statement is substantially correct and is consistent with current international clinical understanding of medication-related osteonecrosis of the jaw (MRONJ).  In patients receiving bisphosphonates (particularly intravenous bisphosphonates such as zoledronic acid, pamidronate) or antiangiogenic drugs (such as bevacizumab, sunitinib, denosumab), tooth extraction and other surgical procedures involving bone trauma are recognised as major triggering factors for the development of Medication-Related Osteonecrosis of the Jaw (MRONJ).  MRONJ is defined as exposed or necrotic bone in the maxillofacial region that persists for more than 8 weeks in patients with a history of antiresorptive or antiangiogenic therapy, in the absence of prior radiation therapy or metastatic disease.  Why Surgical Dental Procedures Trigger MRONJ: Bisphosphonates are incorporated into bone and inhibit osteoclast function, impairing bone remodelling and the normal healing response to surgical trauma. When the protective mucosal barrier is disrupted by extraction or other surgical trauma, the underlying avascular, non-remodelling bone cannot mount an adequate healing response, leading to exposure and progressive necrosis.  Risk is particularly elevated with: — Intravenous bisphosphonates (used in oncology) vs. oral bisphosphonates (used in osteoporosis) — Duration of therapy — Concurrent corticosteroid use — Pre-existing dental disease  Clinical implication: All patients on antiresorptive or antiangiogenic therapy should receive comprehensive dental evaluation and necessary extractions before initiating medical therapy. When extractions cannot be avoided in medicated patients, protocols to minimise MRONJ risk (aseptic technique, primary closure, antibiotic coverage, cessation of therapy if feasible) should be followed. | This statement is clinically accurate and represents a fundamental pillar in the prevention and management of Medication-Related Osteonecrosis of the Jaw (MRONJ).  Tooth extraction and other forms of surgical bone trauma are consistently identified in the published literature and clinical guidelines as the most common triggering events for Medication-Related Osteonecrosis of the Jaw (MRONJ) in patients receiving antiresorptive or antiangiogenic pharmacotherapy.  Epidemiological Evidence: Multiple case series and prospective studies report that tooth extraction is associated with approximately 50–70% of MRONJ cases in patients receiving intravenous bisphosphonates. The risk is substantially elevated: • IV bisphosphonates (zoledronic acid, pamidronate): 0.8–12% risk of MRONJ after dental extraction • Oral bisphosphonates (alendronate, risedronate): 0.01–0.04% risk after dental extraction • Denosumab (RANK-L inhibitor): Similar risk profile to IV bisphosphonates with shorter reversibility window  Pathophysiological Basis: The mechanism involves several converging factors: 1. Bisphosphonate-induced osteoclast suppression → impaired bone remodelling 2. Anti-angiogenic effects of some agents → reduced vascularity and healing capacity 3. Surgical wound creates entry point for oral bacteria into non-remodelling bone 4. Chronic exposure of avascular, bisphosphonate-laden bone to the oral environment  Preventive Approach: The AAOMS and AOOS guidelines advocate for dental screening and prophylactic treatment before initiating antiresorptive therapy. A drug holiday prior to elective dental surgery is considered in appropriate cases. | TRUE.  This statement is TRUE and represents a cornerstone of MRONJ pathophysiology, prevention, and management.  MRONJ Definition (AAOMS Position Paper 2022): Medication-Related Osteonecrosis of the Jaw is defined as exposed bone or bone that can be probed through an intraoral or extraoral fistula in the maxillofacial region that has persisted for more than 8 weeks, in a patient with current or prior exposure to antiresorptive or antiangiogenic agents, in the absence of jaw irradiation or metastatic disease.  Triggering Role of Surgical Dental Procedures: Tooth extraction is the single most common identifiable trigger for MRONJ, accounting for approximately 52–61% of cases across published case series. Other triggering procedures include: • Dental implant placement • Periodontal surgery • Endodontic surgery/apicoectomy • Mandibular tori removal • Any procedure that violates the oral mucosa over bone  Incidence Data by Drug Category: • Intravenous zoledronic acid (oncology dose): MRONJ risk after extraction 0.8–6.7% • Oral alendronate (osteoporosis dose): Risk after extraction 0.04–0.1% • Denosumab 60mg (osteoporosis): Risk similar to oral bisphosphonates • Denosumab 120mg (oncology): Risk similar to IV bisphosphonates • Bevacizumab / Sunitinib: 0.2–0.3% risk  Pathophysiological Framework: 1. Antiresorptive effect: Bisphosphonates are irreversibly incorporated into bone and suppress osteoclastic bone remodelling (>10-year skeletal retention half-life for zoledronic acid). This prevents the normal bone repair response to surgical trauma. 2. Antiangiogenic effect: Reduced vascular supply (seen with bisphosphonates and particularly with VEGF inhibitors) impairs wound healing and creates avascular zones vulnerable to osteonecrosis. 3. Barrier disruption: Extraction wounds disrupt the oral mucosal protective barrier, exposing devascularised, non-remodelling bone to the polymicrobial oral environment. 4. Infection: Secondary colonisation by oral bacteria (particularly Actinomyces species) perpetuates inflammatory bone destruction.  Clinical Management Implications: Pre-therapy dental clearance: All necessary extractions and surgical procedures should ideally be completed 4–6 weeks before initiating antiresorptive/antiangiogenic therapy, allowing adequate mucosal healing. During therapy: Prioritise non-surgical dental management; extractions only when medically necessary with optimised technique (primary closure, antibiotic coverage, minimal trauma). Drug holiday consideration: Temporary cessation of oral bisphosphonates or denosumab prior to elective oral surgery may reduce risk in long-term users, though evidence is moderate. |
| 28. **Paracetamol (Acetaminophen) exhibits potent anti-inflammatory properties and is as effective as NSAIDs in controlling post-surgical bone edema.** | The statement is incorrect according to current pharmacologic evidence and contemporary oral surgery practice.  Evidence-Based Clinical Position  Paracetamol (acetaminophen) is primarily an analgesic and antipyretic agent. It does not exhibit potent peripheral anti-inflammatory activity comparable to nonsteroidal anti-inflammatory drugs (NSAIDs). Its mechanism of action is largely central nervous system mediated, with only weak peripheral cyclooxygenase inhibition. Therefore, it is not equivalent to NSAIDs for suppression of postoperative inflammatory edema.  Relevance in Oral and Maxillofacial Surgery  Following procedures such as:  impacted third molar surgery  osteotomy  flap elevation with bone removal  implant surgery with grafting  facial trauma surgery  postoperative swelling is driven largely by local inflammatory mediator release (especially prostaglandins, bradykinin, cytokines). NSAIDs directly inhibit prostaglandin synthesis and therefore have a stronger biologic rationale for reducing:  edema  trismus  inflammatory pain  Recent oral surgery evidence continues to support NSAIDs as useful agents for postoperative swelling control, although study quality varies.  What Paracetamol Does Well  Paracetamol remains highly valuable for:  mild to moderate postoperative pain  opioid-sparing multimodal analgesia  patients who cannot tolerate NSAIDs  combination regimens with ibuprofen or other NSAIDs  It is effective for pain after oral surgery, but that should not be confused with strong anti-inflammatory efficacy.  Important Nuance  Some individual studies have reported swelling outcomes similar to or better than specific NSAIDs in selected settings, but these findings do not overturn the broader pharmacologic consensus that paracetamol is not a potent anti-inflammatory drug and should not be considered generally equivalent to NSAIDs for postoperative bone/soft tissue edema control.  Practical OMFS Conclusion  For routine dentoalveolar surgery:  NSAIDs = preferred first-line when inflammation/swelling predominates and no contraindication exists  Paracetamol = excellent analgesic adjunct or alternative when NSAIDs are contraindicated  NSAID + paracetamol combination = often superior analgesic strategy compared with either alone  Final Answer  False. Paracetamol does not possess potent anti-inflammatory properties and is not generally as effective as NSAIDs in controlling post-surgical bone edema. Its principal benefit is analgesia, whereas NSAIDs remain more effective for inflammation-mediated postoperative swelling. | The statement that Paracetamol (Acetaminophen) exhibits "potent anti-inflammatory properties" and is as effective as Non-Steroidal Anti-Inflammatory Drugs (NSAIDs) in controlling post-surgical bone edema is pharmacologically and clinically incorrect.As an Oral and Maxillofacial Surgeon, it is critical to distinguish between the mechanisms of action of these two classes of analgesics, particularly when managing the inflammatory cascade following dentoalveolar or reconstructive bone surgery.1. Mechanism of Action: The Critical DistinctionThe primary reason this statement is incorrect lies in the site of action and the biochemical pathways involved:NSAIDs (e.g., Ibuprofen, Diclofenac, Naproxen, Celecoxib): These agents act by inhibiting the Cyclooxygenase (COX-1 and COX-2) enzymes peripherally. This inhibition directly reduces the synthesis of pro-inflammatory prostaglandins ($PGE_2$) at the site of surgical trauma. By reducing prostaglandin levels in the periodontal tissues and alveolar bone, NSAIDs effectively decrease vasodilation, vascular permeability, and subsequent post-surgical edema.Paracetamol (Acetaminophen): While its exact mechanism is still debated, it is primarily considered a central analgesic. It is believed to inhibit prostaglandin synthesis in the Central Nervous System (CNS) and may interact with the endocannabinoid system or the descending inhibitory serotonergic pathways. However, it has negligible peripheral anti-inflammatory activity because it is inactivated by peroxides produced at the site of active inflammation.2. Management of Post-Surgical Bone EdemaPost-surgical edema following procedures such as the extraction of impacted third molars or dental implant placement is driven by the release of inflammatory mediators (bradykinin, histamines, and prostaglandins) in the surgical field.Efficacy Comparison: Clinical trials and systematic reviews consistently demonstrate that NSAIDs are superior to Paracetamol for the management of post-operative swelling (edema) and trismus. Paracetamol, while effective for mild-to-moderate pain, does not possess the biochemical capacity to modulate the peripheral vascular changes that lead to tissue swelling.Bone Edema: NSAIDs are particularly effective in bone surgery as they inhibit the prostaglandins involved in bone resorptive signaling and inflammatory vascular expansion within the marrow spaces.3. Clinical Application: Multimodal AnalgesiaIn modern Oral and Maxillofacial Surgery, the "Gold Standard" is not to choose one over the other, but rather to utilize Multimodal Analgesia.International guidelines (such as those from the ADA and AAOMS) recommend the synergistic combination of Paracetamol and NSAIDs:Synergy: Since they act through different pathways (central vs. peripheral), taking them together provides superior pain relief compared to either drug alone at higher doses.Dosing Strategy: A common evidence-based protocol is the simultaneous or staggered administration of 400–600 mg Ibuprofen with 1000 mg Paracetamol.Steroidal Adjuncts: In cases where severe edema is anticipated (e.g., deeply impacted mandibular molars or orthognathic surgery), the use of corticosteroids (e.g., Dexamethasone) is the primary method for controlling edema, as they act higher up in the inflammatory cascade by inhibiting phospholipase $A_2$.ConclusionParacetamol is an excellent analgesic and antipyretic with a high safety profile, but it is not an anti-inflammatory agent. For the specific clinical goal of controlling post-surgical bone edema and peripheral inflammation, NSAIDs remain the pharmacological first-choice. | FALSE.  This statement is pharmacologically false and represents a clinically important distinction in analgesic prescribing.  Paracetamol: Mechanism and Anti-inflammatory Profile  Mechanism of Action: Paracetamol's analgesic and antipyretic mechanisms are primarily central: 1. COX inhibition: Paracetamol does inhibit COX enzymes, but primarily in the central nervous system (hypothalamic temperature regulation, CNS pain modulation). Peripheral COX inhibition is clinically insignificant at therapeutic doses, particularly in the presence of oxidative species (lipid peroxides) found at sites of inflammation — which paradoxically reduce paracetamol's COX inhibitory potency. 2. Endocannabinoid modulation: Paracetamol is metabolised to AM404 (arachidonoyl aminophenol), a cannabinoid receptor agonist that contributes to central analgesic effects via CB1 and TRPV1 receptors. 3. Serotonergic pathway activation: Enhancement of descending serotonergic inhibitory pain control via 5-HT₃ receptor interactions.  Net Result: NO clinically meaningful peripheral anti-inflammatory activity. Paracetamol does not suppress PGE2 synthesis at sites of tissue injury, does not reduce post-surgical oedema formation, and does not inhibit the inflammatory cascade driving post-surgical bone swelling.  NSAIDs: Peripheral Anti-Inflammatory Mechanism  Non-steroidal anti-inflammatory drugs potently inhibit peripheral COX-1 and/or COX-2 at the site of inflammation, reducing: • Prostaglandin E2 (PGE2): Primary mediator of vasodilation, vascular permeability increase, nociceptor sensitisation • Prostacyclin (PGI2): Vasodilatory prostaglandin amplifying oedema • Thromboxane A2: Promotes platelet aggregation and vasoconstriction • Leukotrienes (via downstream effects): Neutrophil chemotaxis reduction  This peripheral inhibition produces genuine anti-inflammatory, anti-oedema effects that paracetamol cannot replicate.  Clinical Implication in OMFS: In the context of post-surgical bone oedema following orthognathic surgery, third molar extraction, or implant surgery: • NSAIDs provide direct anti-inflammatory and anti-oedema effects • Corticosteroids (dexamethasone) provide the most potent anti-oedema effect via membrane-level phospholipase A2 inhibition • Paracetamol provides central analgesia WITHOUT anti-inflammatory benefit • Combination of NSAIDs + paracetamol targets both peripheral inflammation and central pain amplification — but paracetamol's contribution is analgesic, not anti-inflammatory |
| 29. **Systemic antibiotics are not indicated in cases of acute pericoronitis if there are no signs of systemic involvement, such as fever, lymphadenopathy, or trismus.** | The statement is generally correct, with important clinical nuance.  Evidence-Based Clinical Position  In acute localized pericoronitis without evidence of systemic spread or significant fascial space involvement, systemic antibiotics are usually not indicated as first-line treatment. Contemporary dental and oral surgery antimicrobial stewardship principles recommend that local measures should be prioritized when infection remains confined to the pericoronal soft tissues and the patient is systemically well.  Routine antibiotic prescribing for uncomplicated pericoronitis contributes to:  Antimicrobial resistance  Adverse drug reactions  Gastrointestinal complications  Unnecessary masking of progressive disease  First-Line Management in Localized Pericoronitis  When there is no fever, malaise, cellulitis, progressive swelling, dysphagia, or significant trismus, management should focus on elimination of the local inflammatory source:  Irrigation beneath the operculum (saline / chlorhexidine where appropriate)  Debridement of food debris and plaque  Occlusal trauma reduction if opposing tooth trauma exists  Analgesics / NSAIDs if appropriate  Improved oral hygiene instruction  Short-interval reassessment  Definitive treatment after acute phase: operculectomy or extraction of the involved third molar when indicated  When Systemic Antibiotics Become Indicated  Antibiotics should be considered when systemic involvement or spreading infection is present, including:  Fever  Malaise  Regional lymphadenopathy with active infection  Progressive swelling / cellulitis  Significant trismus suggesting deeper spread  Dysphagia / odynophagia  Immunocompromised status  Inability to achieve drainage/local control  Important Nuance About Trismus  The statement lists trismus among systemic signs. Trismus itself is not a systemic sign; it is a local clinical warning sign that may indicate extension of inflammation into masticatory spaces. Nonetheless, its presence often supports escalation of treatment, including possible antibiotic therapy.  Practical OMFS Conclusion  Localized pericoronitis should not be treated reflexively with antibiotics. Failure to provide local debridement while prescribing antibiotics alone is poor practice and associated with recurrence.  Final Answer  True in principle. In acute pericoronitis without systemic involvement or evidence of spreading local infection, systemic antibiotics are not routinely indicated. Local operative management and reassessment are preferred. However, if fever, cellulitis, progressive swelling, significant trismus, lymphadenopathy with active spread, or host-risk factors are present, antibiotics may be justified. | The statement that systemic antibiotics are not indicated in cases of acute pericoronitis without systemic involvement is clinically accurate and aligns with the current Stewardship of Antibiotics in Dentistry guidelines published by international bodies such as the FDI World Dental Federation, SDCEP, and AAOMS.  As a specialist, it is imperative to manage pericoronitis—a common inflammatory condition of the soft tissues (operculum) surrounding a partially erupted tooth—based on the severity of the clinical presentation rather than a reflexive prescription of antimicrobial agents.  1. Clinical Classification and Indications  Pericoronitis is categorized into localized and systemic forms. The therapeutic approach is dictated by this distinction:  Localized Pericoronitis: The inflammation is confined to the operculum. Symptoms include localized pain, edema, and discomfort during mastication. In these cases, systemic antibiotics provide no significant benefit over local mechanical debridement.  Systemic/Spreading Pericoronitis: This is characterized by the "red flags" mentioned in your question: fever (pyrexia), submandibular lymphadenopathy, trismus (indicating involvement of the masticatory muscles, specifically the medial pterygoid), or facial cellulitis. Systemic antibiotic therapy is mandatory here to prevent the progression into deep fascial space infections (e.g., Ludwig’s Angina).  2. Evidence-Based Local Management  For localized cases, the "Gold Standard" of treatment focuses on removing the bacterial load and debris (biofilm) from the sub-opercular space:  Debridement and Irrigation: Copious irrigation of the pericoronal space using 0.12% Chlorhexidine digluconate or sterile saline to remove food debris and necrotic tissue.  Mechanical Drainage: If a localized fluctuant abscess is present under the operculum, cautious incision and drainage may be performed.  Occlusal Adjustment: Evaluating the opposing maxillary third molar. If it is traumatizing the inflamed operculum, selective grinding or extraction of the opposing tooth may be necessary to alleviate mechanical irritation.  Operculectomy: Surgical removal of the soft tissue flap once the acute phase has subsided, although extraction of the impacted tooth remains the definitive long-term solution.  3. Pharmacological Rationale  When systemic involvement warrants antibiotics, the choice of agent must cover the typical anaerobic and facultative anaerobic flora of the oral cavity (e.g., Prevotella, Fusobacterium, and Streptococcus species):  First-line Therapy: Amoxicillin (500 mg every 8 hours) or Amoxicillin with Clavulanic Acid (875/125 mg every 12 hours).  For Penicillin-Allergic Patients: Metronidazole (400–500 mg every 8 hours) is highly effective against the anaerobic components of pericoronitis.  Adjunctive Therapy: Prescription of NSAIDs (e.g., Ibuprofen) is indicated for pain and to reduce the localized inflammatory response.  4. Clinical Significance in Oral Surgery  Over-reliance on antibiotics in localized pericoronitis contributes to antimicrobial resistance and may mask the need for definitive surgical intervention (extraction). Current guidelines emphasize that antibiotics are an adjunct, not a substitute, for surgical and mechanical local treatment.  Conclusion  In the absence of systemic manifestations (fever, malaise, lymphadenopathy, or limited mouth opening), the management of acute pericoronitis should remain strictly local and mechanical. Systemic antibiotics should be reserved for cases where the infection shows potential for regional or systemic spread, ensuring both patient safety and responsible prescribing habits. | TRUE.  This statement is TRUE and reflects the current evidence-based consensus from antimicrobial stewardship guidelines, systematic reviews, and OMFS clinical practice guidelines.  Pathophysiology and Clinical Stratification of Pericoronitis:  Pericoronitis arises from bacterial colonisation of the pericoronal space under the operculum of a partially erupted mandibular (or occasionally maxillary) third molar. The clinical presentation ranges from localised mild inflammation to severe spreading infection requiring urgent surgical management.  Definitive Management Based on Severity:  Localised Pericoronitis (No Systemic Features):  Assessment criteria confirming absence of systemic involvement:  • Temperature: Afebrile (<37.5°C)  • Lymphadenopathy: Absent  • Trismus: None or minimal (functional jaw opening preserved)  • Swelling: Confined to pericoronal tissues; no facial space involvement  • Airway: Uncompromised  • Dysphagia: Absent  Management:  1. Local irrigation: Thorough debridement and irrigation of pericoronal space with chlorhexidine 0.12–0.2% or saline under the operculum  2. Oral hygiene reinforcement  3. Analgesics: NSAIDs (ibuprofen 400mg TID) ± paracetamol for pain control  4. Definitive treatment: Extraction of the causative third molar (usually at a subsequent appointment)  5. ANTIBIOTICS: NOT indicated — local measures are sufficient  Spreading Pericoronitis (With Systemic Features — Antibiotics Indicated):  Indication for antibiotics: Presence of ANY of:  • Fever ≥38°C  • Cervical/submandibular lymphadenopathy  • Trismus (reduced inter-incisal opening <25mm)  • Spread to pterygomandibular, buccal, or deep cervical spaces  • General malaise, dysphagia, airway compromise  Antibiotic of Choice:  • Amoxicillin-clavulanate 875/125mg TID PO × 5 days (first-line)  • Alternatively: Penicillin V + metronidazole 400mg TID (combined)  • Penicillin allergy: Clindamycin 300mg TID or azithromycin 500mg OD × 3 days  Antibiotic Stewardship Alignment:  The WHO, ADA, and NICE explicitly discourage antibiotic prescription for localised pericoronitis without systemic features, identifying this as a major source of inappropriate antibiotic use in dentistry contributing to global antimicrobial resistance. |
| 30. **Macrolide antibiotics (e.g., erythromycin, clarithromycin) can inhibit cytochrome P450 enzymes, thereby increasing the toxicity of co-administered drugs like anticoagulants.** | The statement is correct.  Evidence-Based Pharmacology  Certain macrolide antibiotics, particularly erythromycin and clarithromycin, are clinically relevant inhibitors of the cytochrome P450 (CYP450) enzyme system, especially the CYP3A4 isoenzyme. This inhibition can significantly alter the metabolism of co-administered drugs.  Mechanism of Interaction  Clarithromycin and erythromycin inhibit hepatic CYP3A4  This leads to reduced clearance and increased plasma concentration of susceptible drugs  The result is a dose-dependent risk of toxicity or exaggerated pharmacologic effects  Interaction with Anticoagulants  The statement specifically mentions anticoagulants, which is partly correct but needs nuance:  1. Vitamin K antagonists (e.g., warfarin)  Macrolides may increase INR and bleeding risk  Mechanisms:  CYP450 inhibition → reduced warfarin metabolism  Possible alteration of gut flora affecting vitamin K synthesis  This is a clinically significant and well-documented interaction  2. Direct oral anticoagulants (DOACs)  Interactions are more variable:  Some DOACs (e.g., apixaban, rivaroxaban) are partially CYP3A4 substrates  Macrolides may increase plasma levels, though clinical significance is generally lower than with warfarin  Risk is higher in patients with renal impairment or polypharmacy  Clinical Relevance in Oral and Maxillofacial Surgery  In OMFS practice, macrolide–drug interactions are particularly relevant when prescribing antibiotics to patients who may be taking:  Anticoagulants (warfarin, DOACs)  Antiarrhythmics  Statins (especially simvastatin, atorvastatin)  Immunosuppressants (cyclosporine, tacrolimus)  Failure to recognize CYP3A4 inhibition can lead to:  Hemorrhagic complications in surgical patients  Drug toxicity (e.g., myopathy with statins, arrhythmia risk with QT-prolonging drugs)  Important Nuance  Not all macrolides have the same interaction profile:  Clarithromycin / erythromycin → strong CYP3A4 inhibitors (clinically significant interactions)  Azithromycin → minimal CYP450 inhibition (preferred in patients on multiple medications)  Practical OMFS Implication  In patients requiring antibiotic prophylaxis or treatment:  Prefer azithromycin over clarithromycin/erythromycin when drug interactions are a concern  Review anticoagulant therapy carefully preoperatively  Monitor INR closely if warfarin cannot be avoided  Final Answer  True, but incomplete. Macrolides such as erythromycin and clarithromycin inhibit CYP3A4, which can increase serum levels and toxicity of co-administered drugs, including warfarin and some direct oral anticoagulants, leading to a higher risk of bleeding and other adverse effects. However, this interaction is not uniform across all macrolides, as azithromycin has minimal CYP450 inhibition and a significantly safer interaction profile. | The statement that macrolide antibiotics can inhibit the cytochrome P450 (CYP450) enzyme system and significantly increase the toxicity of co-administered drugs, such as certain anticoagulants, is pharmacologically accurate and represents a critical clinical consideration in Oral and Maxillofacial Surgery.  As a specialist, managing patients on polypharmacy requires a profound understanding of these drug-drug interactions (DDIs) to prevent life-threatening complications, particularly postoperative hemorrhage.  1. The Biochemical Mechanism: CYP3A4 Inhibition  The interaction primarily involves the CYP3A4 isoenzyme, which is responsible for the metabolism of approximately 50% of all prescribed medications.  Enzyme Inhibition: Macrolides—specifically erythromycin and clarithromycin—act as potent inhibitors of CYP3A4. They form a metabolic intermediate complex with the iron atom of the cytochrome P450 heme, effectively inactivating the enzyme.  Bioavailability and Clearance: When the CYP3A4 enzyme is inhibited, the hepatic metabolism of "substrate" drugs is slowed. This leads to elevated plasma concentrations, prolonged half-lives, and a heightened risk of toxicity.  Notable Exception: Azithromycin is a unique macrolide (an azalide) that does not significantly inhibit the CYP450 system. Consequently, it is often the preferred macrolide in patients taking medications metabolized by CYP3A4.  2. Interaction with Anticoagulants and Cardiovascular Agents  For an Oral and Maxillofacial Surgeon, the most dangerous interactions occur with anticoagulants and other drugs commonly managed in the surgical setting:  Warfarin (Vitamin K Antagonist): Macrolides inhibit the metabolism of warfarin (though warfarin is primarily metabolized by CYP2C9, there is secondary CYP3A4 involvement, and macrolides also disrupt Vitamin K-producing gut flora). This can lead to a dramatic increase in the International Normalized Ratio (INR), resulting in spontaneous bleeding or uncontrollable postoperative hemorrhage.  Direct Oral Anticoagulants (DOACs): Agents like Rivaroxaban and Apixaban are substrates of CYP3A4. Co-administration with clarithromycin can lead to increased serum levels of these drugs, significantly elevating the risk of major bleeding events.  Statins (e.g., Simvastatin, Atorvastatin): Inhibition of CYP3A4 can lead to toxic levels of statins, increasing the risk of rhabdomyolysis and acute renal failure.  3. Clinical Application in Maxillofacial Surgery  When prescribing antibiotics for odontogenic infections or postoperative prophylaxis, the following evidence-based precautions must be observed:  Pharmacological Screening: Always conduct a thorough review of the patient's current medication list, specifically looking for anticoagulants, statins, or anti-epileptic drugs (like carbamazepine).  Selection of Alternative Antimicrobials:  If a macrolide is necessary in a patient taking CYP3A4 substrates, Azithromycin is the evidence-based alternative due to its minimal impact on the CYP450 system.  Alternatively, consider other classes such as Penicillins or Lincosamides (Clindamycin), keeping in mind the most recent IE prophylaxis guidelines.  Monitoring: If a macrolide like clarithromycin must be used with warfarin, the patient’s INR must be monitored closely, and the warfarin dose may need temporary adjustment by the managing physician.  Conclusion  The inhibitory effect of erythromycin and clarithromycin on the cytochrome P450 system is a major clinical trap. In the context of maxillofacial surgery, where maintaining hemostasis is vital, these interactions can turn a routine procedure into a surgical crisis. Utilizing Azithromycin or performing a comprehensive DDI check is the standard of care to ensure patient safety. | TRUE.  This statement is TRUE. The inhibition of cytochrome P450 (CYP450) enzymes by macrolide antibiotics is one of the most clinically significant and extensively documented drug-drug interactions in pharmacology.  Molecular Mechanism of CYP450 Inhibition  The CYP450 superfamily comprises multiple hepatic and intestinal isoenzymes responsible for phase I oxidative biotransformation of endogenous and exogenous compounds. The CYP3A4 isoenzyme alone accounts for the metabolism of approximately 50% of clinically used drugs, making its inhibition by macrolides pharmacologically consequential for a broad range of co-prescribed medications.  Mechanism of Macrolide CYP3A4 Inhibition:  1. Erythromycin and clarithromycin are oxidised by CYP3A4 to nitrosoalkane metabolites  2. These metabolites form stable, quasi-irreversible complexes with the haem iron moiety of CYP3A4  3. This inactivates the enzyme (mechanism-based/irreversible inhibition), reducing CYP3A4 activity for the remainder of the enzyme's lifespan (irreversible until new enzyme synthesis)  4. The net effect: substantially elevated plasma concentrations of all co-administered CYP3A4-dependent drugs  Spectrum of CYP450 Enzyme Inhibition by Macrolides:  • CYP3A4 — most clinically important  • CYP1A2 — mild inhibition (erythromycin)  • CYP2C9 — mild inhibition (relevant for warfarin metabolism)  Clinically Critical Drug-Drug Interactions Mediated Through CYP450 Inhibition:  \| Drug Class \| Specific Agents \| Consequence \|  \|---\|---\|---\|  \| Statins \| Atorvastatin, simvastatin \| Myopathy, rhabdomyolysis \|  \| Anticoagulants \| Warfarin, acenocoumarol \| Elevated INR, haemorrhage \|  \| Immunosuppressants \| Ciclosporin, tacrolimus \| Nephrotoxicity, organ rejection risk \|  \| Antiepileptics \| Carbamazepine, phenytoin \| CNS toxicity \|  \| Benzodiazepines \| Midazolam, triazolam \| Excess sedation, respiratory depression \|  \| Calcium blockers \| Amlodipine, diltiazem \| Hypotension \|  \| Antiarrhythmics \| Amiodarone, digoxin \| Arrhythmia, digoxin toxicity \|  Comparative CYP3A4 Inhibitory Potency Among Macrolides:  Erythromycin > Clarithromycin >> Azithromycin  Azithromycin's significantly weaker CYP3A4 inhibition profile makes it the preferred macrolide in patients receiving multiple medications with narrow therapeutic indices.  Clinical Recommendation for OMFS Practice:  Before prescribing erythromycin or clarithromycin, always review the patient's complete medication list for CYP3A4 substrates. Consider azithromycin as a safer alternative when significant drug interactions are anticipated. |
| 31. **Ibuprofen is considered the safest and first-choice analgesic during all trimesters of pregnancy for managing severe post-surgical pain in dentistry.** | The statement is incorrect according to current obstetric and pharmacologic safety guidelines.  Ibuprofen is NOT considered safe during all trimesters of pregnancy and is therefore not the first-choice analgesic during all trimesters for managing post-surgical pain.  Trimester-Specific Safety Profile of Ibuprofen:  • First trimester: Controversial; some evidence associates early NSAID use with increased risk of miscarriage and congenital malformations (cardiac and musculoskeletal). Use should be avoided if possible.  • Second trimester: May be used with caution when benefit outweighs risk, but this should be a carefully considered decision.  • Third trimester (after 30 weeks): CONTRAINDICATED. Prostaglandins are essential for maintaining patency of the ductus arteriosus in the fetus. Ibuprofen inhibits COX-mediated prostaglandin synthesis, causing premature closure of the ductus arteriosus, potentially resulting in pulmonary hypertension of the newborn. Additionally, NSAID use in late pregnancy can cause oligohydramnios, renal dysfunction, and prolonged labour.  Appropriate Analgesic in Pregnancy:  Paracetamol (acetaminophen) is the recommended first-line analgesic throughout pregnancy when analgesia is necessary, as it does not carry the fetal risks associated with NSAIDs. Even paracetamol should be used at the lowest effective dose for the shortest necessary duration.  The statement is therefore FALSE. | The statement that Ibuprofen is the safest and first-choice analgesic during all trimesters of pregnancy is clinically and toxicologically incorrect.  Ibuprofen is explicitly contraindicated during the third trimester of pregnancy and should be used with significant caution in the first and second trimesters — it is never the "safest first-choice analgesic" during all trimesters.  Pregnancy Safety Profile of Ibuprofen:  First Trimester (Weeks 1–12):  • Multiple epidemiological studies associate first-trimester NSAID use with:  — Increased risk of spontaneous abortion (miscarriage)  — Potential cardiac septal defects  • Recommendation: Avoid if possible  Second Trimester (Weeks 13–27):  • Most restrictive guidelines consider this a period of limited-duration acceptable use with close monitoring  • Short-course low-dose ibuprofen may be considered when paracetamol is insufficient  Third Trimester (Weeks 28–40): CONTRAINDICATED  • Mechanism: Prostaglandins (PGE2, PGI2) maintain patency of the ductus arteriosus (DA) in utero. Ibuprofen-mediated COX inhibition reduces DA-maintaining prostaglandins, causing:  — Premature ductal closure → neonatal pulmonary hypertension  — Oligohydramnios (reduced fetal urine production)  — Fetal renal dysfunction  — Delayed parturition and prolonged labour  Recommended Alternative Throughout Pregnancy:  Paracetamol (acetaminophen): First-line analgesic in pregnancy at all trimesters; lowest effective dose for shortest duration. | FALSE.  This statement is categorically FALSE and represents a dangerous misconception about analgesic safety in obstetric pharmacology.  Ibuprofen Pregnancy Safety Classification: FDA Classification (historical): Category C (first/second trimester) → Category D (third trimester) Current classification framework: Labelling updated to reflect specific risks across trimesters.  Trimester-Specific Risks of Ibuprofen:  First Trimester (Weeks 0–12): • Prostaglandins are required for successful implantation, maintenance of early pregnancy, and embryonic development • NSAID use in early pregnancy is associated with:  — Increased risk of spontaneous abortion (RR approximately 1.6–2.4 in cohort studies)  — Possible association with cardiac septal defects and other structural anomalies (controversial; mechanistically plausible) • Recommendation: Contraindicated or strictly avoided  Second Trimester (Weeks 13–27): • Considered the lowest-risk window for NSAID use if absolutely required • However, even short-course use should be carefully evaluated; routine use is not endorsed • Some international guidelines permit limited use under specialist supervision  Third Trimester (Weeks 28–Term): ABSOLUTE CONTRAINDICATION The third-trimester contraindication for NSAIDs is based on four well-characterised adverse effects:  1. Premature closure of the ductus arteriosus (DA): • The ductus arteriosus — a fetal vascular connection bypassing the pulmonary circulation — is maintained patent in utero by PGE2 and PGI2 produced via COX-2 • NSAID-mediated COX inhibition suppresses these prostaglandins → premature DA constriction or closure • Consequence: Fetal pulmonary hypertension, right ventricular failure, and neonatal cardiopulmonary compromise  2. Oligohydramnios: • Fetal renal prostaglandins regulate renal vascular tone and tubular function • COX inhibition → reduced fetal urine output → oligohydramnios (potentially severe) • Risk: Fetal limb contractures, pulmonary hypoplasia, umbilical cord compression  3. Impaired renal development: • Prostaglandins are essential for nephrogenesis; NSAID exposure in late pregnancy may cause neonatal renal insufficiency  4. Inhibition of platelet aggregation: • Risk of intrapartum and neonatal haemorrhage  Correct Analgesic for Pregnant Patients in OMFS: Paracetamol 500–1000mg every 4–6 hours (maximum 4g/day) is the ONLY routinely recommended analgesic throughout pregnancy, at the lowest effective dose for the shortest duration necessary. Even paracetamol should be used judiciously, as recent epidemiological data raise questions about prolonged high-dose exposure. |
| 32. **The initial management of severe odontogenic facial space infections (e.g., Ludwig’s Angina) can be effectively managed using oral antibiotics alone.** | The statement is false and contradicts established principles of emergency oral and maxillofacial infection management.  Evidence-Based Clinical Position  Severe odontogenic facial space infections, particularly Ludwig’s angina, represent a life-threatening deep neck space infection characterized by rapidly progressive cellulitis involving the submandibular, sublingual, and submental spaces. The condition carries a significant risk of:  Airway obstruction  Mediastinal spread  Sepsis  Aspiration and sudden respiratory compromise  According to contemporary emergency OMFS and infectious disease guidelines, oral antibiotics alone are not appropriate for initial management.  Why Oral Antibiotics Alone Are Inadequate  Severe odontogenic space infections typically involve:  Polymicrobial flora (aerobic and anaerobic bacteria)  Rapid tissue edema and induration limiting oral absorption  Compromised vascular perfusion of infected tissues  High bacterial load requiring high serum antibiotic concentrations  Oral therapy fails to achieve:  Reliable serum levels in severe systemic infection  Rapid bactericidal effect required in rapidly spreading infections  Control of airway-threatening edema  Standard Evidence-Based Management of Ludwig’s Angina  1. Airway Protection (Priority Intervention)  Early airway assessment is critical  Indications for:  Awake fiberoptic intubation or  Elective tracheostomy in advanced cases  Airway compromise is the leading cause of mortality  2. Hospital Admission and Monitoring  ICU or high-dependency setting  Continuous airway and hemodynamic monitoring  3. Intravenous Broad-Spectrum Antibiotics (Immediate)  Empiric coverage must target:  Streptococci  Staphylococci  Anaerobes (e.g., Prevotella, Fusobacterium)  Common regimens include:  Ampicillin-sulbactam  Clindamycin + metronidazole (if penicillin-allergic)  Piperacillin-tazobactam in severe cases  4. Surgical Source Control  Early incision and drainage of involved fascial spaces  Removal of odontogenic source (usually offending tooth)  Placement of drains when necessary  5. Supportive Care  IV fluids  Pain control  Glycemic control in diabetics  Oxygen therapy if required  Role of Oral Antibiotics  Oral antibiotics may be used only:  During step-down therapy after clinical stabilization  After adequate surgical drainage  When patient is afebrile and infection is resolving  They are not appropriate as initial monotherapy in severe disease.  Clinical OMFS Conclusion  In Ludwig’s angina and other severe odontogenic space infections, delayed or inadequate treatment significantly increases mortality risk. Early aggressive intervention is mandatory.  Final Answer  False. Severe odontogenic facial space infections such as Ludwig’s angina cannot be effectively managed with oral antibiotics alone. Initial management requires urgent airway assessment, intravenous broad-spectrum antibiotics, and surgical drainage, with oral antibiotics reserved only for later step-down therapy after clinical stabilization. | The statement that severe odontogenic facial space infections, such as Ludwig’s Angina, can be effectively managed using oral antibiotics alone is dangerously incorrect and contradicts the fundamental principles of life-saving maxillofacial emergency care.As an Oral and Maxillofacial Surgeon, you recognize that Ludwig’s Angina is a rapidly spreading, potentially fatal cellulitis of the submandibular, submental, and sublingual spaces. The standard of care for such cases necessitates a multi-stage, hospital-based surgical intervention.1. Airway Management: The Absolute PriorityIn the context of Ludwig’s Angina, the floor of the mouth becomes significantly edematous, elevating and displacing the tongue superiorly and posteriorly. This leads to acute upper airway obstruction.Clinical Reality: Oral antibiotics have zero effect on the mechanical obstruction of the airway.Intervention: Management begins with immediate airway stabilization, often requiring fiberoptic intubation or an emergency tracheostomy/cricothyroidotomy if the trismus and tongue displacement preclude standard laryngoscopy.2. Surgical Decompression and DrainageA hallmark of severe space infections is the accumulation of pus or inflammatory exudate under high pressure within the deep fascial planes.I&D Protocol: Treatment requires urgent Incision and Drainage (I&D). This involves aggressive surgical exploration of the submandibular, sublingual, and submental spaces to achieve decompression and establish dependent drainage.Source Control: The offending tooth (the primary source of infection, typically a mandibular second or third molar) must be extracted to prevent recurrence and further inoculation of the spaces.3. Intravenous (IV) vs. Oral Antimicrobial TherapyThe pharmacological management of deep neck infections must bypass the gastrointestinal tract to achieve rapid, high-peak serum concentrations.IV Administration: Patients with severe infections often present with dysphagia (difficulty swallowing) and odynophagia (painful swallowing), making the administration of oral medication physically impossible or unreliable.Empiric Selection: Therapy must be intravenous and broad-spectrum to cover the polymicrobial nature of these infections (aerobic and anaerobic species). Common regimens include:Ampicillin/Sulbactam (3g IV every 6 hours)Clindamycin (600–900 mg IV every 8 hours) — though less common now due to resistance.Ceftriaxone + Metronidazole4. Clinical Progression and MonitoringRelying on oral antibiotics in a healthy or compromised individual with Ludwig’s Angina risks the progression of the infection into the mediastinum (mediastinitis) or the development of sepsis and septic shock.Management PillarLocalized InfectionLudwig's Angina / Severe Space InfectionSettingOutpatientInpatient (ICU/High Dependency)AntibioticsOralIntravenous (IV)AirwayNot compromisedHigh Risk / Emergency StabilizationSurgerySimple ExtractionExtensive I&D + Source ControlConclusionManaging Ludwig’s Angina with oral antibiotics alone constitutes medical malpractice. The condition is a surgical emergency that requires the "Triangle of Treatment": 1) Secure the airway, 2) Surgical incision and drainage for decompression, and 3) High-dose intravenous antibiotic therapy. Clinical success is dependent on immediate hospitalization and aggressive surgical intervention. | FALSE.  This statement is FALSE and represents a critical patient safety issue. Ludwig's Angina is an immediately life-threatening deep neck space infection for which oral antibiotic monotherapy is wholly inadequate and potentially fatal if used as sole management.  Definition and Clinical Characteristics:  Ludwig's Angina is a rapidly progressive bilateral cellulitis of the submandibular, sublingual, and submental spaces, most commonly of odontogenic origin (mandibular second/third molar infections accounting for 70–80% of cases). Key characteristics include:  • Brawny, woody induration of the floor of mouth and neck  • Elevation and posterior displacement of the tongue  • Inability to open the mouth (trismus)  • Dysphagia and drooling  • Respiratory distress (the primary cause of mortality — historically 50%; currently <5% with early intervention)  • Rapid, potentially airway-threatening progression  Why Oral Antibiotics Alone Are Categorically Insufficient:  1. Airway Emergency Takes Absolute Priority:  The life-threatening element of Ludwig's Angina is NOT infection per se — it is airway compromise caused by progressive tongue displacement and floor-of-mouth oedema. Antibiotics cannot reverse this anatomical threat rapidly enough. Early definitive airway management (awake fibre-optic nasotracheal intubation or surgical tracheostomy) is the critical first intervention.  2. Intravenous Antibiotics Are Mandatory:  • Oral bioavailability is unreliable in a systemically ill, dysphagia-affected patient  • IV administration achieves immediate, sustained therapeutic plasma and tissue concentrations  • Tissue penetration is inadequate via oral route in oedematous, hypoperfused deep space tissues  Standard IV Antibiotic Regimens:  Mild-moderate: Amoxicillin-clavulanate 1.2g IV Q8h + metronidazole 500mg IV Q8h  Severe/nosocomial: Piperacillin-tazobactam 4.5g IV Q8h ± vancomycin (MRSA coverage)  Penicillin allergy: Clindamycin + aztreonam, or meropenem  3. Surgical Drainage Is an Integral Component:  Established pus, necrotic tissue, and abscess collections cannot be sterilised by antibiotic therapy alone:  • Surgical incision and drainage of all involved spaces (submandibular, sublingual, submental, and potentially parapharyngeal if extended)  • Parallel incisions intraorally and extraorally for dependent drainage  • Continuous drain placement with wound irrigation  • Repeated surgical exploration if clinical improvement is inadequate  4. Intensive Care Support:  • ICU or high-dependency unit monitoring  • Serial airway assessments  • IV fluid resuscitation  • Nutritional support via nasogastric tube  • Regular wound inspection and microbiological reassessment |
| 33. **Non-steroidal anti-inflammatory drugs (NSAIDs) demonstrate a synergistic effect when combined with low-dose opioids, thereby reducing the total opioid requirement.** | The statement is correct, and it is well supported by contemporary pharmacologic evidence and multimodal analgesia guidelines.  Evidence-Based Clinical Position  Non-steroidal anti-inflammatory drugs (NSAIDs), when combined with opioids in low or moderate doses, demonstrate a clinically relevant opioid-sparing (synergistic) effect. This is a cornerstone of modern multimodal analgesia strategies in oral and maxillofacial surgery and perioperative pain management.  Mechanistic Rationale  The synergistic effect arises from the complementary mechanisms of action:  NSAIDs  Inhibit cyclooxygenase (COX-1 and/or COX-2)  Reduce peripheral prostaglandin synthesis  Decrease:  Inflammatory sensitization of nociceptors  Tissue edema–mediated pain amplification  Opioids  Act centrally via μ-opioid receptors  Modulate pain perception at the spinal and supraspinal levels  Combined Effect  Peripheral + central analgesic pathways are targeted simultaneously  Results in greater analgesic efficacy than either agent alone  Allows reduction in opioid dose without loss of analgesic control  Clinical Evidence  Systematic reviews in postoperative surgical populations (including dental and maxillofacial surgery) consistently show that:  NSAID + opioid combinations provide superior pain control  They significantly reduce:  Total opioid consumption  Opioid-related adverse effects (nausea, vomiting, sedation, constipation)  They improve functional recovery and patient satisfaction  This opioid-sparing effect is a key reason NSAIDs are recommended as first-line agents in acute postoperative dental pain, with opioids reserved for breakthrough pain.  Relevance in Oral and Maxillofacial Surgery  In procedures such as:  Impacted third molar surgery  Orthognathic surgery  Maxillofacial trauma repair  Dental implant surgery with bone manipulation  NSAID–opioid combinations are particularly useful in:  Moderate to severe postoperative pain  Cases where NSAID monotherapy is insufficient  Short-term rescue analgesia protocols  Safety and Clinical Considerations  While effective, the combination must be individualized:  NSAID limitations:  Gastrointestinal risk  Renal impairment risk  Bleeding risk in selected surgical contexts  Opioid limitations:  Respiratory depression (dose-dependent)  Sedation  Nausea/vomiting  Risk of dependence with prolonged use  Therefore, modern guidelines emphasize:  Lowest effective opioid dose  Shortest possible duration  Preferential use of NSAIDs as baseline therapy  Final OMFS Interpretation  The NSAID–opioid combination is not merely additive but functionally synergistic in clinical analgesia, enabling effective pain control with reduced opioid exposure, which is a key goal in current perioperative pain management protocols.  Final Answer  True. NSAIDs exhibit a clinically meaningful opioid-sparing synergistic effect when combined with low-dose opioids, resulting in improved analgesia and reduced total opioid consumption. This principle forms a core component of modern multimodal analgesia strategies in oral and maxillofacial surgical practice. | The statement that Non-Steroidal Anti-Inflammatory Drugs (NSAIDs) demonstrate a synergistic effect when combined with low-dose opioids, effectively reducing the total opioid requirement, is clinically accurate and represents a cornerstone of contemporary Multimodal Analgesia in Oral and Maxillofacial Surgery.This approach is heavily endorsed by the American Association of Oral and Maxillofacial Surgeons (AAOMS) and the World Health Organization (WHO) as part of an "opioid-sparing" strategy to optimize pain management while minimizing the risk of adverse effects, dependency, and the ongoing opioid crisis.1. The Pharmacological Rationale: Synergistic vs. Additive EffectsThe efficacy of this combination stems from the fact that these two classes of drugs target different levels of the pain pathway:NSAIDs (Peripheral Action): These agents (e.g., Ibuprofen, Naproxen, Ketorolac) inhibit the Cyclooxygenase (COX) enzymes, preventing the conversion of arachidonic acid into prostaglandins ($PGE_2$) at the site of surgical trauma. This reduces the sensitization of peripheral nociceptors (peripheral sensitization).Opioids (Central Action): These agents (e.g., Oxycodone, Hydrocodone, Codeine) act as agonists at mu-opioid receptors in the central nervous system (CNS). They modulate the perception of pain and the emotional response to it by altering the descending inhibitory pathways.When combined, these mechanisms provide a synergistic effect, meaning the cumulative analgesic benefit is greater than the sum of their individual effects. This allows the surgeon to achieve superior pain control using significantly lower doses of opioids than would be required if the opioid were used as a monotherapy.2. The "Opioid-Sparing" EffectThe primary clinical advantage of this synergy is the reduction of the total opioid load. In the maxillofacial setting—such as after orthognathic surgery, reconstructive bone grafting, or complex impactions—this reduction is critical for:Mitigating Side Effects: Lowering the dose of opioids reduces the incidence of nausea, vomiting, constipation, respiratory depression, and cognitive impairment (sedation).Preventing Hyperalgesia: Excessive opioid use can paradoxically lead to opioid-induced hyperalgesia; NSAIDs help stabilize the nociceptive threshold.Faster Recovery: Patients with lower opioid intake often experience quicker return of bowel function and faster mobilization post-operatively.3. Clinical Application and GuidelinesCurrent evidence-based protocols for managing acute post-surgical pain in dentistry suggest a "stepped" approach:Tier 1: Standard NSAID (e.g., 400–600 mg Ibuprofen) combined with Paracetamol (1000 mg).Tier 2 (Severe Pain): Add a low-dose opioid (e.g., 5 mg Oxycodone) to the Tier 1 regimen.Tier 3: Schedule the NSAID/Paracetamol combination as the "base" medication every 6–8 hours, utilizing the opioid only as a "breakthrough" rescue medication.4. Specialist Considerations: ContraindicationsWhile synergistic, the surgeon must remain vigilant regarding the contraindications of the NSAID component, particularly:Gastrointestinal: Active peptic ulcer disease or history of GI bleeding.Renal: Significant renal impairment or dehydration (risk of acute tubular necrosis).Hemostasis: Inhibition of platelet aggregation, which may be a concern in extensive reconstructive cases or in patients already on anticoagulants.ConclusionThe combination of NSAIDs and low-dose opioids is an evidence-based standard of care. It utilizes peripheral and central pharmacological synergy to achieve high-quality analgesia while fulfilling the surgical responsibility of opioid stewardship. By prioritizing NSAIDs as the foundational analgesic, the maxillofacial surgeon provides a safer and more effective recovery path for the patient. | TRUE.  This statement is TRUE. The synergistic interaction between NSAIDs and opioids is among the most well-documented and clinically exploited drug interactions in pain pharmacology, forming the mechanistic foundation for multimodal opioid-sparing analgesia.  Pharmacological Basis of NSAID-Opioid Synergism  The pain pathway has multiple distinct anatomical and molecular targets. NSAIDs and opioids act at fundamentally different nodes within this pathway, enabling synergism rather than mere additivity.  Mechanism of NSAIDs:  Peripheral action:  • COX-1/COX-2 inhibition → reduced PGE2, PGI2, thromboxane at injury site  • Decreased sensitisation of peripheral Aδ and C-fibre nociceptors (primary hyperalgesia prevention)  • Reduced inflammatory mediator production (bradykinin, cytokines, substance P amplification)  Spinal action:  • COX-2 upregulation in dorsal horn following peripheral injury is inhibited  • Reduced spinal prostaglandin-mediated amplification of nociceptive signals  Mechanism of Opioids:  Central spinal action:  • μ, κ, δ-opioid receptor agonism at pre- and post-synaptic dorsal horn neurons  • Presynaptic: Inhibits release of substance P, glutamate, CGRP from primary afferent terminals  • Postsynaptic: Hyperpolarises second-order neurons (↑K+ conductance, ↓Ca²+ conductance)  Supraspinal action:  • Periaqueductal grey (PAG) → activation of descending noradrenergic and serotonergic inhibitory systems → gate control at dorsal horn  Why Synergism (Not Just Additivity) Occurs:  The convergence of peripheral prostaglandin reduction (NSAIDs) and central opioid receptor-mediated inhibition acts on independent molecular targets with complementary functional outcomes. Isobolographic analysis (mathematical modelling of drug combinations) demonstrates that the NSAID-opioid analgesic response curves are genuinely synergistic — more than the sum of individual contributions.  Quantified Clinical Opioid-Sparing Effect:  Meta-analyses across surgical and pain settings:  • Ketorolac + morphine: 25–35% morphine sparing  • Ibuprofen + oxycodone: 30–40% oxycodone sparing  • Celecoxib + morphine: 20–30% morphine sparing  • Diclofenac + tramadol: 35–50% tramadol sparing  Reduction in Opioid Adverse Effects at Reduced Doses:  • PONV: 20–30% reduction  • Respiratory depression: Significant risk reduction  • Ileus and constipation: Reduced  • Urinary retention: Reduced  • Sedation: Reduced |
| 34. **The risk of cross-allergy between penicillins and cephalosporins in the general population is estimated to be over 50%.** | The statement is false.  Evidence-Based Clinical Position  The true rate of clinically significant cross-reactivity between penicillins and cephalosporins is very low, and not remotely close to >50%. The historically quoted high rates are based on outdated first-generation cephalosporins with similar side chains and contamination issues in early manufacturing, not on modern pharmacologic evidence.  Current Estimated Cross-Reactivity Risk  Contemporary systematic reviews and allergy literature demonstrate:  Overall cross-reactivity: ~1–2%  Higher risk only with:  First-generation cephalosporins  Cephalosporins sharing similar R1 side chains with penicillins  Third- and fourth-generation cephalosporins: <1% risk, often clinically negligible  Thus, the true risk is low and structurally dependent, not population-wide or fixed.  Mechanistic Explanation  Cross-reactivity is primarily driven by:  Similar R-side chain structures (not the β-lactam ring itself)  Immunologic recognition of shared antigenic determinants  Modern cephalosporins have:  Distinct side chains  Improved purity and manufacturing standards  → significantly reduced immunogenic overlap  Clinical Implications in Oral and Maxillofacial Surgery  This is highly relevant when selecting antibiotics for:  Dental infections  Surgical prophylaxis  Odontogenic space infections  Practical guideline-based approach:  Patients with non-anaphylactic penicillin allergy (e.g., rash history):  → Many cephalosporins can be safely used (especially later generations)  Patients with true immediate IgE-mediated anaphylaxis:  → Avoid structurally similar β-lactams  → Prefer alternatives such as:  Clindamycin (context-dependent, with current stewardship caution)  Azithromycin / clarithromycin  Doxycycline  Why the “>50%” figure is incorrect  That number originates from:  Early studies from the 1960s–1970s  Contaminated cephalosporin preparations with penicillin residues  Lack of structural classification of side-chain immunogenicity  Modern immunologic and epidemiologic data have definitively disproven it.  Final Answer  False. The risk of cross-allergy between penicillins and cephalosporins is not >50%. Contemporary evidence shows a low overall risk (~1–2%), largely dependent on similarity of side-chain structures rather than class-wide cross-reactivity. Modern cephalosporins, particularly second- and third-generation agents, have a very low clinically relevant cross-allergy risk. | The statement that the risk of cross-allergy between penicillins and cephalosporins is estimated to be over 50% is factually incorrect and represents an outdated medical misconception that has been debunked by contemporary pharmacological and immunological research.As an Oral and Maxillofacial Surgeon, maintaining an accurate understanding of cross-reactivity is vital for ensuring safe and effective antibiotic selection, particularly when managing odontogenic infections or providing perioperative prophylaxis.1. Historical Context vs. Modern EvidenceThe "10% cross-reactivity" myth was largely based on early studies from the 1960s and 1970s. During that period, cephalosporins (specifically first-generation agents like cephalothin) were produced using Penicillium molds, leading to trace contamination with penicillin.Modern manufacturing processes have eliminated this contamination. Recent literature and systematic reviews indicate that the true rate of cross-reactivity between penicillins and cephalosporins in the general population is significantly lower, typically estimated to be between 1% and 3%. In patients with a confirmed (IgE-mediated) penicillin allergy, the risk of a reaction to a third- or fourth-generation cephalosporin is negligible (less than 1%).2. The Role of the R1 Side ChainImmunological cross-reactivity is primarily determined by the R1 side chain of the chemical structure rather than the common beta-lactam ring.Structural Similarities: Cross-reactions are most likely to occur between drugs that share identical or highly similar R1 side chains. For example, Amoxicillin and Cephalexin share an identical side chain (p-hydroxybenzyl), which increases the theoretical risk of cross-reactivity compared to other pairings.Generation-Dependent Risk: First-generation cephalosporins (e.g., Cephalexin) share more structural similarities with penicillins than later generations. Third- and fourth-generation cephalosporins (e.g., Ceftriaxone, Cefepime) have distinct side chains, making them exceptionally safe even in penicillin-allergic patients.3. Clinical Implications for Maxillofacial SurgeryWhen treating a patient with a reported penicillin allergy, a nuanced approach is required based on the nature of the previous reaction:Non-Anaphylactic History (e.g., simple rash, GI upset): Cephalosporins (including first-generation) can generally be administered safely. The benefit of using a cephalosporin often outweighs the minimal risk of a minor skin reaction.IgE-Mediated Anaphylaxis History (e.g., angioedema, bronchospasm, urticaria): Cephalosporins should be used with extreme caution. While the risk remains low with later-generation agents, the standard of care is to select a non-beta-lactam alternative such as Azithromycin or Doxycycline (notably, Clindamycin has been removed from recent AHA IE prophylaxis guidelines due to C. diff risks).4. Patient Risk Stratification TablePenicillin Reaction HistoryCephalosporin Administration RecommendationLow Risk (Gastrointestinal upset, headache)Safe to administer.Moderate Risk (Delayed skin rash, no systemic symptoms)Safe to administer; monitor for minor reactions.High Risk (Anaphylaxis, Laryngeal edema, Hypotension)Avoid cephalosporins (especially 1st/2nd Gen); select different antibiotic class.ConclusionThe claim of >50% cross-reactivity is a significant overestimation that can lead to "penicillin allergy labeling," resulting in the use of less effective or more toxic alternative antibiotics. Current evidence supports that the vast majority of penicillin-allergic patients can safely tolerate cephalosporins. In the maxillofacial setting, accurate risk stratification ensures optimal source control of infections while minimizing the potential for adverse drug events. | FALSE.  This statement is FALSE. The estimated cross-reactivity rate between penicillins and cephalosporins in the general population is approximately 1–2%, not over 50%.  Historical Background and the Origins of the Overestimation:  The frequently cited 10% cross-reactivity estimate originated from three sources of methodological error in early studies (1960s–1970s):  1. Penicillin contamination of early cephalosporin batches: Early manufacturing processes resulted in cephalosporin preparations containing penicillin residues, which artifactually elevated apparent cross-reactivity rates  2. Methodological limitations: Skin testing and clinical assessment methods in early studies lacked sensitivity and specificity  3. Selection bias: Studies often enrolled patients with the most severe allergy histories  Contemporary Evidence — Revised Estimate:  Prospective studies using purified cephalosporin preparations and standardised allergy evaluation (skin testing + graded oral challenge):  • Macy & Romano (JACI, 2014): Cross-reactivity in penicillin-allergic patients confirmed by challenge: <1%  • Blumenthal et al. (JAMA Int Med, 2018): Cross-reactivity between amoxicillin and cephalosporins: ~1%  • Multiple systematic reviews: Overall cross-reactivity 1–2%  Molecular Basis — Side Chain Hypothesis:  The current understanding is that beta-lactam cross-reactivity is mediated primarily by similar or identical R1 side chains, not by the shared beta-lactam ring:  High cross-reactivity pairs (shared R1 side chain):  • Amoxicillin ↔ Cefadroxil, Cefprozil (identical R1 aminobenzyl group)  • Ampicillin ↔ Cefaclor, Cefalexin  Low cross-reactivity pairs (dissimilar R1 side chains):  • Penicillin V ↔ Ceftriaxone, Cefuroxime, Ceftazidime  Clinical Implications for OMFS Practice:  The historically overestimated cross-reactivity rate led to widespread denial of cephalosporin therapy to penicillin-labelled patients, exposing them instead to less effective or more toxic alternatives. Current guidelines (EAACI, AAAAI/ACAAI, NICE) now recommend:  1. Formal evaluation of penicillin allergy history (most reported allergies are not true IgE-mediated reactions)  2. Cephalosporins may generally be used in penicillin-allergic patients, particularly if:  — Allergy history does not suggest anaphylaxis  — Cephalosporin with dissimilar R1 side chain is selected  3. Graded challenge or skin testing for confirmed IgE-mediated penicillin allergy before cephalosporin administration |
| 35. **Submucosal or intramuscular administration of corticosteroids (e.g., dexamethasone) prior to third molar surgery is effective in reducing postoperative edema and trismus.** | The statement is correct.  Evidence-Based Clinical Position  Preoperative administration of corticosteroids, including dexamethasone via submucosal, intramuscular, or intravenous routes, is an evidence-supported strategy to reduce postoperative inflammatory sequelae following surgical removal of impacted third molars.  This intervention is widely incorporated into multimodal perioperative protocols in oral and maxillofacial surgery.  Mechanism of Action  Dexamethasone exerts potent anti-inflammatory effects by:  Inhibiting phospholipase A₂, thereby suppressing arachidonic acid cascade  Reducing synthesis of:  Prostaglandins  Leukotrienes  Stabilizing lysosomal membranes  Decreasing capillary permeability  Attenuating cytokine-mediated inflammatory response (e.g., IL-1, TNF-α)  These effects directly reduce:  Soft tissue edema  Trismus (secondary to inflammatory muscle involvement)  Postoperative pain (indirectly via reduced inflammation)  Evidence from Oral Surgery Literature  Multiple randomized controlled trials and meta-analyses in third molar surgery demonstrate that:  Preoperative corticosteroids significantly reduce:  Facial swelling (peak at 48–72 hours post-op)  Maximal interincisal opening limitation (trismus)  Pain scores (modest but consistent reduction)  Timing matters: preoperative administration is more effective than postoperative dosing due to inhibition of early inflammatory cascade activation  Route of Administration  1. Submucosal (local infiltration near surgical site)  Simple and widely used in dental practice  Provides high local tissue concentration  Effective in reducing localized inflammation  2. Intramuscular (e.g., deltoid muscle)  More systemic distribution  Useful when intraoral injection is not preferred  Predictable pharmacokinetics  3. Intravenous  Most rapid onset  Often used in hospital-based or complex surgical settings  Clinical Considerations  Typical dexamethasone dosing: 4–8 mg single preoperative dose  Most effective when administered 30–60 minutes before surgery  Generally safe as a single dose in healthy patients  Contraindications / cautions:  Poorly controlled diabetes mellitus (transient hyperglycemia)  Active systemic infection (relative caution)  Peptic ulcer disease (with repeated dosing, not single dose)  Immunosuppression considerations in repeated use  Clinical OMFS Implication  In impacted third molar surgery, corticosteroid use is particularly beneficial in:  Horizontally or deeply impacted teeth requiring flap elevation and bone removal  Longer surgical duration cases  Patients with high inflammatory response tendency  They are commonly combined with:  NSAIDs (multimodal analgesia)  Atraumatic surgical technique  Cold therapy postoperatively  Final Answer  True. Preoperative administration of corticosteroids such as dexamethasone via submucosal or intramuscular routes is evidence-based and effective in reducing postoperative edema and trismus following third molar surgery, primarily through suppression of the early inflammatory cascade and reduction of tissue permeability. | The statement that submucosal or intramuscular administration of corticosteroids, specifically Dexamethasone, prior to third molar surgery is effective in reducing postoperative edema and trismus is clinically accurate and strongly supported by a high level of evidence in the maxillofacial literature.As an Oral and Maxillofacial Surgeon, you are aware that the extraction of impacted mandibular third molars involves significant tissue manipulation and ostectomy, which triggers a robust inflammatory cascade. The proactive use of corticosteroids is a standard of care to modulate this response.1. Pharmacological Mechanism: The Genomic EffectCorticosteroids like Dexamethasone are potent synthetic glucocorticoids that act early in the inflammatory pathway. Their primary mechanism involves:Phospholipase $A_2$ Inhibition: By inducing the synthesis of lipocortin-1, dexamethasone inhibits the enzyme phospholipase $A_2$. This prevents the release of arachidonic acid from cell membranes, thereby blocking the production of both prostaglandins and leukotrienes.Vascular Permeability Reduction: They stabilize lysosomal membranes and decrease capillary permeability, which directly limits the extravasation of fluid into the interstitial space—the primary cause of post-surgical edema.Cytokine Suppression: They reduce the expression of pro-inflammatory cytokines such as $IL\text{-}1\beta$ and $TNF\text{-}\alpha$, which are implicated in muscle irritation and subsequent trismus (limited jaw opening).2. Routes of Administration and Comparative EfficacyDexamethasone is favored in maxillofacial surgery due to its high potency (approximately 25–30 times that of hydrocortisone) and lack of mineralocorticoid (salt-retaining) activity.Submucosal (SM) Injection: Often administered near the surgical site (e.g., in the buccal fold or masseteric region). This route provides high local concentrations and has been shown to be equally effective, if not superior, to systemic routes for localized swelling.Intramuscular (IM) Injection: Usually administered in the deltoid or gluteal muscle. This provides a steady systemic release and is highly effective for bilateral or complex cases.Clinical Timing: The "pre-emptive" approach (administration 30–60 minutes prior to the first incision) is critical. This ensures that the steroid is biologically active at the genomic level before the inflammatory mediators are synthesized.3. Impact on Postoperative OutcomesEvidence-based systematic reviews and meta-analyses consistently report the following:Edema Control: Significant reduction in facial swelling, typically peaking between 48 and 72 hours postoperatively.Trismus Reduction: A measurable increase in maximum incisal opening (MIO) during the first 3 to 7 days compared to placebo groups.Pain Management: While not a primary analgesic, the reduction in tissue tension and pressure (due to decreased edema) contributes to a significant reduction in the patient's perceived pain levels.4. Clinical Safety and ContraindicationsIn the context of a single-dose or short-term perioperative protocol (e.g., 4 mg to 8 mg of Dexamethasone), the risk of suppressing the hypothalamic-pituitary-adrenal (HPA) axis is negligible. However, surgeons must exercise caution in patients with:Uncontrolled Diabetes Mellitus: Corticosteroids can induce transient hyperglycemia.Active Infections: Potentially masking or exacerbating acute suppuration.Peptic Ulcer Disease: Increased risk of GI irritation, especially when combined with NSAIDs.ConclusionThe pre-emptive administration of Dexamethasone via submucosal or intramuscular routes is a technically profound and evidence-based strategy. It significantly enhances the patient’s postoperative quality of life by mitigating the physiological sequelae of surgical trauma. In contemporary practice, this protocol is an essential adjunct to skillful surgical technique and multimodal analgesia. | TRUE.  This statement is TRUE. Perioperative corticosteroid administration is among the most evidence-supported interventions for reducing post-surgical oedema and trismus in oral and maxillofacial surgery, with a particularly robust evidence base from third molar surgery.  Pharmacological Mechanism  Corticosteroids — primarily dexamethasone in the surgical context — mediate anti-inflammatory effects through two temporally distinct pathways:  Genomic (Primary) Mechanism:  1. Dexamethasone crosses cell membranes and binds intracytoplasmic glucocorticoid receptors (GR-α)  2. Drug-receptor complex translocates to the nucleus  3. Binds glucocorticoid response elements (GREs) → transactivation of anti-inflammatory genes  4. Induces lipocortin-1 (annexin A1) synthesis → inhibits phospholipase A2 → blocks arachidonic acid release from cell membrane phospholipids  5. Consequence: Simultaneous suppression of BOTH the COX pathway (prostaglandins) AND the LOX pathway (leukotrienes) — broader anti-inflammatory effect than NSAIDs alone  6. Additionally: Transrepression of NF-κB → suppressed IL-1β, IL-6, TNF-α, and ICAM-1 expression  Non-Genomic (Rapid) Mechanism:  • Direct membrane stabilisation occurs within minutes  • Rapid attenuation of histamine-mediated and bradykinin-mediated vascular permeability  Anti-Oedema Effect in OMFS:  By simultaneously inhibiting:  • Vascular permeability (histamine, bradykinin, PGI2)  • Inflammatory cytokine-driven fluid extravasation  • Leukocyte margination and diapedesis  Corticosteroids substantially reduce fluid accumulation in peri-surgical facial spaces, limiting the magnitude of postoperative facial swelling.  Anti-Trismus Effect:  Reduced oedema in the pterygomandibular, masseteric, and medial pterygoid spaces:  • Decreases mechanical restriction to jaw opening  • Reduces pressure on masticatory muscles  • Attenuates masticatory muscle inflammatory response  Evidence Summary:  Systematic reviews and meta-analyses (Markiewicz et al., JOMS; Roy & Bhagawati, J Oral Maxillofac Res; Cochrane-level reviews):  Oedema reduction: 30–50% reduction in facial swelling measurements  Trismus improvement: Significantly improved interincisal opening at 24 and 48 hours post-surgery  Pain reduction: Modest but significant reduction in pain intensity  Safety: No significant increase in infection, dry socket, or wound healing complications at standard single pre-operative doses  Optimal Dosing Protocol:  • Drug: Dexamethasone (preferred over betamethasone, methylprednisolone for anti-oedema)  • Dose: 4–8mg (adults)  • Route: Submucosal injection, IM, or IV  • Timing: Pre-operatively (30 min before incision) or intraoperatively at time of flap closure  • Duration: Single dose is standard; two-dose protocols (pre- and post-operative) may provide marginal additional benefit  Contraindications/Precautions:  • Active infection beyond surgical site (relative contraindication)  • Uncontrolled diabetes mellitus (transient hyperglycaemia)  • Known adrenal suppression  • Active peptic ulceration (increased GI risk if combined with NSAIDs) |
| 36. **Irrigating the surgical site with chlorhexidine gluconate is more effective than systemic antibiotic therapy alone in reducing the risk of alveolar osteitis (dry socket).** | The statement is correct.  Chlorhexidine gluconate irrigation of the surgical site has demonstrated efficacy in reducing the incidence of alveolar osteitis (dry socket) following tooth extraction and is considered more effective than systemic antibiotic therapy alone in preventing this complication.  Evidence Base:  Multiple randomised controlled trials and systematic reviews have evaluated chlorhexidine's role in alveolar osteitis prevention. The key findings:  • Preoperative and postoperative chlorhexidine 0.12–0.2% mouth rinsing significantly reduces alveolar osteitis incidence  • Chlorhexidine gel/irrigation applied directly to the extraction socket at time of surgery further reduces risk  • The absolute risk reduction from chlorhexidine is comparable to or exceeds that of prophylactic systemic antibiotics in healthy patients  Why Chlorhexidine is More Effective Than Systemic Antibiotics Alone for Dry Socket:  Alveolar osteitis is not primarily an infectious disease — it is a localised failure of blood clot stabilisation and organisation, partially mediated by local fibrinolysis and bacterial contamination of the socket. Since the pathogenesis is largely local, systemic antibiotics have limited penetration into the avascular socket environment and cannot adequately suppress local bacterial activity or fibrinolysis. Chlorhexidine, as a directly applied broad-spectrum antiseptic, reduces local bacterial burden at the socket site directly.  Mechanism of Chlorhexidine:  • Binds to bacterial cell membranes, disrupting permeability and causing cell death  • Binds to oral mucosal surfaces providing substantivity (prolonged activity up to 12 hours)  • Reduces the total bacterial load that colonises the extraction socket  The statement is therefore TRUE. | The statement is correct.  1. Comparative Efficacy and NNT  Systematic reviews and meta-analyses, including high-level evidence from the Cochrane Library, indicate the following:  Chlorhexidine Gluconate: Both 0.12% and 0.2% CHX (used as a perioperative rinse or as a 1% gel placed directly into the socket) have a low Number Needed to Treat (NNT). Studies suggest that using CHX can reduce the risk of AO by approximately 20% to 50%.  Systemic Antibiotics: While systemic antibiotics (such as Amoxicillin or Metronidazole) also reduce the risk of AO, the NNT is often higher than that of local measures. Furthermore, the risk-to-benefit ratio is frequently unfavorable in healthy (ASA I/II) patients due to the potential for systemic side effects and the promotion of antimicrobial resistance.  2. Pathophysiological Rationale  The primary etiology of Alveolar Osteitis is the premature fibrinolysis of the blood clot within the extraction socket, largely driven by bacterial activators (plasminogen activators) and host-derived factors.  Local Action of CHX: Chlorhexidine is a broad-spectrum antiseptic that targets the oral microflora (specifically Treponema denticola and other anaerobes) implicated in the fibrinolytic process. By reducing the local bacterial load directly at the surgical site, it preserves the integrity of the clot without systemic interference.  Systemic Antibiotic Limitation: Systemic agents require adequate blood flow to reach therapeutic concentrations in the alveolar bone. In the context of an extraction socket, where the primary concern is the stability of a localized clot, the systemic route is often less efficient than direct topical application.  3. Clinical Application: The 1% Gel Protocol  Recent international clinical guidelines increasingly favor the use of CHX 1% bioadhesive gel over systemic prophylaxis for third molar surgery:  Placement: Following the extraction and debridement of the socket, the 1% gel is applied directly into the alveolar space.  Suturing: The socket is often sutured to retain the gel and stabilize the initial clot.  Outcome: This method has shown superior efficacy in reducing AO compared to rinses alone, as it provides a sustained-release effect during the critical first 24–48 hours of healing.  4. Recommendation for the Oral Surgeon  For healthy individuals undergoing surgical extraction of impacted third molars, the evidence-based protocol should be:  First-line: Topical Chlorhexidine (0.12% rinse or 1% gel) is the preferred preventive measure due to its localized action and superior safety profile.  Second-line: Systemic antibiotics should be reserved only for cases where there is a clear pre-existing infection (acute pericoronitis, cellulitis) or if the patient is systemically compromised (e.g., poorly controlled diabetes, immunosuppression).  Conclusion  In the context of alveolar osteitis prevention, local measures (CHX) are both highly effective and clinically preferable to systemic antibiotics. This approach provides excellent clinical outcomes while upholding the ethical and professional responsibility to mitigate systemic antibiotic overuse in oral surgery. | TRUE.  This statement is TRUE and is supported by well-conducted randomised controlled trials and systematic reviews demonstrating the superiority of local chlorhexidine application over systemic antibiotic prophylaxis alone in reducing the incidence of alveolar osteitis.  Alveolar Osteitis: Definition, Incidence, and Aetiology  Definition: Alveolar osteitis (dry socket, localized osteitis, fibrinolytic alveolitis) is characterised by loss of the post-extraction blood clot, with exposure of alveolar bone in the tooth socket, accompanied by severe local pain, halitosis, and bad taste, typically occurring 2–4 days post-extraction.  Incidence:  • Routine extractions: 0.5–5%  • Mandibular third molars: 5–30% (higher with surgical extraction)  • Risk factors: Tobacco smoking, oral contraceptive use, traumatic extraction, inadequate socket irrigation, mandibular location, female sex  Pathophysiology:  Alveolar osteitis develops through two primary mechanisms:  1. Fibrinolytic cascade activation: Bacterial and salivary kinases activate plasminogen to plasmin, which degrades the fibrin clot → socket exposure  2. Bacterial contamination: Oral bacteria (particularly Streptococcus and Actinomyces species) produce inflammatory mediators and fibrinolytic enzymes that perpetuate clot dissolution  Why Chlorhexidine is More Effective Than Systemic Antibiotics for Dry Socket:  Systemic Antibiotic Limitations:  • Must penetrate into an avascular, fibrin-clot-dependent wound environment  • Inadequate concentrations achieved in the socket lumen  • Do not address the fibrinolytic component of pathogenesis  • Cannot prevent the initial bacterial colonisation of the wound surface  Chlorhexidine Gluconate — Mechanism of Efficacy:  1. Concentration at site of action: Direct local application delivers clinically effective concentrations (0.12–0.2%) immediately to the wound surface  2. Broad-spectrum antimicrobial activity: Bactericidal against Gram-positive and Gram-negative bacteria, fungi, and some viruses via membrane disruption  3. Substantivity: Chlorhexidine binds electrostatically to oral mucosal proteins and is slowly released over 6–12 hours, providing sustained antimicrobial activity at the wound site  4. Pre-operative decontamination: Pre-operative rinsing significantly reduces the total oral bacterial load available to colonise the wound at the time of extraction  Clinical Evidence Summary:  Systematic reviews including Cochrane analyses (Rodríguez-Pérez et al., 2018):  • 0.12% chlorhexidine rinse (pre- and post-operative): RR for dry socket = 0.52 [95% CI: 0.35–0.79]  • Chlorhexidine gel in socket: RR for dry socket = 0.41 [95% CI: 0.22–0.76]  • Meta-analyses confirm number-needed-to-treat of approximately 8–12 patients to prevent one dry socket  Optimal Chlorhexidine Protocol for Third Molar Surgery:  1. Pre-operative rinse: 0.12–0.2% chlorhexidine × 60 seconds, 1 minute before procedure  2. Socket irrigation: Chlorhexidine solution used for socket wound lavage at end of procedure  3. Post-operative rinse: 0.12–0.2% chlorhexidine BID × 7 days (beginning day 2 post-extraction to avoid clot disruption on day 1)  This protocol significantly outperforms systemic antibiotic prophylaxis in preventing alveolar osteitis, while avoiding all risks associated with antibiotic use (resistance, C. difficile, adverse reactions). |
