# Supplementary material for: Do large language models differ in their pharmacology-related response quality for oral and maxillofacial surgery? a blinded expert benchmark study
Source: BMC Oral Health. 2026 Jul 4;26:1808. doi: 10.1186/s12903-026-09182-w (PMC13602642; doi:10.1186/s12903-026-09182-w)
Supplement: Supplementary file 2 [file 12903_2026_9182_MOESM2_ESM.docx]

| **Category** | **Score** | **Parameters** | | |
| --- | --- | --- | --- | --- |
|  |  | **Accuracy** | **Comprehensiveness** | **Usefulness** |
| **Open-ended questions** | 1 | The response contains predominantly incorrect or outdated clinical information; contradicts current international guidelines; includes potentially harmful recommendations. | The response addresses only a single aspect of the question; critical clinical information is entirely absent; wholly inadequate coverage. | The response provides no actionable clinical information; cannot be applied in a clinical setting; purely theoretical or irrelevant. |
|  | 2 | The response contains several factual errors or significant omissions; partially inconsistent with current guidelines; requires substantial correction. | The response covers some relevant aspects but omits multiple clinically important elements; significant gaps in content. | The response has limited clinical applicability; information is too vague or generic to guide clinical decision-making. |
|  | 3 | The response is mostly accurate but contains minor factual errors or outdated references; generally consistent with guidelines but not fully reliable. | The response addresses the main points of the question but lacks depth in several areas; moderately complete. | The response provides some clinically useful information but lacks specificity; partially applicable to real clinical scenarios. |
|  | 4 | The response is accurate and consistent with current international guidelines; minor imprecisions present but do not affect clinical applicability. | The response comprehensively covers the topic with minor omissions; most clinically relevant aspects are adequately addressed. | The response provides clinically applicable information with clear practical guidance; suitable for direct use in most clinical scenarios. |
|  | 5 | The response is completely accurate, fully consistent with the most recent international clinical guidelines; all drug dosages, indications, and contraindications are correct. | The response provides an exhaustive, well-structured answer covering all clinically relevant aspects including indications, contraindications, drug interactions, dosages, and evidence base. | The response provides highly specific, immediately actionable clinical guidance; directly applicable to real patient management with clear decision-making support. |
|  |  | **Correctness** | **Accuracy of Justification** | **Usefulness of Explanation** |
| **MCQ questions** | 1 | Incorrect answer selected with no rational basis; reflects fundamental misunderstanding of the topic. | Explanation is factually incorrect or misleading; contradicts established clinical knowledge. | Explanation adds no clinical value; does not clarify why the correct answer is appropriate. |
|  | 2 | Incorrect answer selected but the chosen option demonstrates partial clinical reasoning. | Explanation contains several factual errors; only partially consistent with current guidelines. | Explanation provides minimal clinical insight; insufficient to support clinical reasoning. |
|  | 3 | Correct answer selected but without clear confidence or with contradictory accompanying statements. | Explanation is mostly correct but contains minor inaccuracies or oversimplifications. | Explanation provides adequate reasoning but lacks depth or clinical context. |
|  | 4 | Correct answer selected with adequate justification; minor reasoning gaps present. | Explanation is accurate and well-grounded; consistent with current guidelines with only minor imprecisions. | Explanation enhances understanding of the correct answer with clear clinical rationale; mostly applicable. |
|  | 5 | Correct answer selected with complete certainty; fully consistent with current evidence-based guidelines. | Explanation is completely accurate, evidence-based, and correctly references current international guidelines and pharmacological principles. | Explanation provides comprehensive clinical reasoning, clearly differentiates correct from incorrect options, and directly supports evidence-based clinical decision-making. |
|  |  | **Correctness** | **Accuracy of Justification** | **Usefulness of Justification** |
| **T/F questions** | 1 | Incorrect True/False determination with no rational basis; reflects fundamental misunderstanding. | Justification is factually incorrect, misleading, or entirely absent. | Justification provides no clinical insight; does not contribute to understanding of the statement. |
|  | 2 | Incorrect determination but with partial clinical reasoning that is not entirely without merit. | Justification contains significant factual errors; partially inconsistent with established evidence. | Justification provides minimal clinical value; too vague to support clinical reasoning. |
|  | 3 | Correct determination reached but with ambiguous, inconsistent, or poorly justified reasoning. | Justification is mostly accurate but contains minor errors or lacks reference to current guidelines. | Justification provides moderate clinical insight but lacks specificity or practical applicability. |
|  | 4 | Correct determination with adequate supporting rationale; minor gaps in reasoning. | Justification is accurate and consistent with current evidence; minor imprecisions do not affect overall validity. | Justification provides clear clinical reasoning and is applicable to real clinical scenarios with minor limitations. |
|  | 5 | Correct determination with complete confidence; fully consistent with current international clinical guidelines. | Justification is completely accurate, evidence-based, and explicitly referenced to current international guidelines. | Justification provides comprehensive, clinically applicable reasoning that directly supports evidence-based practice and enhances understanding of the clinical topic. |
